# Spironaphthoxazine switchable dyes for biological imaging†
†Electronic supplementary information (ESI) available: Synthetic protocols, DFT calculations, crystal structure, and additional photo-physical and microscopy characterization. CCDC 1812758. For ESI and crystallographic data in CIF or other electronic format see DOI: 10.1039/c8sc00130h


**DOI:** 10.1039/c8sc00130h

**Published:** 2018-02-20

**Authors:** Yaoyao Xiong, Andreas Vargas Jentzsch, Johannes W. M. Osterrieth, Erdinc Sezgin, Igor V. Sazanovich, Katharina Reglinski, Silvia Galiani, Anthony W. Parker, Christian Eggeling, Harry L. Anderson

**Affiliations:** a Department of Chemistry , University of Oxford , Chemistry Research Laboratory , Oxford OX1 3TA , UK . Email: harry.anderson@chem.ox.ac.uk; b MRC Human Immunology Unit , Weatherall Institute of Molecular Medicine , University of Oxford , OX3 9DS , Oxford , UK; c Central Laser Facility , Research Complex at Harwell, Science and Technology Facilities Council , Harwell Campus , Didcot OX11 0QX , UK; d Institute of Applied Optics , Friedrich-Schiller-University Jena , Jena , Germany; e Leibniz Institute of Photonic Technology e.V. , Jena , Germany

## Abstract

Recent developments in super-resolution microscopy have significantly expanded the requirements for switchable dyes, leading to demand for specially designed molecular switches. We report the synthesis and characterization of a spironaphthoxazine photochromic switch (a derivative of palatinate purple) displaying high photoconversion (85–95%) under readily accessible 405 nm light, broad absorption in the visible, and excellent fatigue resistance. The indole substituent on this spironaphthoxazine is twisted out of conjugation with the naphthalene unit, yet it is crucial for activation with visible light. The open colored merocyanine form of the spironaphthoxazine reverts to the closed form with a lifetime of 4.7 s in dichloromethane at 20 °C; this thermal reversion is even faster in more polar solvents. The photochemical quantum yields for ring-opening and ring-closing are approximately 8% and 1%, respectively, in dichloromethane. The ring-opening and ring-closing reactions have been characterized by time-resolved infrared and transient absorption spectroscopies. Ring opening occurs rapidly (*τ* = 2.1 ns) and efficiently (∼90%) from the singlet excited state to form an intermediate (assigned as a cisoid merocyanine), which returns to the closed ground state (*τ* = 4.5 ns) in competition with relaxation to the transoid open form (*τ* = 40 ns). Photochemical ring closing is a faster and simpler process: the excited state proceeds to the closed spirooxazine with a time constant of 0.28 ns. This photochromic switch can be used in conjunction with commercial fluorescent dyes to create a small-molecule switchable fluorescent dyad that shows high contrast and good fatigue resistance in living cells. These properties make the dyads suitable for application in RESOLFT microscopy.

## Abstract 2

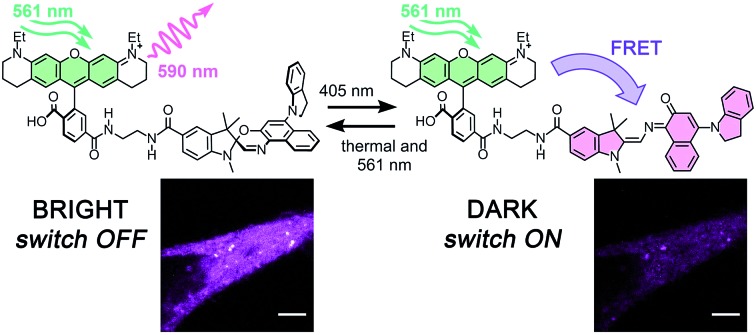
We demonstrate that a photochromic spironaphthoxazine switch operates with excellent fatigue resistance and high conversion when irradiated at 405/561 nm in a range of media including living cells.

## Introduction

Fluorescent labeling techniques have greatly improved recently, particularly with the introduction of fluorescent proteins,^1^ highly optimized small-molecule fluorescent dyes^2^ and advanced tagging techniques.^3^ Not only have the obvious characteristics been improved (*e.g.* brightness, photostability, membrane permeability) but new properties have been introduced (*e.g.* photoactivation^4^,^5^ and environment sensitivity^6^).

Advances in fluorescent-probe engineering are the foundation of one of the most important recent developments in optical microscopy, super-resolution microscopy (SRM). The possibility to alternate between two different fluorescence states is the defining requirement of most current approaches to SRM, now enabling cellular optical imaging with spatial resolution beyond the classical diffraction limit.^7^–^9^

**Fig. 1:**
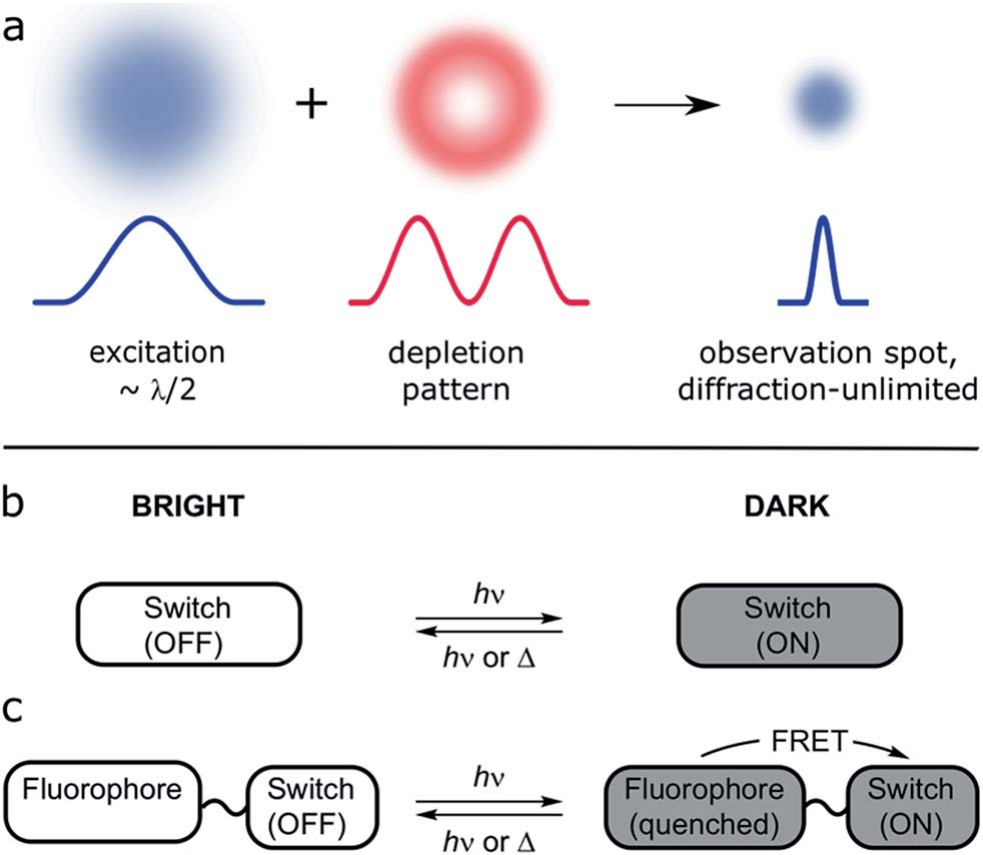
(a) Principle of RESOLFT microscopy: the diffraction-limited excitation spot (Gaussian-shaped, width *λ*/2, where *λ* is the wavelength of the light) is superimposed with a fluorescence-depletion spot with a local intensity minimum (doughnut-shaped intensity distribution). Provided the depletion beam deactivates the fluorescence of the dye, any fluorescence must come from the diffraction unlimited difference spot. (b and c) Two approaches to small-molecule switchable fluorescent dyes: (b) the molecular switch acts as fluorophore itself. (c) The emission of a bright fluorophore can be modulated by a covalently attached photochromic switch in a dyad (*e.g.* quenching by FRET).

A collection of SRM techniques circumvent the diffraction limit and allow improved spatial resolution, but often at the expense of long acquisition times and/or high laser intensities.^10^ Many approaches have been proposed to achieve routine SRM of living systems; one of them is reversible saturable optical fluorescence transitions (RESOLFT) microscopy (Fig. 1a).^9^–^12^ This technique replaces the photophysical switching of stimulated emission depletion (STED) by a photochemical process, so that the depletion and emission processes are no longer in direct competition, allowing use of lower laser intensities.

The first working examples of RESOLFT microscopy were made possible by the development of stable switchable fluorescent proteins.^9^,^11^,^13^ However, the use of fluorescent proteins is not always desirable (for example this strategy cannot be used to label endogenous proteins) and therefore significant effort has been devoted to the development of small-molecule switchable fluorescent dyes for use in RESOLFT microscopy.^14^–^16^ Surprisingly, although photochromic molecular switches have been studied for more than 100 years,^17^ only limited success toward this application has been reported. This impasse reflects the demanding list of requirements that such a dye must fulfill to be suitable for RESOLFT. Specifically, these requirements are:

(1) A stable emissive form (μs–ms lifetime) that can be switched off photochemically using visible light.

(2) High contrast of bright and dark states, which is mainly determined by the darkness of the dark state. The composition of the photo-stationary state (PSS) is crucial for achieving good contrast.

(3) Strong fatigue resistance in biological media; this directly determines the achievable resolution under the RESOLFT principle. An *n*-fold increase in resolution requires the dye to show only minor degradation after *n*^2^ switching cycles for 2D-imaging.

(4) High brightness and low toxicity.

(5) Use of visible light illumination to trigger switching. This is important because commercial microscopes typically do not have UV lasers and their optics have poor transmission at wavelengths shorter than 400 nm. Furthermore, UV light is damaging for cells and it has a short penetration depth through biological tissues.^18^

(6) The dye construct should be able to permeate cellular membranes, to allow intracellular staining in living cells.

There are at least two approaches to such a switchable dye: a single molecular switch having a brightly fluorescent form, and a comparatively dark state (Fig. 1b). Alternatively, a dyad can be used, in which the fluorescence of one unit is modulated by the other (Fig. 1c).^18^–^24^ The dyad approach has two main advantages: the requirements on the photochromic switch are less demanding because it does not need to be emissive, and the possibility of using different fluorescent dyes enables multi-color imaging.

Within the wealth of photochromic switches, we have chosen to focus on the spiroheterocyclic families^25^ (*i.e.* spiropyrans and spirooxazines). Spiropyrans are widely investigated, but they tend to show poor fatigue resistance. Spirooxazines, on the other hand, are less studied by the academic community, while being commonly used for industrial applications such as photo-responsive sunglasses and ophthalmic lenses.^26^ Our molecular design was inspired by numerous spironaphthoxazines reported in patents for various applications.^27^ In particular, spironaphthoxazines bearing an indolinyl substituent, known commercially as palatinate purple, typically show strong absorption for the closed isomer at 405 nm, whereas the closed forms of most spirooxazines do not absorb visible light.^28^,^29^

**Scheme 1:**
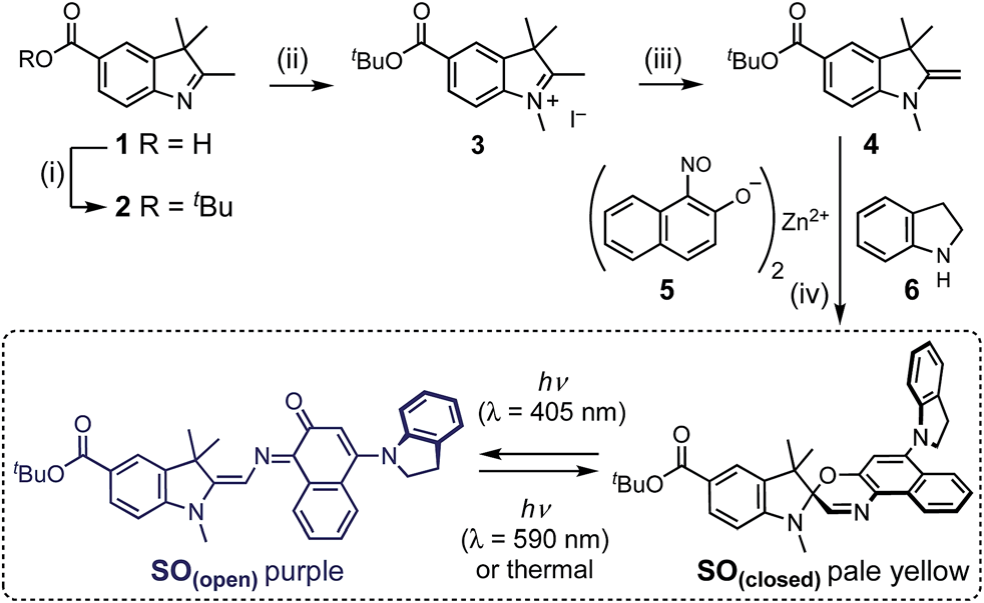
Synthesis of switch **SO**. (i) *tert*-Butyl 2,2,2-trichloroacetimidate, BF_3_OEt_2_, THF, 20 °C, 20 min, 64%. (ii) MeI, *o*-DCB, 80 °C, 24 h, 77%. (iii) KOH, 20 °C, 2 h, 99%. (iv) **5**, **6**, EtOH, air, reflux 2 h, then **4**, Na_2_SO_4_, reflux 21 h, 22%. The photo-switching mechanism of **SO** is shown.

For both spiropyran and spirooxazine families, reversible photochromism arises from interconversion between a closed form, that is colorless or slightly yellow, and an open form that is intensely colored. Upon photoactivation, the closed form undergoes ring opening *via* either heterolytic cleavage of the C_(spiro)_–O bond, or electrocyclization, to give the open form, which absorbs at a longer wavelength due to an extended π system (see the spironaphthoxazine **SO** in Scheme 1).^30^ Both families are T-type photoswitches, *i.e.* the forward reaction is driven by light and the reversion occurs spontaneously in the dark. In the case of spiropyrans and spirooxazines, the thermal back reaction can be photochemically accelerated.^31^ Despite similarities in the structures and switching mechanisms, spirooxazines exhibit significantly stronger fatigue resistance, which is a crucial factor for RESOLFT microscopy.

Here we report a spironaphthoxazine that fulfills the design guidelines necessary to be utilized as a tunable fluorescence quencher. We first describe its synthesis, crystal structure and basic photophysical characterization. The photochemistry and switching kinetics for both photo processes are elucidated next by the means of ultrafast time-resolved spectroscopies. Finally, we demonstrate that it can be used in combination with a commercial dye to create a dyad displaying outstanding fatigue resistance in living cells.

## Results and discussion

### Synthesis

The synthesis of molecular switch **SO** started with the preparation of indole **1**,^32^ which was converted to the *t*-butyl ester **2**, then methylated using methyl iodide and deprotonated by potassium hydroxide to yield Fischer's base **4**. The final spironaphthoxazine was prepared using chemistry developed by Pang, Meng and coworkers,^33^ by the one-pot condensation of the zinc-chelated nitrosonaphthol **5**, indoline **6** and Fischer's base **4**.

### X-ray crystallography and DFT calculations

**Fig. 2:**
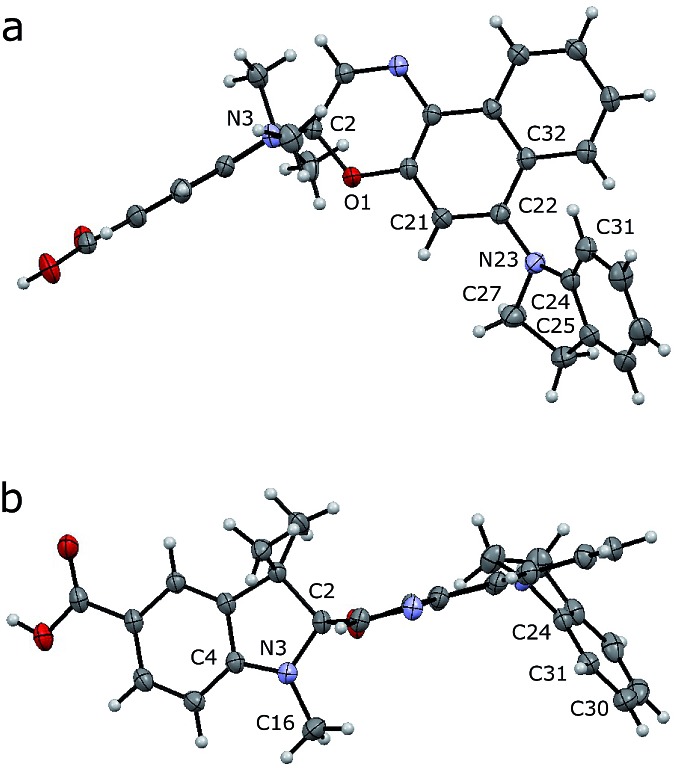
Crystal structure of **7** (deprotected **SO**). Side (a) and axial (b) views are shown with thermal ellipsoids at 50% probability.

Crystals of compound **7** (deprotected **SO_(closed)_**) suitable for X-ray analysis were obtained by slow evaporation of a solution in CH_2_Cl_2_/CH_3_OH at 25 °C. As in the structures of related spironaphthoxazines,^34^ the naphthoxazine and *t*-Bu-indole halves of the molecule are oriented orthogonally around the spiro sp^3^ carbon center (Fig. 2). This lack of π-conjugation implies that electronic transitions are mainly localized in each half of the molecule, giving rise to discrete absorption bands in the UV spectrum.^35^ Thus, the bathochromic shift into the visible region shown by this switch, compared to analogues with different substituents, is attributed to the indole-incorporating naphthoxazine component.^28^

In the naphthoxazine moiety, the indole nitrogen atom (N23) is slightly pyramidal; it is 0.28 Å above the plane of its three connected carbon atoms (C22/C24/C27). As expected, the indole unit is almost flat and the lone pair of this nitrogen interacts mainly with the indole π-system; the C22/C24/C27 and C25/C24/C31 planes are almost parallel (angle: 14.3°). There is a significant twist between the indole and naphthalene units; the angle between the C22/C24/C27 and C21/C22/C32 planes is 52.3°, indicating weak overlap between the indole nitrogen lone pair and the naphthalene π-system.

In the indole moiety, the N3 nitrogen atom is pyramidalized so as to align its lone pair anti-parallel to the C_(spiro)_–O bond; 
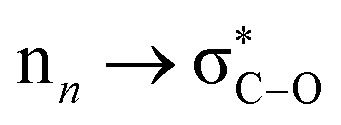
 donation appears to weaken this bond, reducing the barrier to ring-opening. The C_(spiro)_–O bond length (1.449 Å) in **7** is similar to that in other spirooxazines (mean = 1.46 Å, CCDC†), and slightly longer than a typical C–O single bond (about 1.43 Å). The extent of pyramidalization at N3 is similar to that at N23; N3 is displaced from the C2/C4/C16 plane by 0.22 Å.

We investigated **SO_(closed)_** by density functional theory (DFT) using previously optimized methods (B3LYP/6-31+G(d,p) with the polarizable continuum model for solvation by CH_2_Cl_2_).^36^,^37^ The DFT-optimized structure matches well with the crystal structure of **7**, indicating that the molecular geometry is not strongly influenced by crystal packing. The values of the parameters discussed above in this calculated structure are: displacement of N23 from C22/C24/C27 plane: 0.21 Å; angle between planes C22/C24/C27 and C25/C24/C31: 12.1°; angle between planes C22/C24/C27 and C21/C22/C32: 48.0°; displacement of N3 from C2/C4/C16 plane: 0.24 Å. TD-DFT calculations (cam-B3LYP/6-311+G(2d,p)) were used to predict the UV-vis absorption spectrum of **SO_(closed)_**, correctly reproducing the red-shifted absorption of indole-substituted spirooxazines: *λ*_max_(exp) = 388 nm *vs. λ*_max_(calc) = 365 nm. These calculations predict an energy difference of 4792 cm^–1^ between the relaxed singlet excited state and the singlet excited state generated by vertical excitation from the ground state (*i.e.* a shift from 365 nm to 442 nm), suggesting significant geometry changes. Comparison of the optimized geometries of the ground state and singlet excited state (see ESI, Fig. S19†) does not reveal a specific change leading to this energy difference but points toward minor changes across the whole molecule.

**Fig. 3:**
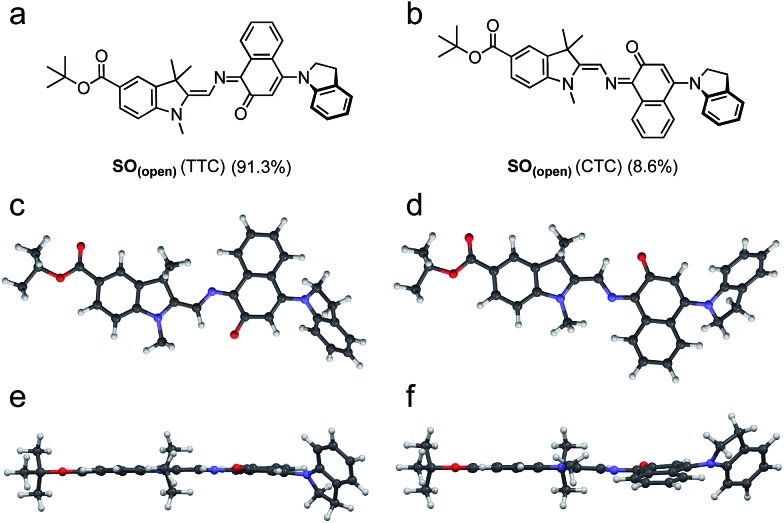
(a and b) Major isomers of **SO_(open)_** as predicted by DFT (B3LYP/6-31+G(d,p)). (c–e) Side (c and d) and top (e and f) views of the predicted structure of the major **SO_(open)_** isomers (TTC: (c) and (e); CTC (d) and (f)). Populations calculated for thermodynamic equilibrium.

DFT calculations were used to elucidate the structures of the open isomers of **SO**, which are not accessible by X-ray crystallography due to the fast thermal back reaction (<10 s). **SO_(open)_** can exist as several different stereoisomers. The most stable is TTC (*trans*–*trans*–*cis*, 91.3%) followed by CTC (*cis*–*trans*–*cis*, 8.6%) (Fig. 3), with the other isomers accounting for less that 0.1% of the total speciation at thermodynamic equilibrium, in agreement with results for similar systems.^36^

### Photochromic behavior

As described above, numerous criteria must be satisfied for a photochromic switch to be suitable for application in RESOLFT microscopy. Several of these properties can be tested directly in dilute solution at an early stage. The photophysical characteristics of our switch of choice, **SO**, are presented here.

#### UV-vis absorption and fluorescence

**Fig. 4:**
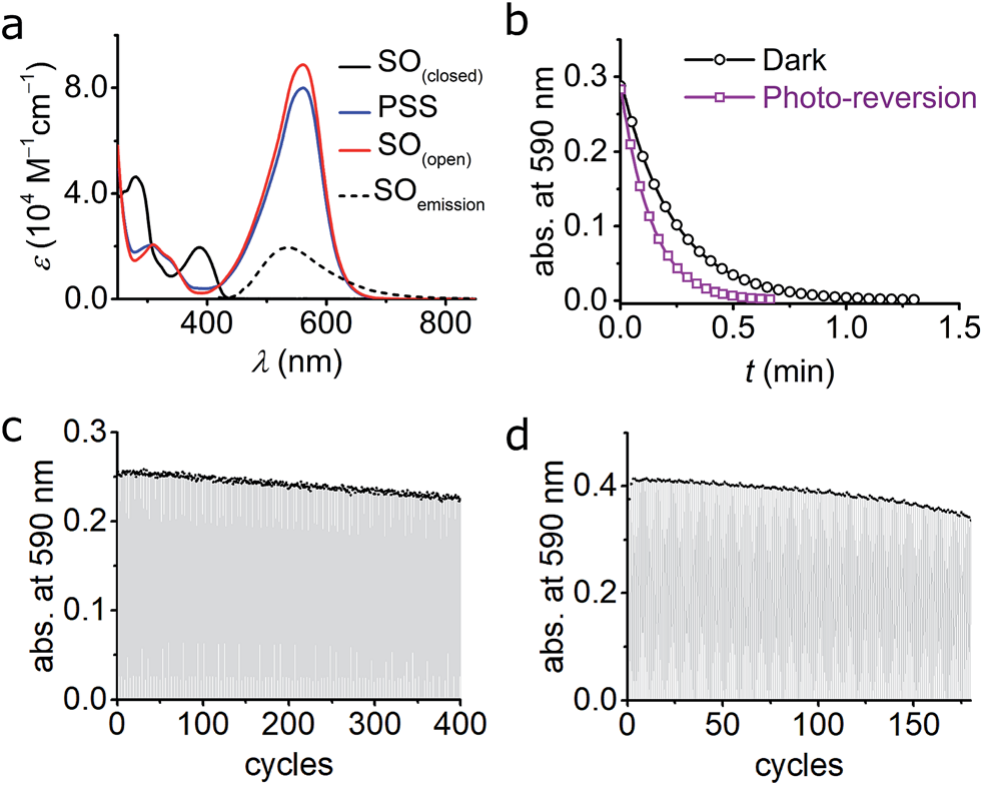
(a) UV-vis absorption of switch **SO_(closed)_**, **SO_(open)_**, and PSS in cyclohexane at 10 °C. The fluorescence spectrum of **SO_(closed)_** is included. The PSS was obtained by irradiation with blue (405 nm) light and the **SO_(open)_** was reconstructed mathematically (see ESI, Fig. S6†). (b) Kinetic traces for the ring-closure reaction **SO_(open)_** → **SO_(closed)_** after blue-light irradiation in cyclohexane. Both the thermal (black circles; fit, black line) and combined thermal and photochemical using green light (0.97 W cm^–2^, 100 ms irradiation, 400 ms interval, violet squares; fit, violet line) are shown. (c and d) Fatigue resistance of **SO** in cyclohexane (c) and CH_2_Cl_2_ (d) at 25 °C. Each cycle consists of blue irradiation (405 nm, 2.5 s, 2.1 W cm^–2^), and thermal relaxation.

The absorption spectrum of **SO_(closed)_** displays two main bands (281 nm, *ε* = 4.6 × 10^4^ M^–1^ cm^–1^ and 388 nm, *ε* = 2.0 × 10^4^ M^–1^ cm^–1^, Fig. 4a), as reported for similar spironaphthoxazines.^28^,^29^,^38^ Significant absorption is observed at 405 nm (*ε* = 1.5 × 10^4^ M^–1^ cm^–1^) indicating that it is possible to drive the photo-switching reaction using readily available 405 nm blue light. Compared to analogues with different substituents, spirooxazines incorporating an indole substituent display a bathochromic shift in the UV-vis absorbance.^28^,^38^ **SO** exhibits weak fluorescence (541 nm, *Φ*_f_ = 0.051 in CH_2_Cl_2_); similar fluorescence has been reported before for closely related spironaphthoxazines,^28^,^38^–^40^ whereas most spirooxazines show no detectable fluorescence.^41^ The large Stokes shift (153 nm, 7289 cm^–1^) suggests that significant relaxation takes place in the singlet excited state; as discussed above, our TD-DFT calculations predict a similar Stokes shift (4792 cm^–1^).

The photoisomerization reaction using 405 nm light was explored next. The open forms of spiro(naphth)oxazines are typically short-lived (less than 10 s),^28^–^30^,^38^,^39^ which makes them difficult to characterize. We measured the decay of **SO_(open)_** to estimate the rate of the thermal back reaction, which occurs in less than 100 ms in MeOH, while in apolar media lifetimes of a few seconds are observed (*e.g.* 4.7 s in CH_2_Cl_2_ at 25 °C see ESI, Section S6†). Accelerated thermal ring closure in polar solvents has been reported previously for spironaphthoxazines,^42^ although some spiropyrans display the opposite behavior.^43^ To explore the spectroscopic properties of **SO**, we used a custom-modified UV-vis spectrophotometer equipped with a pulsed LED light source (see ESI for details, Section S2†) to generate **SO_(open)_***in situ*. Under pulsed blue-light irradiation (405 nm, FWHM 20 nm, 80 mW, 100 ms pulse, 200 ms interval) **SO_(open)_** could be generated and observed in various solvents. The spectrum of the PSS under blue-light irradiation is presented in Fig. 4a. The spectrum of **SO_(open)_** can be calculated based on the observed PSS. **SO_(open)_** shows a very strong absorption band from 450 to 650 nm (*λ*_max_: 561 nm, *ε*: 8.9 × 10^4^ M^–1^ cm^–1^, Fig. 4a) which is in line with similar spironaphthoxazines.^28^,^29^,^44^

Remarkably, the **SO_(closed)_** band centered at 388 nm almost completely disappears in **SO_(open)_** implying that high degrees of conversion can be achieved using 405 nm light (85–95%). This is important for the reasons given above, requirement (5), *i.e.* a similar feature centered at *ca.* 350 nm would limit the use of the switch to custom-made microscopes.

The quantum yield of ring opening was measured by analyzing the changes in UV-vis absorption during excitation,^28^,^45^,^46^ but due to the rapid back-reaction it was necessary to use time-resolved absorption (down to 200 ms, see ESI, Section S9†). To minimize the interference of the back reaction, the photochemical quantum yield for ring opening (*Φ*_c→o_) was measured in cyclohexane at 10 °C giving a value of 8.0 ± 0.7%. In dichloromethane at 10 °C, a comparable value was measured (*Φ*_c→o_ = 7.7 ± 0.6%) implying that the solvent has little influence on the photochemistry of **SO**. These quantum yields are 2–4 times smaller than the values reported previously for related palatinate purple spironaphthoxazines.^28^,^38^,^46^

An important feature of spironaphthoxazines is that the thermal back reaction can be accelerated using light.^18^,^23^ We explored the photo-reversion reaction using the same set-up described above by starting with blue (405 nm) irradiation followed by several short pulses of green light (525 nm, FWHM 60 nm, 69 mW, 100 ms pulse, 400 ms interval). Fig. 4b shows the acceleration of the thermal back reaction under green illumination. Using similar methods as for the ring opening reaction (see ESI, Section S9†), we calculated the quantum yield of ring closing to be *Φ*_o→c_ = 1.1 ± 0.1%, in cyclohexane at 10 °C, in agreement with studies on related compounds.^28^

The changes in the absorption spectra, together with the quantum yields of ring opening and closing, result in a high PSS (*ca.* 85–95% open, as calculated from the absorption spectra)^47^–^49^ using 405 nm irradiation, which is a critical requirement for high contrast in microscopy applications.

#### Fatigue resistance

A crucial property of a molecular switch is its fatigue resistance (*i.e.* number of switching cycles the molecule can undergo before irreversible degradation), and this is most important in the context of RESOLFT, as mentioned above.

We investigated the fatigue resistance of **SO** in cyclohexane and CH_2_Cl_2_ by time-resolved absorption, alternating blue (405 nm) light irradiation and thermal relaxation (Fig. 4c and d). With less than 10% photo-degradation after 100 photo-cycles (in CH_2_Cl_2_), the fatigue resistance of **SO** is most promising compared to published dyes.^15^,^24^ We also investigated the fatigue resistance under photo-reversion conditions (*i.e.* by alternating blue and green irradiations during the measurements, see ESI, Section S11†), and confirmed that over 100 cycles can be achieved with less than 20% photo-degradation in cyclohexane. This result confirms that spirooxazines are remarkably fatigue-resistant switches.^25^,^30^

### Ultrafast photochemistry: time-resolved infrared (TRIR) and transient absorption (TA) spectroscopy

The experiments described above provide valuable insights into the most important properties of **SO**, such as a strongly colored open form, high fatigue resistance, and almost complete photo-isomerization using blue light. Further improvements to the molecular design require a thorough understanding of the underlying photochemistry. For instance, the photo-isomerization of spiropyrans can occur *via* either singlet or triplet pathways, or a combination of both,^46^,^50^ and the reactions of triplet excited states, for example with oxygen, can lead to photo-degradation.^51^

A number of photophysical studies have been reported on different spirooxazines,^44^,^50^–^54^ complemented by computational work.^36^,^37^ Photo-isomerization is thought to proceed *via* dissociation of the C_spiro_–O bond in the singlet electronic excited state, followed by the formation of a short-lived (*ca.* 10–100 ps) cisoid intermediate,^29^ sometimes identified as the CCC merocyanine (the so-called “X-intermediate”).^55^–^57^ This intermediate either relaxes back to the closed spirooxazine or transforms to one of the stable open merocyanine forms on a timescale of a few ps to ns. These isomers are thought to be in equilibrium, with the TTC and CTC forms predominating (Fig. 3).^29^ The kinetics of these processes and the distribution of the species are strongly dependent on the chemical structure.^25^

To date, the majority of studies of spirooxazines have been performed using transient absorption (TA) or time-resolved fluorescence,^58^ and often the results observed for spiropyrans are extrapolated to spiro(naphth)oxazines. To gather a more reliable understanding of the structure of the various intermediates and their kinetics, we turned to ultrafast time-resolved vibrational spectroscopy, namely time-resolved infrared, TRIR. Our DFT calculations (see ESI, Section S12†) suggest that the different open merocyanine isomers have significantly different IR signatures, while being virtually indistinguishable by UV-vis spectroscopy, and thus TRIR is an appealing tool to explore the isomerization process. While the relative stabilities of the **SO_(open)_** isomers are predicted to favor the TTC (91.3%) at thermodynamic equilibrium, whether the other isomers represent reaction intermediates is an open question that can be addressed using TRIR.

The LIFETime (TRIR in Time-Resolved Multiple Probe Spectroscopy mode, time resolution 1 ps up to 1 ms) and ULTRA (TA in Time-Resolved Multiple Probe Spectroscopy mode, time resolution 100 fs up to 10 μs) instruments at the STFC Rutherford Appleton Laboratory were used and are described in detail elsewhere.^59^ All samples were measured in a flow cell to prevent interference from **SO_(open)_** generated during photolysis. Experimental details are provided in the ESI (Section S13†).

**Fig. 5:**
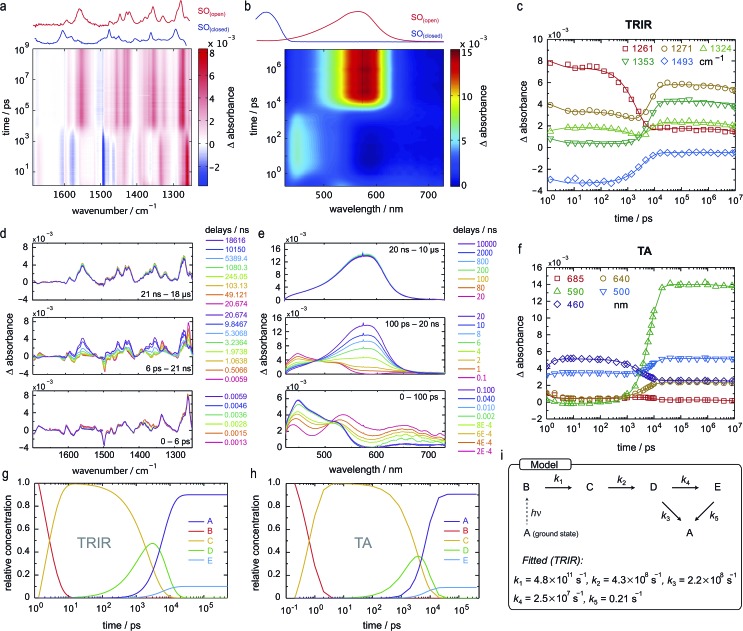
TRIR and TA spectra, in CD_2_Cl_2_ and CH_2_Cl_2_, respectively, and excited-state kinetics of the ring-opening process of **SO_(closed)_**. (a) TRIR contour spectrum; the steady-state spectra are shown for reference. (b) TA contour spectrum; the steady-state spectra are shown for reference. (c) TRIR kinetic traces for selected wavenumbers (scatter) and fitted kinetics (solid lines) using the values obtained from target analysis. (d) TRIR spectra for selected probe times. (e) TA spectra for selected probe times. (f) TA kinetic traces for selected wavelengths (scatter) and fitted kinetics (solid lines) using the values obtained from target analysis. (g and h) Speciation plot for the ring-opening reaction using the values obtained by target analysis for TRIR (g) and TA (h). (i) Model used to fit the TRIR and TA data. Laser details: TRIR: 405 nm pump, 800 nJ; TA: 400 nm pump, 200 nJ.

**Scheme 2:**
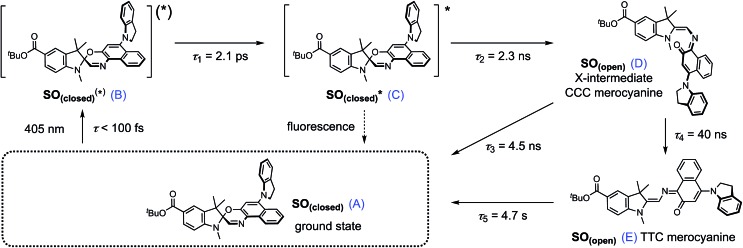
Proposed reaction mechanism for the ring opening process of **SO**. The indicated lifetimes correspond to the values determined by target analysis of the TRIR data in deuterated CH_2_Cl_2_; *τ*_5_ was determined separately. For simplicity, only the major isomer calculated by DFT is shown as photoproduct. SO_(closed)_^(^*^)^ corresponds to the non-relaxed singlet excited state. The structure of the reaction intermediate **SO_(open)_** X-intermediate (CCC-form) is shown for illustrative purposes and is based on previous literature.^29^

Both TRIR and TA spectra of **SO** in dichloromethane (CD_2_Cl_2_ for TRIR and CH_2_Cl_2_ for TA) show the presence of two clear transient species (Fig. 5a, b, d and e), indicated by distinct spectral features (*e.g.* in TA the absorption at 440 and 590 nm). Upon close inspection, a third intermediate can be identified and is necessary to correctly describe the system. Such an intermediate (D in Scheme 2) has been described previously for similar spirooxazines.^44^,^52^–^54^

In TRIR, the singlet excited state is formed within the first ps (*i.e.* the time resolution of the instrument), as indicated by the ground-state bleach 1493 cm^–1^, and decays with a lifetime of 2 ps to a second transient species (Fig. 5). The IR signatures of both transients are strikingly similar but distinct, suggesting only minor structural changes and opposing a putative vibrational cooling (Fig. 5d, lower panel). The generated singlet excited state displays a lifetime of *ca.* 3–9 ns (Fig. 5d, middle panel) and transforms either to the final **SO_(open)_** isomer or back to the **SO_(closed)_** ground state. The identity of **SO_(open)_** can be confirmed both by its long lifetime (>1 ms, Fig. 5d, upper panel) and comparison to the FT-IR spectrum of **SO_(open)_**, recorded under constant 405 nm illumination (Fig. 5a, top).

The TA spectra of **SO** could be easily related to the TRIR data, and both show similar kinetics. The first transformation (first 5 ps), characterized by a shift from *λ*_max_ = 534 to *λ*_max_ = 450 nm, is better observed in TA, suggesting a large change in electronic structure, relative to the ground state (Fig. 5e, lower panel). Excitation to the S_2_ state can be dismissed based on the excitation energy (*i.e.* too low for the predicted transition) and the calculated intensity of the transition (from DFT). Therefore, we assign this transition to a conformational relaxation of the singlet excited state. The large Stokes shift observed experimentally and predicted by TD-DFT supports this assignment. Next, a clear transformation to the **SO_(open)_** form is evidenced by the presence of well-defined isosbestic point at 478 nm. The identity of **SO_(open)_** is confirmed by its characteristic UV-vis spectrum (*λ*_max_ = 590 nm). Nevertheless, relatively poor fits to exponential decays (see ESI, Fig. S22 and S23†) and discrepancies regarding the accepted photo-conversion mechanism of spirooxazines^44^,^52^–^54^ implied that the data required a more detailed analysis.

Target analysis, *i.e.* globally fitting the data assuming a putative kinetic model, was applied to gain further insights into the photoreaction and its associated kinetics. The selected model (Fig. 5i) is based on the literature regarding the inclusion of an intermediate between the singlet excited state and the photoproduct. The data exclude significant formation of a triplet-excited state, based on the fact that we do not observe any transient species with lifetimes in the range 100 ns to 100 μs typical for triplets.

To reduce the number of fitted variables, certain assumptions were made: conformational relaxation was restricted to less than 3 ps, the thermal back reaction from **SO_(open)_** to **SO_(closed)_** was set to be longer than 1 ms,§
§Under our flow measuring conditions, the renewal of the solution happens within 1 ms, and it is this process that was observed instead of the thermal back reaction. and only solutions close to the experimental quantum yield for ring-opening were considered (*Φ*_o→c_ < 20%). Using these constraints, it is possible to fit both TRIR and TA data (Fig. 5c and f) to the model depicted in Fig. 5i and Scheme 2. The use of a simpler model excluding species C results in a poor fit to the TA data, and completely fails to describe the TRIR data. For simplicity, radiative and nonradiative relaxation of the singlet excited state (C → A) was not included in this model; this is expected to be <10% given that fluorescence occurs with a quantum yield of only 5.1%.

The overall model is as follows: photo-excitation happens during the pump pulse. Intermediate B corresponds to the singlet excited state, and C to the relaxed singlet excited state. Bond breaking leads to D, which is described in the literature as the “X intermediate”^55^–^57^ and is postulated to be the CCC-form of **SO_(open)_**.^29^ E corresponds to the final form **SO_(open)_** and A to the ground-state **SO_(closed)_**.

Based on this model, following conformational relaxation, the singlet excited state is transformed to the “X intermediate” (*τ* = 2.3 ns), which proceeds either back to the ground-state (*τ* = 4.5 ns) or to the final photo-product **SO_(open)_** (*τ* = 40 ns). The thermal back reaction occurs within seconds (*τ* = 4.7 s). The evolution of the different species is depicted in Fig. 5g and h. Our data do not show evidence of multiple, discernable, isomers of **SO_(open)_**, implying that if these isomers are intermediates, they must be short-lived or not resolvable.

The TRIR data enable us to estimate the quantum yield for ring-opening *Φ*_c→o_ using two different approaches: from the recovery of the ground-state bleach (eqn (1); *e.g.* at 1488 cm^–1^Fig. 5a), or from the rate constants deduced from target analysis (eqn (2)). Both methods give similar results (eqn (1): *Φ*_c→o_ = 7.4%; eqn (2): *Φ*_c→o_ = 10%, in CH_2_Cl_2_ at 20 °C) and match well with the value from steady state measurements (*Φ*_c→o_ = 7.7% in CH_2_Cl_2_ at 10 °C). Eqn (2) will over-estimate *Φ*_c→o_ because it ignores direct relaxation of the singlet excited state (C → A); if 10% of C relaxes directly to A, this would reduce *Φ*_c→o_ by 10%.1
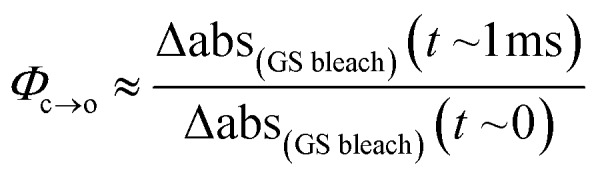

2
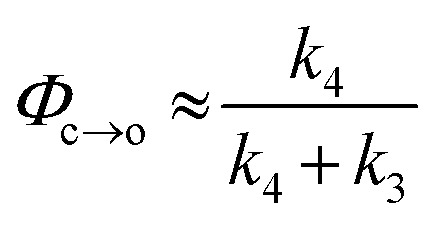



To further explore the ring-closing photo-reaction, a photo-stationary state was generated *in situ* within the time-resolved set-up using continuous irradiation with blue-light (405 nm). Since **SO_(closed)_** has no absorption above 500 nm, only the photo-generated species is excited by the pump pulse at 590 nm and any transients observed correspond to the ring-closing process. We opted to use TRIR to avoid visible light scattering problems that would be introduced in TA, and because it should be better able to discern the different **SO_(open)_** isomers.

**Fig. 6:**
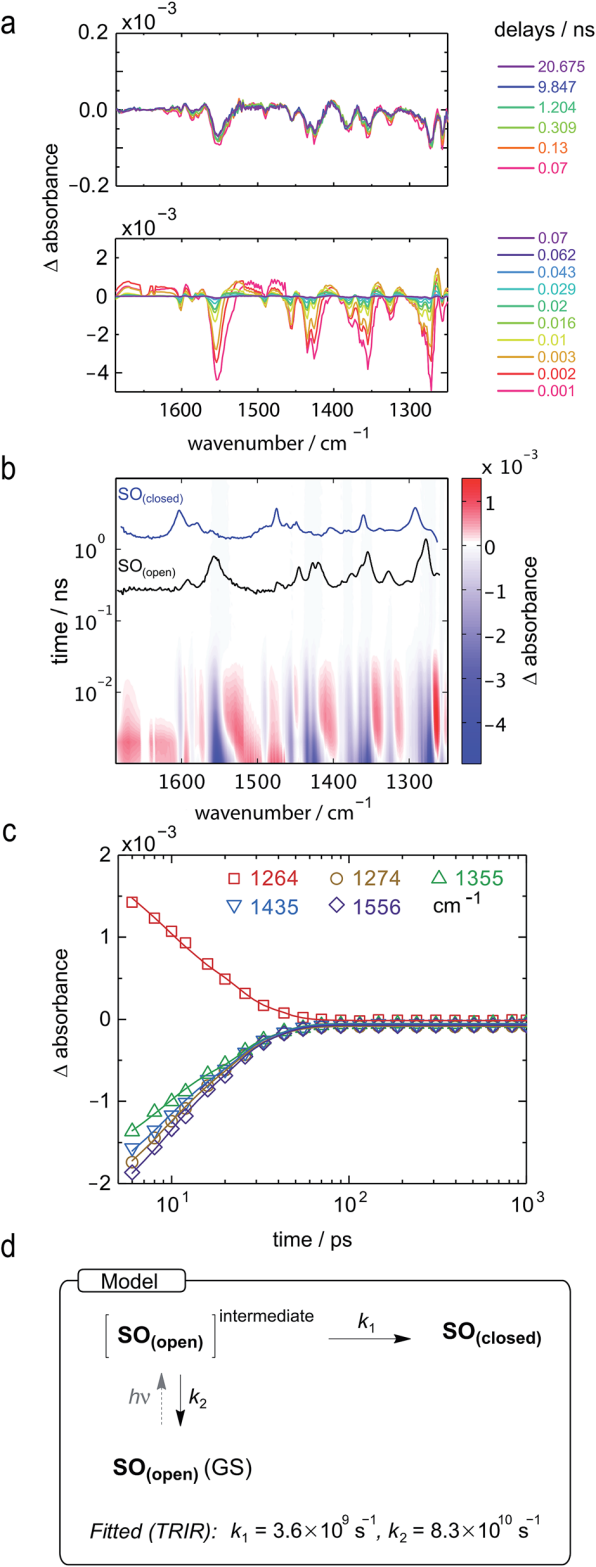
TRIR spectra and excited-state kinetics of the ring-closing process of **SO_(open)_** in CD_2_Cl_2_ by excitation at 590 nm. (a) TRIR spectra for selected probe times. (b) TRIR contour spectra; the steady-state spectra are shown for reference. (c) Kinetic traces for selected wavenumbers (points) and fitted kinetics (solid lines) using the values obtained from target analysis. (d) Model used to fit the TRIR. Laser energy: 800 nJ.

TRIR spectroscopy shows that photochemical ring-closure is about 1000 times faster than photochemical ring-opening (Fig. 6). Target analysis of the TRIR data indicates that the formation of the transient intermediate occurs almost instantaneously¶
¶An additional lifetime of 1–2 ps can be fitted using single wavenumber analysis, potentially indicating that the excited state and the intermediate are different species, but such a model did not yield satisfactory results using target analysis due to the proximity of time zero. after photo-excitation and decays back to **SO_(open)_** (*τ*_2_ = 12 ps) or is transformed into **SO_(closed)_** (*τ*_1_ = 280 ps). As before, the quantum yield of this photo-reaction can be estimated from the TRIR using two different approaches, eqn (3) and eqn (4). Again, the two methods give similar results (eqn (3): *Φ*_c→o_ = 1.4–2.0%; eqn (2): *Φ*_c→o_ = 4% in CD_2_Cl_2_ at 20 °C), which are comparable to the value from steady state measurements (*Φ*_c→o_ = 1.1% in cyclohexane at 10 °C).3
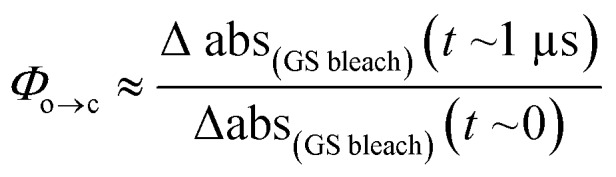

4
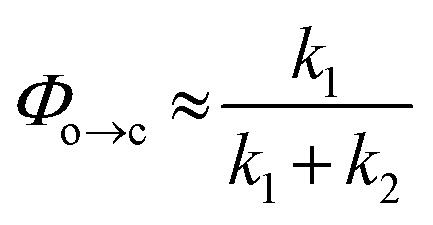



The ring-opening kinetics observed for **SO** are significantly different from those previously reported from studies of the ultrafast photochemistry of spirooxazines.^44^,^53^–^57^ For instance, in the absence of the ester substituent, the corresponding spirooxazine converts to its open form with *τ* = 1 ns, and was shown by time-resolved resonance Raman to proceed through multiple open-form intermediates.^29^ The substitution of the *N*-methyl by an isopropyl group resulted in a highly solvent-dependent photo-isomerization reaction.^44^ In general, the photoisomerization process occurs within a maximum timescale of a few ns. In our case, **SO** shows remarkably little solvent dependence (additional TA traces in acetone, DMSO, THF, and toluene can be found in the ESI, Fig. S24†) and the second step of photoisomerization is much slower (*τ* = 40 ns), than the previously reported cases. This difference may be attributed to the ester substitution on the indole moiety. The long-lived excited state of **SO_(closed)_** is also apparent from its steady-state properties: while most spirooxazines are non-emissive,^41^ it shows detectable fluorescence (*Φ*_f_ = 0.051).

### Model dyads and live-cell imaging

It is apparent from the results presented above that **SO** possesses many of the required photophysical properties to work as a fluorescence quencher in a putative FRET-based switchable fluorescent dyad. Nevertheless, other properties such as membrane permeability, photostability in cellular media, and quenching efficiency of the fluorescence of an attached dye (*via* FRET) can only be assessed correctly in real systems.

**Scheme 3:**
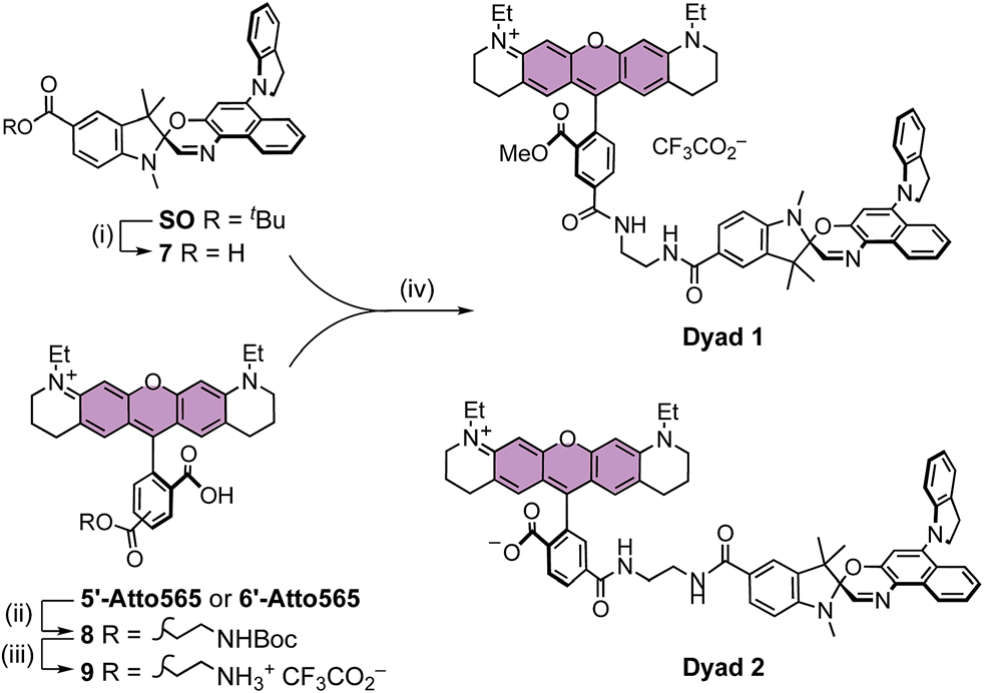
General scheme for the synthesis of **Dyad 1** and **Dyad 2**. (i) SiO_2_, toluene, reflux, 12 h, 77%. General protocol for both dyads: (ii) Boc-NHCH_2_CH_2_NH_2_, HBTU, TEA, DMF, 20 °C, 15 min. (iii) TFA, CH_2_Cl_2_, 20 °C, 1 h. (iv) HBTU, TEA, DMF, 20 °C, 1 h.

To investigate the feasibility of a switchable dye, we decided to prepare two model dyads: **Dyad 1** and **Dyad 2**. Both dyads are composed by a well-known fluorescent dye, Atto565,^60^ covalently linked to **SO** (Scheme 3). Assuming a FRET mechanism of energy transfer and a donor–acceptor distance of 15.4 Å (the calculated center-to-center distance in the unfolded conformation), our calculations based on the spectral overlap suggest that a quenching efficiency above 99% is expected from such a dyad system (see ESI, Section S10†). Moreover, given the broad absorption of **SO_(open)_**, various other dyes (*e.g.* AlexaFluor488®) should be quenched with similar efficiencies, which is important for multi-color imaging.

#### Synthesis

The synthesis of both dyads follows a similar sequence (Scheme 3). Fluorescent dye **5′-Atto565** was coupled to Boc-protected ethylenediamine using HBTU as coupling agent, giving compound **8**, which was converted to **9** by TFA promoted Boc-deprotection. In parallel, the *t*Bu-protecting group of **SO** is removed under mild acidic conditions using silica-gel in refluxing toluene^61^ to give **7**, which was coupled to **9** and MeOH to afford dyad **Dyad 1**. A similar procedure was used to prepare **Dyad 2** from the 6′-isomer of Atto565 without protecting the acid at the 3′-position. The main difference between the dyads is the permanent cationic form of **Dyad 1**, while **Dyad 2** can exist as a neutral zwitterion.

#### Photo-physical characterization

**Fig. 7:**
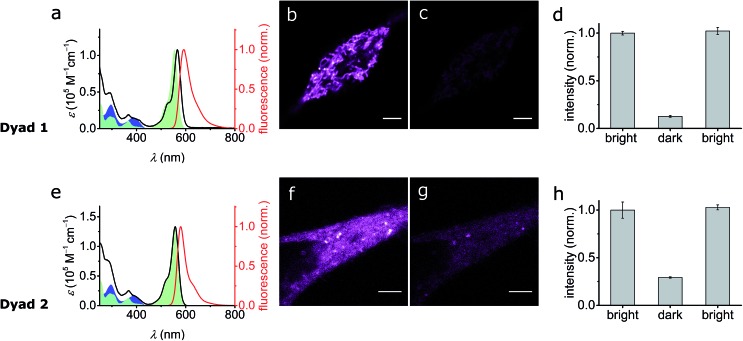
(a and e) UV-vis absorption and emission spectra for **Dyad 1** (a) and **Dyad 2** (e) in MeOH. The absorption of **SO_(closed)_** and **5′-Atto565** are shown in blue and green respectively for reference. (b and f) Confocal image in live HEK cells stained with **Dyad 1** (b) and **Dyad 2** (f) in the bright state by excitation at 561 nm. (c and g) Confocal image of **Dyad 1** (c) and **Dyad 2** (g) in the dark state by irradiation at 405 nm immediately followed by excitation at 561 nm. (d and h) Quantification of the quenching achieved with **Dyad 1** (d) and **Dyad 2** (h) in panels b, c and f, g. Scale bar: 5 μm.

Both dyads were investigated for their basic photophysical properties. In methanol, the absorption spectra of the dyads are close to the sum of those of their components (Fig. 7a and e). The observed absorption of **SO_(open)_** overlaps nicely with the emission of Atto565 and the fluorescence lifetimes are identical within the error of the measurement (*e.g.***5′-Atto565** and **Dyad 1** have fluorescence lifetimes of *τ* = 4.05 ns and *τ* = 3.88 ns respectively; see Table S1†). Curiously, the fluorescence quantum yields of the dyads show a marked decrease in aqueous phosphate-buffered saline (*e.g.* for **Dyad 1***Φ*_f_ = 33% in MeOH and *Φ*_f_ = 3.6% in PBS) which, taken together with the virtually unchanged fluorescence lifetime, suggests a static quenching mechanism. This decrease in fluorescent quantum yield is attributed to aggregation because it is accompanied by marked changes in the absorption spectra (see ESI, Fig. S3†). The fact that *Φ*_f_ of the dyads remains lower than that of Atto565 even in solvents such as CH_2_Cl_2_ (40% against 90%) indicates that other quenching mechanisms are involved, but remain minor with respect to aggregation.

#### Living cell imaging

The most important question is whether spironaphthoxazines can function in biological media. Indeed, there are no examples of this family of photochromic switches being used in cells and very few examples in aqueous media, typically in vesicles^62^ or nanoparticles,^63^ have been reported.

To investigate the switching and fluorescence quenching of dyads **Dyad 1** and **Dyad 2** in biological media, we added solutions of the dyads in DMSO directly to cell culture media of live human embryonic kidney cells (HEK 293 cells from ATCC, Manassas, VA, USA). After 15 min incubation, we removed the excess dye and recorded confocal images of the stained cells. Cellular uptake of the dye is confirmed for both dyads by the observation of fluorescence from inside the cell (excitation wavelength 561 nm, 5 μW, monitoring the fluorescence at 570–650 nm). In spite of the similarity of the dyads, their localization within the cell is significantly different: while **Dyad 1** accumulates in the intracellular compartments, **Dyad 2** appears to be dispersed in the cytosol. This is important because it suggests that cellular uptake and intracellular localization can be controlled by the choice of dye, rather than being dominated by the hydrophobicity of **SO**.

**Fig. 8:**
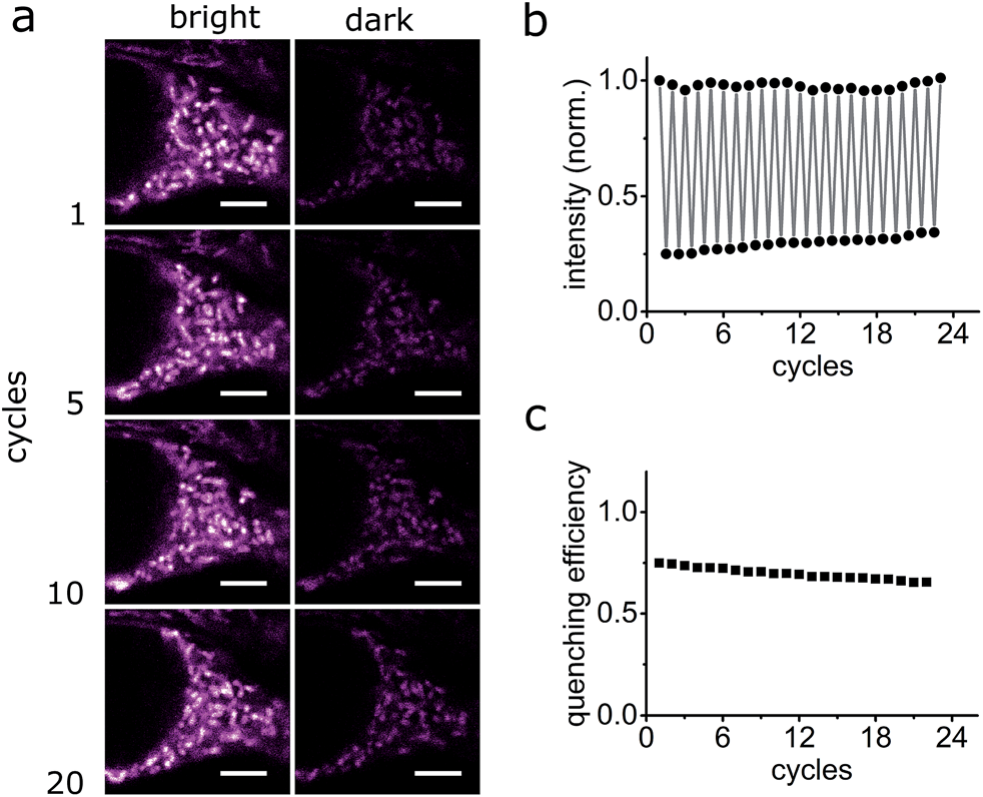
Fatigue resistance of **Dyad 1** in living HEK cells. (a) Confocal images of **Dyad 1** in living HEK cells in the bright and dark states at various switching cycles. (b and c) Fluorescence quantification of **Dyad 1** over several switching cycles. Quenching efficiency refers to the effective quenching of florescence per cycle. Scale bar: 5 μm.

The photo-switching of the spironaphthoxazine, and the concomitant fluorescence quenching using **Dyads 1** and **2**, was explored in the cells using a commercial confocal microscope. The field of view was scanned first with a green laser (561 nm, 5 μW), to acquire the “bright” image (Fig. 7b and f), and then sequentially, line-by-line, with a blue laser (405 nm, 30 μW) followed immediately by the image acquisition using the green laser (Fig. 7c and g) to obtain the “dark” image. Following this procedure, quenching efficiency values of 85–88% for **Dyad 1** and 70–80% for **Dyad 2** could be consistently recorded (Fig. 7d and h). Under the same conditions, the fatigue resistance in cellular media was measured by repeating the photo-switching sequence described above. The fatigue resistance of both dyads was measured and a typical example is presented in Fig. 8. **Dyad 1** loses 15% of its quenching efficiency after 23 cycles (Fig. 8b and c). It is important to notice that while the pixel size is set to 80 nm, the actual illumination spot size is larger (*ca.* 320 nm because of the diffraction-limited illumination) implying that every switching cycle effectively involves 4 switching events. Assuming that a minimum contrast of 50% is necessary to discern both states, an increase in resolution of 10 times is to be expected using a RESOLFT set-up.^15^ A similar fatigue resistance is measured for **Dyad 2** (see ESI, Fig. S32 and S33†).

The FRET-based dyad systems showed high fatigue resistance, notably also in the live-cell environment.^24^ We expect that both the contrast and the fatigue resistance can be further improved by optimizing the imaging parameters for a pixel-by-pixel imaging mode. Moreover, it has been shown previously that it is possible to obtain super-resolved images by applying the RESOLFT principle using dyes with poorer contrast^9^ or fatigue resistance.^15^

## Conclusions

We have described the synthesis and characterization of a spironaphthoxazine switch as a potential photochromic FRET-quencher for RESOLFT microscopy. The photochromic switch displays a strong absorbance at 405 nm, which is almost completely absent in the active, colored form. Due to the favorable quantum yield of ring-opening, and a comparatively poor quantum yield of ring closing, an uncommonly high photoconversion can be achieved using conventional equipment. The possibility to achieve high photochromic conversions using readily accessible non-UV light sources is of great interest in microscopy.

The ultrafast photochemistry of this spironaphthoxazine molecular switch is shown to be simpler than other reported spiro-photoswitches and no evidence of a triplet-excited state was observed. Moreover, the kinetic traces show no evidence of multiple merocyanine isomers. The results indicate that **SO** has clean photochemistry and good fatigue resistance.

Most importantly, we have not only demonstrated that spirooxazines can be used in living cells, but that it is possible to use them to modulate the fluorescence of a covalently attached fluorescent dye in biological media. The cell studies substantiate the excellent fatigue resistance within the working RESOLFT environment and indicate that the performance of these dyads in living cells is extremely promising. The intracellular localization differences observed between **Dyad 1** and **Dyad 2** also indicate that spironaphthoxazine may be tailored to target specific cellular domains. We are currently exploring the use of fusion proteins to demonstrate the feasibility of RESOLFT microscopy using versions of these **SO** dyads.

## Conflicts of interest

There are no conflicts to declare.

## Supplementary Material

Supplementary information

Crystal structure data
