# Correction: Spironaphthoxazine switchable dyes for biological imaging

**DOI:** 10.1039/c8sc90071j

**Published:** 2018-03-29

**Authors:** Yaoyao Xiong, Andreas Vargas Jentzsch, Johannes W. M. Osterrieth, Erdinc Sezgin, Igor V. Sazanovich, Katharina Reglinski, Silvia Galiani, Anthony W. Parker, Christian Eggeling, Harry L. Anderson

**Affiliations:** a Department of Chemistry , University of Oxford , Chemistry Research Laboratory , Oxford OX1 3TA , UK . Email: harry.anderson@chem.ox.ac.uk; b MRC Human Immunology Unit , Weatherall Institute of Molecular Medicine , University of Oxford , OX3 9DS , Oxford , UK; c Central Laser Facility , Research Complex at Harwell , Science and Technology Facilities Council , Harwell Campus , Didcot OX11 0QX , UK; d Institute of Applied Optics , Friedrich-Schiller-University Jena , Jena , Germany; e Leibniz Institute of Photonic Technology e.V. , Jena , Germany

## Abstract

Correction for ‘Spironaphthoxazine switchable dyes for biological imaging’ by Yaoyao Xiong *et al.*, *Chem. Sci.*, 2018, **9**, 3029–3040.



## 

Andreas Vargas Jentzsch should have been included in the footnote reference ‡, as the first two authors contributed equally, and the corrected author listing is shown here.

The Royal Society of Chemistry apologises for these errors and any consequent inconvenience to authors and readers.

