# Supplementary material for: Spironaphthoxazine switchable dyes for biological imaging
Source: Chem Sci. 2018 Feb 20;9(11):3029–40. doi: 10.1039/c8sc00130h (PMC5916019; doi:10.1039/c8sc00130h)
Supplement: Supplementary file 1 [file SC-009-C8SC00130H-s001.pdf]

Electronic Supplementary Material (ESI) for Chemical Science.
This journal is © The Royal Society of Chemistry 2018

Supplementary Electronic Information

Spironaphthoxazine switchable dyes for biological imaging

Yaoyao Xiong,[a,†] Andreas Vargas Jentzsch,[a,†] Johannes W. M. Osterrieth,[a] Erdinc Sezgin,[b] Igor V. Sazanovich,[c] Katharina Reglinski,[b] Silvia Galiani,[b] Anthony W. Parker,[c] Christian Eggeling,[b,d] Harry L. Anderson[a,*]

[a] Oxford University, Department of Chemistry, Chemistry Research Laboratory, Oxford OX1 3TA, UK. [b] Oxford University, MRC Human Immunology Unit and Wolfson Imaging Centre Oxford, Weatherall Institute of Molecular Medicine, Oxford OX3 9DS, UK. [c] Central Laser Facility, Science and Technology Facilities Council, Rutherford Appleton Laboratory, Harwell Science and Innovation Campus, Didcot, Oxfordshire OX11 0QX, UK. [d] Institute of Applied Optics Friedrich-Schiller-University Jena, Jena, Germany and Leibniz Institute of Photonic Technology e.V., Jena, Germany.

[†]Y. X. and A.V.J. contributed equally.

Table of Contents

Section S1	Synthesis of spironaphthoxazine switches and dyads	S2
Section S2	General procedures	S3
Section S3	Photophysical properties	S3
Section S4	Solvatochromism	S6
Section S5	Acquisition of the photostationary state (PSS) spectra	S7
Section S6	Rates of thermal ring closure	S8
Section S7	Fluorescence lifetimes	S8
Section S8	Re-absorption corrected fluorescence absolute quantum yields	S10
Section S9	Quantum yields of photochemical reactions	S11
Section S10	FRET efficiency (E) calculations	S12
Section S11	Fatigue resistance in the cuvette	S13
Section S12	DFT calculations	S14
Section S13	Ultrafast spectroscopy	S19
Section S14	Crystal structure	S23
Section S15	Cell culture and staining procedure	S25
Section S16	Optical microscopy	S25
Section S17	Experimental synthetic procedures	S28
Section S18	References	S33
Section S19	Supporting spectra	S34—S49

S1. Synthesis of spironaphthoxazine switches and dyads

Compound **7** was prepared in two routes: deprotection of switch **SO** and one-pot reaction of compound **S1** with zinc-chelated nitrosonaphthol (Scheme S1). The latter approach afforded product **7** with a minor amount of impurities in 28% yield, but the purity is sufficient for the subsequent coupling reaction. Switch **SO** was isolated in high purity, and used in the measurements of time-resolved IR and transient absorption.

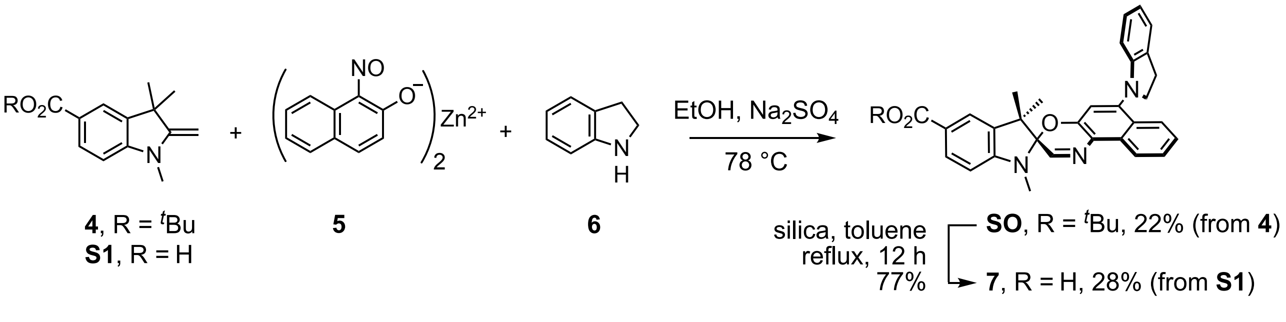

Scheme S1. Synthesis of spironaphthoxazines using a zinc chelate complex.[1]

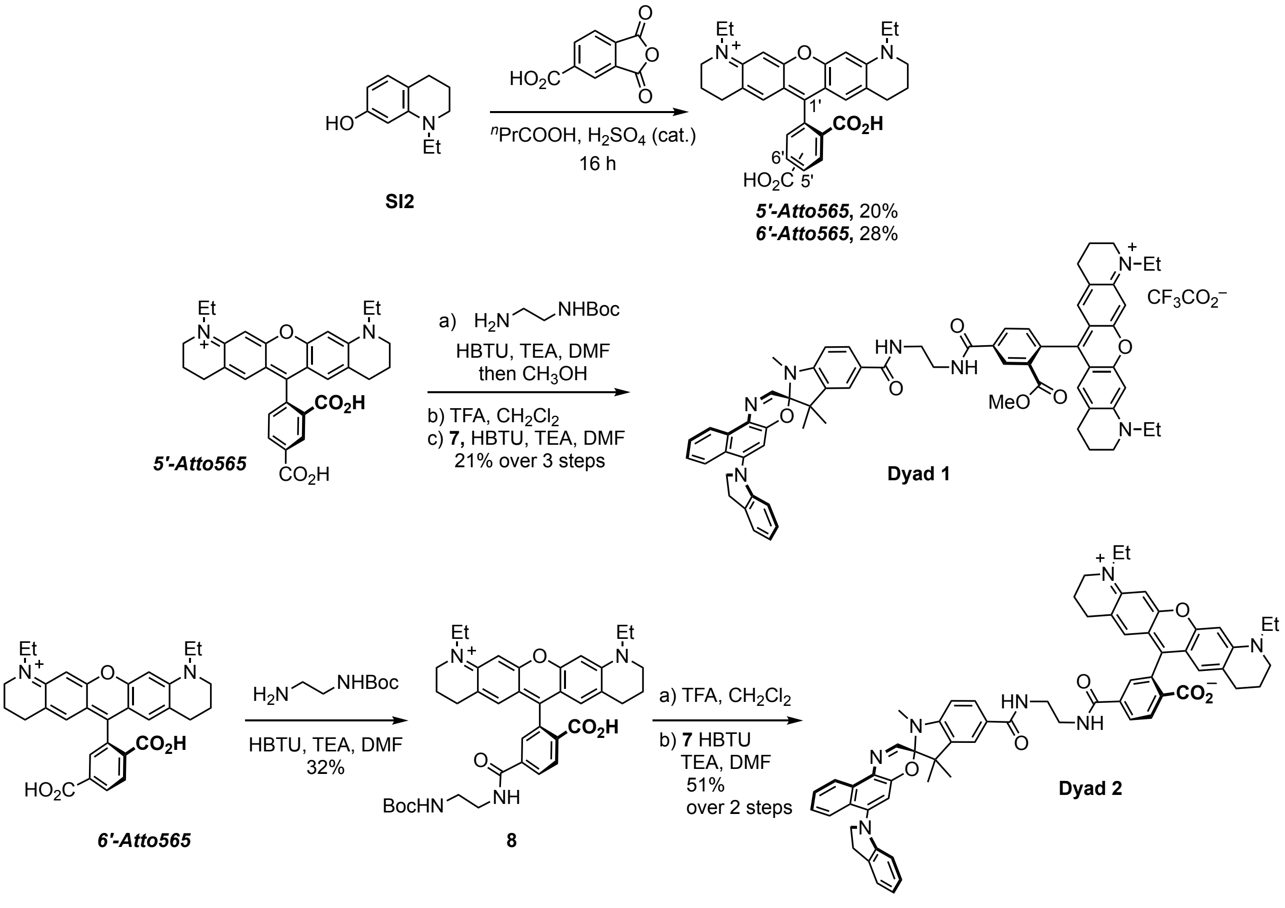

Scheme S2. Synthesis of **Atto565** dyes based on a modified procedure,[2] and synthesis of **Dyads 1** and **2**.

S2. General procedures

All reagents were purchased from commercial sources and used as received. Solvents were procured from Honeywell, formerly Sigma Aldrich. DMF was distilled and stored over molecular sieves under argon. Column chromatography was carried out using SiO_2 60 (particle size 40–63 μm, Merck, UK) as stationary phase. NMR spectra were acquired on a Bruker AVII400, AVIII400, or AVII500 instrument. ^1H NMR chemical shifts are reported in ppm and were referenced internally to residual protons in the solvent (δ = 7.26 for $CDCl_3$; 3.31 for CD_3OD). $^{13}C\{^1H\}$ NMR chemical shifts are reported in ppm and were referenced internally with respect to solvent signal (δ = 77.2 for $CDCl_3$; 49.0 for CD_3OD). Standard abbreviations indicating multiplicity were used as follows: s = singlet, d = doublet, dd = double of doublets, t = triplet, q = quartet, m = multiplet, br. = broad signal. High-resolution mass spectra (HRMS) were obtained on a Bruker μTOF instrument or a Waters GCT.

Stock solutions of all compounds were prepared at concentrations in the range 0.1–5 mM and stored at −20 °C, and thawed immediately before each experiment. Spectroscopic measurements were conducted in HPLC grade solvents. The irradiation sources were mic-LED-405 (from Prizmatix Ltd.; centered at 405 nm, FWHM = 15 nm) up to 360 mW, and mic-LED-525 (from Prizmatix Ltd; centered at 525 nm, FWHM = 60 nm) up to 69 mW. To measure the ring-opening and -closing processes, the two LEDs were joined into one output beam *via* a beam combiner, which was coupled to a liquid light guide (diameter = 3 mm). The liquid light guide was wired close to the cell holder and orthogonal to the beamline of the spectrometer. The irradiation was performed using Matlab programs to control light sources, intensities and interval lengths, and the number of cycles. The LED power was measured as previously described.[4] The UV-vis absorption spectra were obtained on a Perkin Elmer Lambda 20 spectrometer using quartz cuvettes from Starna (10 mm path length), and temperature was controlled by a PTP-1 peltier unit from Perkin Elmer. For characterization of photo-physical and -chemical properties, the sample concentration was adjusted to have absorbance at the excitation wavelength below 0.1 to avoid the inner filter effect. The emission spectra were obtained on a FS5 fluorescence spectrophotometer (Edinburgh Instruments). Where necessary, Matlab and OriginLab softwares were used for data treatment.

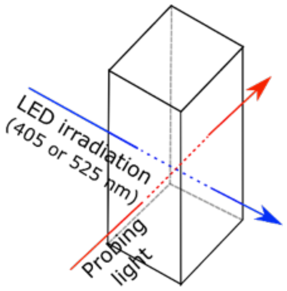

Figure S1. Schematic representation of the geometrical arrangement of the sample holder in the UV-vis spectrometer, for measurements of the PSS spectrum, fatigue resistance, and photochemical ring closure.

S3. Photophysical properties

The UV-vis absorption spectrum of **SO** in CH_2Cl_2 was compared with the excitation spectrum of the same sample with detection at 560 nm. The emission spectrum was recorded with excitation at 405 nm, and fluorescence emission was measured between 420 and 800 nm.

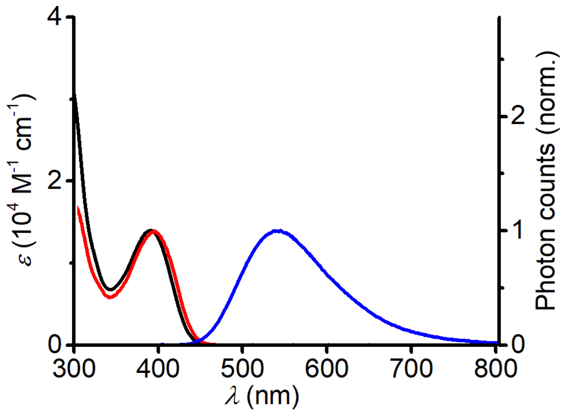

Figure S2. Absorption (black), excitation (red) and emission spectra (blue) of **SO** in CH_2Cl_2. The measurements were taken of a 10 µM solution at 25 °C. The excitation spectrum was monitored at 560 nm, and the emission spectrum was recorded once excited at 405 nm.

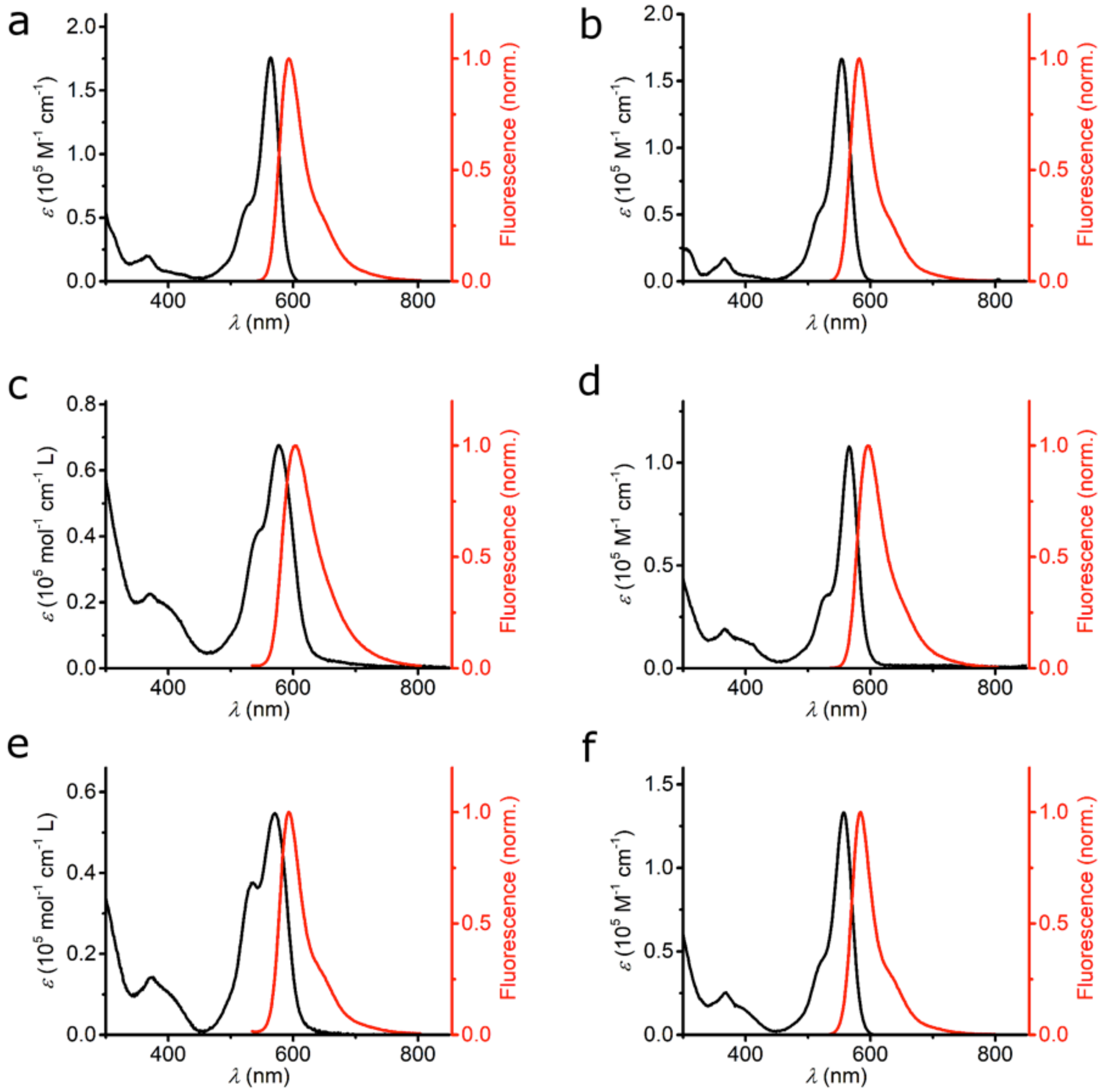

Figure S3. Absorption and normalized emission spectra at µM concentrations of a) **5'-Atto565** in PBS. b) **5'-Atto565** in CH_3OH. c) **Dyad 1** in PBS. d) **Dyad 1** in CH_3OH. e) **Dyad 2** in PBS. f) **Dyad 2** in CH_3OH.

Table S1. Photophysical properties of selected compounds in various solvents.

Compound (solvent)	$\lambda_{abs,\ max}$ (nm) ε (M^{-1} cm^{-1})	$\varepsilon_{570}/\varepsilon_{405}$	$\lambda_{em,\ max}$ (nm)	Fluorescence lifetime τ (ns), fitness χ^2	$\Phi_{fluorescence}$ (%)
**SO (CH_2Cl_2)**	**390**	0	533	–	5.1%
**5'-Atto565 (PBS)**	**564** 1.8 × 10^5	22.5	589	τ = 4.05, χ^2 = 0.98	79%; 80%[a]
**5'-Atto565 (CH_3OH)**	**554** 1.7 × 10^5	19.6	578	τ = 4.16, χ^2 = 1.02	85%; 85%[a]
**Mix of 5'- and 6'-Atto565 isomers[3] (PBS)**	**564**	–	590	4.0	90%
**Dyad 1 (PBS)**	**576** 6.8 × 10^4	3.6	598	τ = 3.88, χ^2 = 1.39	3.6%
**Dyad 1 (CH_3OH)**	**566** 1.1 × 10^5	8.4	591	τ_1 = 1.68, τ_2 = 3.70, χ^2 = 1.09	33%; 33%[a]
**Dyad 1 (CH_2Cl_2)**	**567** 1.2 × 10^5	10.5	587	τ_1 = 1.19, τ_2 = 3.18, χ^2 = 1.18	47%; 48%[a]
**Dyad 1 (DMSO)**	**578** 1.0 × 10^5	9.6	605	τ_1 = 0.24, τ_2 = 3.39, χ^2 = 1.31	65%; 66%[a]
**Dyad 2 (PBS)**	**571** 5.5 × 10^4	5.5	589	τ = 4.00, χ^2 = 1.78	4.3%
**Dyad 2 (CH_3OH)**	**557** 1.3 × 10^5	6.3	580	τ_1 = 1.43, τ_2 = 3.55, χ^2 = 1.04	42%; 43%[a]

[a]The values are re-absorption corrected.

S4. Solvatochromism of SO

In each solvent, the sample was prepared to have the maximum absorbance between 0.04 and 0.07, and the UV-vis absorption spectrum was recorded. For emission spectra, the same sample was excited at 405 nm, and fluorescence emission in the region 420 – 850 nm was measured.

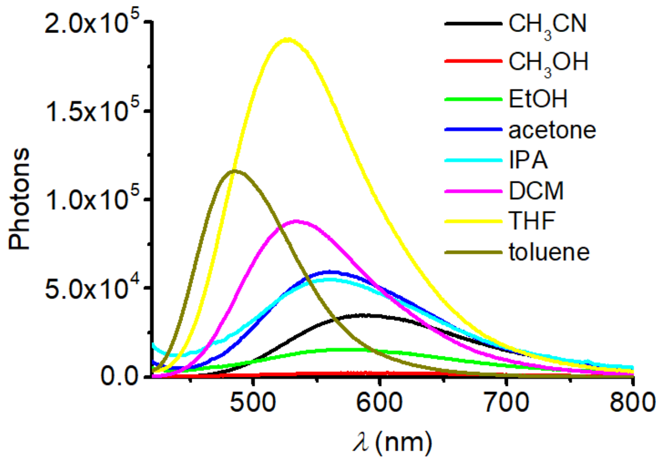

Figure S4. Emission spectra of **SO** in different solvents. The measurements were taken of a 3.3 μM solution at 25 °C once excited at 405 nm.

Table S2. Solvatochromism property of **SO** in different solvents.

**Solvent**	**$\lambda_{abs, max}$ (nm)** ε (M^{-1} cm^{-1})	**$\varepsilon_{abs, 405nm}$ (M^{-1} cm^{-1})**	**$\lambda_{em, max}$ (nm)**
**CH_3CN**	**387** 1.3×10^4	9.8×10^3	592
**CH_3OH**	**389** 1.2×10^4	9.3×10^3	574[a]
**EtOH**	**389** 1.5×10^4	1.2×10^4	570
**acetone**	**389** 1.3×10^4	1.0×10^4	560
**2-propanol**	**388** 1.5×10^4	1.2×10^4	562
**CH_2Cl_2**	**390** 1.4×10^4	1.2×10^4	533
**THF**	**389** 1.5×10^4	1.2×10^4	528
**toluene**	**391** 1.4×10^4	1.2×10^4	485

[a]The emission is very weak.

While the absorption spectra remain unchanged, significant bathochromic shifts of the emission wavelength are observed with increasing solvent polarity, with exceptions of solvents of hydrogen bonding capacity, *e.g.* 2-propanol. This is because **SO**, as a polar compound, has a larger dipole moment at the excited state relative to the ground state, and the interaction with polar solvents lowers the energy of the excited state, thus resulting in a red shifted emission wavelength. In addition to solvent polarity, other interactions between compounds and solvents, *e.g.* hydrogen bonding, can also give rise to spectral shifts.

S5. Acquisition of the photostationary state (PSS) spectra

A typical experiment is as follows: a solution of **SO** in cyclohexane (10 μM, 2.0 mL) at 10 °C was irradiated with pulses of 405 nm LED (2.1 W/cm^2, 100 ms irradiation, 200 ms interval), during which the PSS spectrum was recorded by the UV-vis spectrometer. The raw spectrum was processed in Matlab to remove the negative spikes due to scattering of the excitation light. In addition, conversion from the closed to open form was estimated to be about 85–95% to give a smooth **SO$_{(open)}$** spectrum. The **SO$_{(open)}$** spectrum was used to calculate the FRET efficiency.

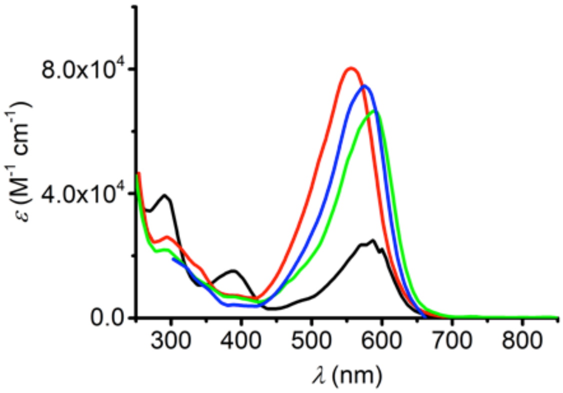

Figure S5. The PSS spectra of **SO** in different solvents, *i.e.* CH_3CN (black), cyclohexane (red), CH_2Cl_2 (blue), toluene (green) after irradiation at 405 nm.

Estimation of the PSS composition and SO$_{(open)}$ spectrum

Given the quick kinetics of ring closing, standard methods (e.g. NMR or HPLC analysis) are not suitable for estimating the composition of the PSS and, therefore, it is not possible to directly calculate the spectrum of **SO$_{(open)}$**. Three approaches could be considered:

(a) Assume that the PSS is 100% **SO$_{(open)}$** and 0% **SO$_{(closed)}$**. In this case the absorption spectrum of the PSS would be the same as that of **SO$_{(open)}$**.

(b) Assume that **SO$_{(open)}$** has no absorption at 390 nm. In this case the composition of the PSS can be easily calculated as 20% **SO$_{(closed)}$** because the ratio of the absorption of the PSS at 390 nm divided by the initial absorption of **SO$_{(closed)}$** at 390 nm is 0.2.

(c) If the fraction of **SO$_{(closed)}$** in the PSS is x, where x is in the range 0–20%, then the absorption spectrum of **SO$_{(open)}$** can be calculated by subtraction a fraction x of the absorption spectrum of **SO$_{(closed)}$** from the absorption of the PSS (Figure S6). Assuming that the absorption spectrum of **SO$_{(open)}$** is a smooth curve, and that it does not have zero absorption at 390 nm, implies that the composition of the PSS is 85–95% **SO$_{(open)}$**. The assumption of a smooth spectrum is supported by the results of the TD-DFT calculations.

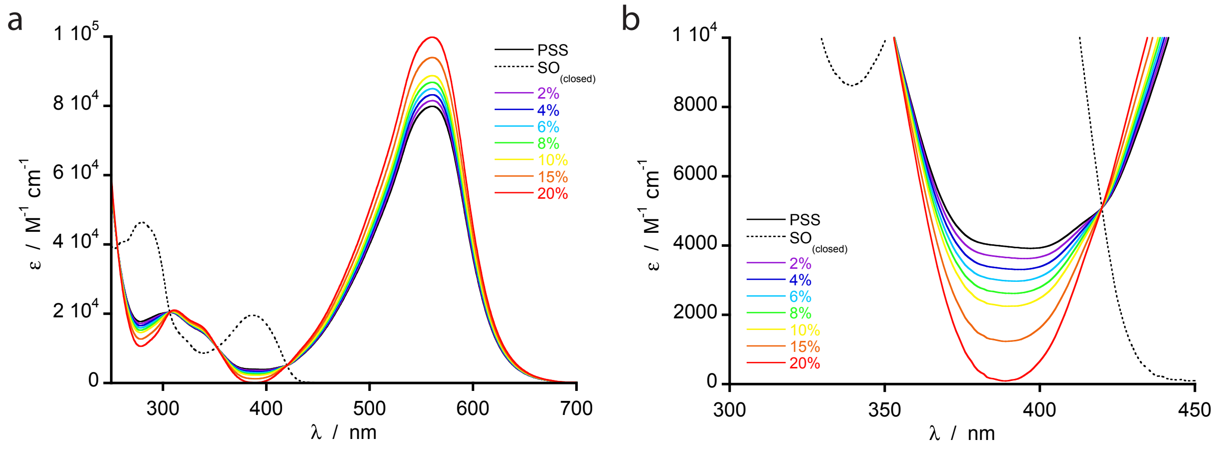

Figure S6. Analysis of the evolution of the spectral shape assuming different contributions of **SO$_{(closed)}$** to the PSS spectrum.

S6. Rates of thermal ring closure

In the UV-vis spectrometer, a vigorously stirred solution of **SO** (3.3 µM, 3 mL) at 25 °C was irradiated by 405 nm LED (2.1 W/cm^2, 2.5 s), to establish the PSS, then the irradiation was stopped and the absorbance changes at 590 nm were monitored over time. The decay was fitted to a mono-exponential function. The results indicate that dielectric constants of solvents have important effect on the kinetic rate of thermal ring closure. Additionally, in protic polar solvents (*i.e.* CH_3OH, EtOH, 2-propanol), the thermal relaxation is much faster even to the extent that it is within the spectrometer response time and cannot be measured, whereas non-polar solvents like toluene and cyclohexane, significantly slow down the process.

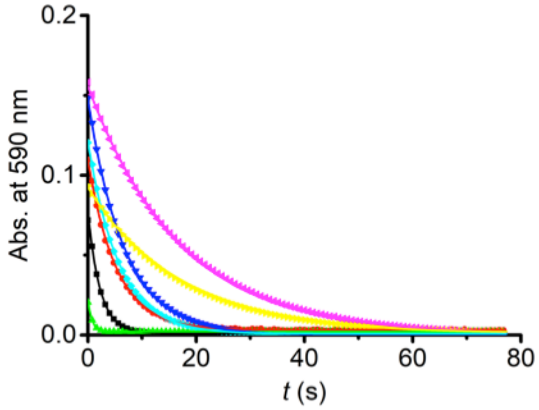

Figure S7. Kinetic traces of thermal ring closure of **SO$_{(open)}$** in different solvents (scatters) and corresponding mono-exponential fits (lines). CH_3CN, black square; fit, black line. Acetone, red circle; fit, red line. 2-Propanol, green up triangle; fit, green line. CH_2Cl_2, navy down triangle; fit, navy line. THF, green diamond; fit, blue line. Toluene, magenta left triangle; fit, magenta line. Cyclohexane, yellow right triangle; fit, yellow line.

Table S3. Half lifetime of thermal ring closure of **SO$_{(open)}$** in different solvents.

Solvent	Dielectric constant κ[a]	$t_{1/2}$ (s)	R^2 of mono-exponential fit
**CH_3CN**	36.6 (20 °C)	1.55	0.9991
**CH_3OH**	32.6	–[b]	–[b]
**EtOH**	24.6	–[b]	–[b]
**acetone**	20.7	3.62	0.9991
**2-propanol**	18.3	0.53	0.9808
**CH_2Cl_2**	8.9	4.69	0.9998
**THF**	7.5 (22 °C)	3.99	0.9998
**toluene**	2.4	11.91	0.9999
**cyclohexane**	2.0	11.55	1.0000

[a]The values at 25 °C (unless otherwise stated in parentheses) are extracted from references.[5,6] [b]The process was too fast to be recorded.

S7. Fluorescence lifetime measurements

The fluorescence lifetime was determined by time correlated single photon counting (TCSPC) using the FS5-TCSPC unit (Edinburgh Instruments) with a picosecond pulsed diode laser (473.4 nm ± 0.5 nm). Once a time-resolved fluorescence decay and the instrumental response function were measured, a reconvolution fit analysis was carried out using the software Fluoracle (Edinburgh Instruments) by fitting in a mono-exponential function: Fit = $A + Be^{(-t/\tau)}$, where A is the calculated background, B is the calculated pre-exponential factor, and the goodness of fit is given as χ^2.

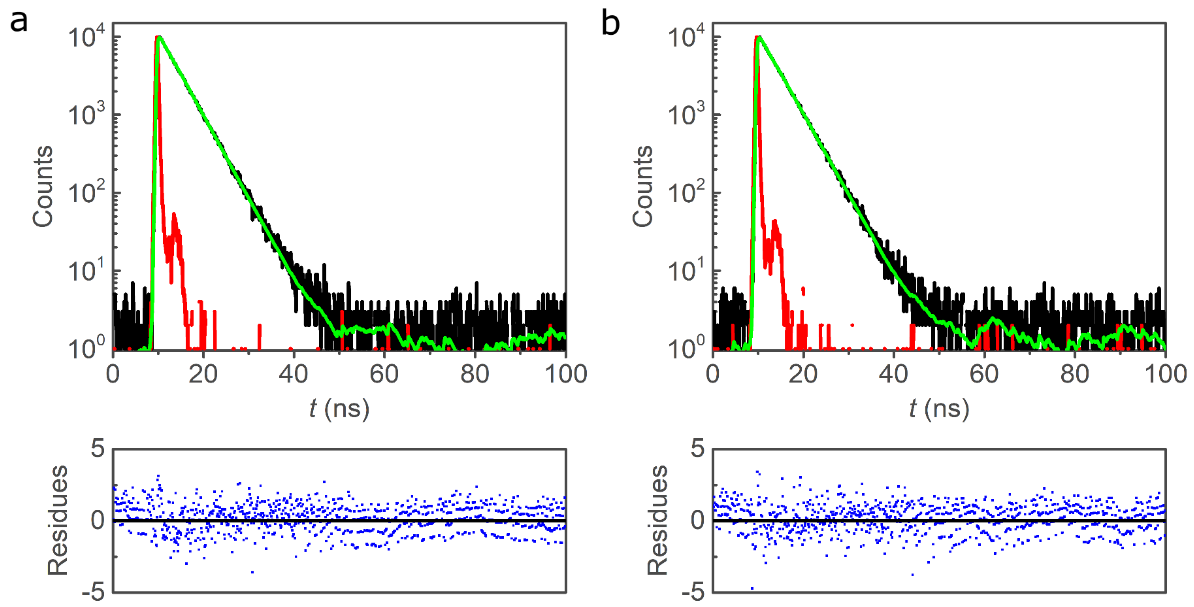

Figure S8. The fluorescence decays of **5'-Atto565** and the mono-exponential fits. a) Top: fluorescence decay of **5'-Atto565** in PBS buffer, black; instrument response function, red; mono-exponential fit, green. Bottom: residues after the fit. The residues for each of the compounds are randomly scattered around the axis y = 0 without an apparent pattern, indicating the presence of a single fluorescence decay. b) Fluorescence decay of **5'-Atto565** in CH_3OH, exponential fit and residues after the fit.

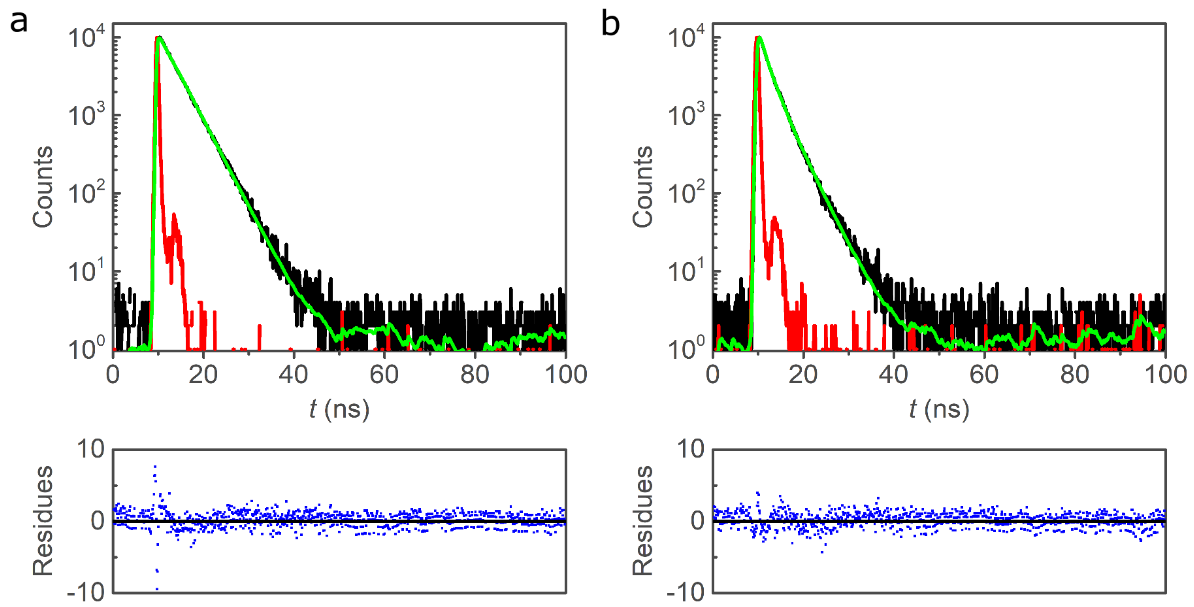

Figure S9. The fluorescence decays of **Dyad 1** and the mono-exponential fits. a) Top: fluorescence decay of **Dyad 1** in PBS buffer, black; instrument response function, red; mono-exponential fit, green. Bottom: residues after the fit. The residues for each of the compounds are randomly scattered around the axis y = 0 without an apparent pattern, indicating the presence of a single fluorescence decay. b) Fluorescence decay of **Dyad 1** in CH_3OH, exponential fit and residues after the fit.

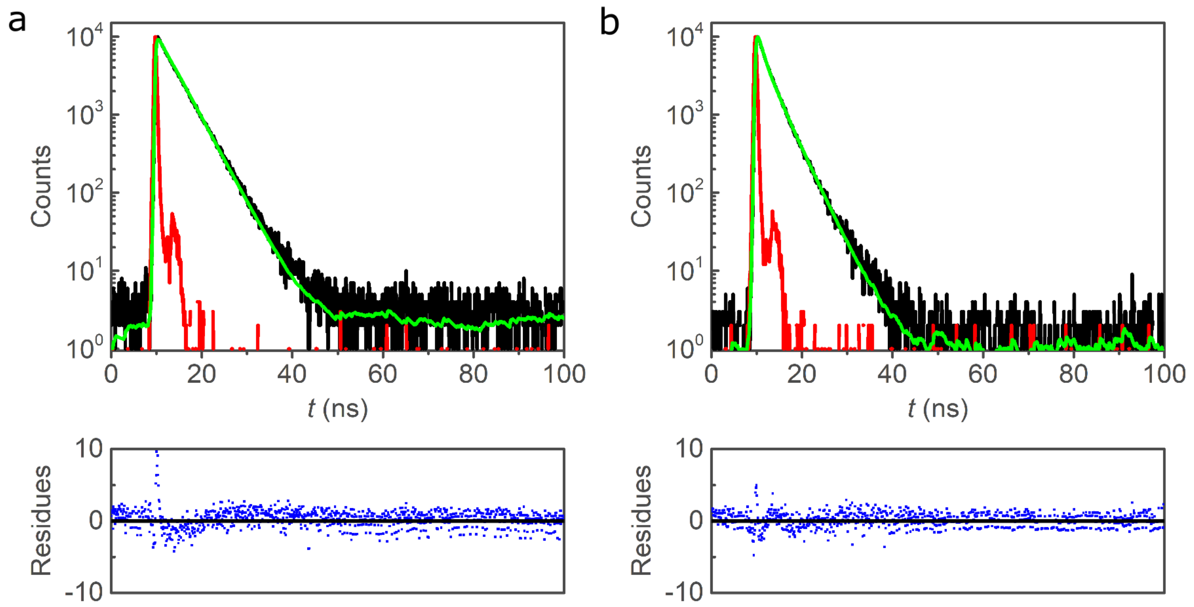

Figure S10. The fluorescence decays of **Dyad 2** and the mono-exponential fits. a) Top: fluorescence decay of **Dyad 2** in PBS buffer, black; instrument response function, red; mono-exponential fit, green. Bottom: residues after the fit. The residues for each of the compounds are randomly scattered around the axis *y* = 0 without an apparent pattern, indicating the presence of a single fluorescence decay. b) Fluorescence decay of **Dyad 2** in CH_3OH, exponential fit and residues after the fit.

S8. Re-absorption corrected fluorescence absolute quantum yields

The absolute fluorescence quantum yields were measured using a SC-30 integrating sphere module (Edinburgh Instruments) and the re-absorption effect was corrected when possible. For **5'-Atto565** in PBS or CH_3OH, **Dyad 1** and **2** in CH_3OH, the excitation wavelength was 520 nm, the wavelength step size 0.1 nm, and integration time 0.2 s. Three scans were repeated for both the sample solution and the solvent (Figure S11). The scattering region between 512 and 528 nm, and emission region between 540 and 770 nm were chosen for the calculation of the observed quantum yields.

Due to the low fluorescence quantum yields of **SO, Dyad 1** and **Dyad 2** in PBS, the scattering spectra were measured with a neutral density filter behind the excitation monochromator to protect the detector, and the emission spectra were obtained separately in a standard way. Specifically, for **SO** in CH_2Cl_2, 405 nm was used as the excitation light. Once the scattering region was scaled accordingly to match the overlapped region with the emission, the scattering region between 392 and 420 nm and emission between 460 and 850 nm were used to calculate the observed quantum yield, 5.1%. For **Dyad 1** and **Dyad 2** in PBS buffer, the excitation source was 520 nm, and similar measurements gave quantum yields of 3.6% and 4.3%, respectively.

The above procedures give the observed quantum yields, but the true fluorescence quantum yields might be higher due to re-absorption of dyes with a significant spectral overlap between absorption and emission. Practically, the emission spectra of the sample are measured in both the standard fluorescence module and the integrating sphere. Once the tails of the two spectra are normalized (Figure S11), the difference in the areas is calculated, denoted as a = (true em./obs. em. − 1), the fraction reduced by re-absorption. The re-absorption corrected fluorescence quantum yield is then calculated using the formula, $QY^{true} = \frac{QY^{obs}}{1-a+a*QY^{obs}/100}$. For highly fluorescent dyes, *i.e.* **5'-Atto565**, **Dyad 1** and **Dyad 2** in CH_3OH, the re-absorption corrected fluorescence absolute quantum yields are only slightly higher than the uncorrected values. On the other hand, it was impossible to acquire the values for the other compounds because of difficulties to get sensible a values due to their low emission.

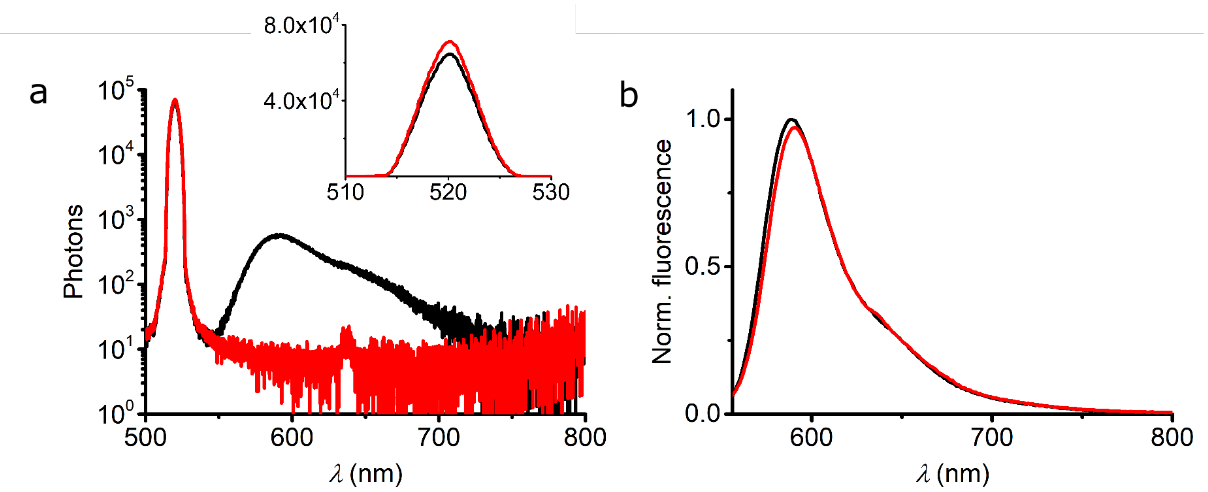

Figure S11. Representative spectra used in the calculation of the absolute fluorescence quantum yield. a) Spectra used in the calculation of the observed quantum yield using an integrating sphere, *y*-axis displayed in the $\log_{10}$ scale. PBS solution of **5'-Atto565**, black; PBS blank solvent, red. The inset is the zoom-in in the scattering region, *y*-axis displayed in the linear scale for clarity. b) Normalized fluorescence spectra using a standard fluorescence module (black) and an integrating sphere (red), and the percentage of the area difference *a*, caused by re-absorption, was used to calculate the absolute quantum yield.

S9. Quantum yields of photochemical reactions

Quantum yield of ring opening

A cyclohexane solution of **SO** (10.0 μM, 2.0 mL) was placed in the sample holder of a UV-vis spectrometer and stirred at 10 °C. While it was irradiated with pulses of mic-LED-405 (28.1 mW, 100 ms pulse, 200 ms interval), a kinetic trace of absorption intensity at 560 nm was recorded and processed in Matlab to give the time-dependent absorbance change (Figure S12).

The ring-opening quantum yield of **SO** was obtained using the following equation,[7] $\phi_O = \frac{mVN_Ahc}{P\lambda(1-10^{-A})\varepsilon_{prod}d}$, where *m* is the slope of the linear fit at the initial rise stage (0.329 ± 0.012 s^{-1} in Figure S12), *V* is the sample volume (2.0 mL), N_Ahc are the constants, *P* is the power intensity (28.1 ± 0.3 mW), *λ* is the excitation wavelength (405 nm), *A* is the absorbance at the excitation wavelength, ε_{prod} is the estimated molar extinction coefficient of $\mathbf{SO}_{\mathbf{(open)}}$ at 560 nm (8.9 ± 0.5 × 10^4 M^{-1} cm^{-1}), *d* is the path length of the cuvette (1 cm). The molar extinction coefficient was derived based on the assumption of 90 ± 5% photoconversion from the closed to open form at the PSS. The calculation gives the quantum yield of ring opening, ϕ_o = 8.0 ± 0.7%. In DCM at 10 °C a value of ϕ_o = 7.7 ± 0.6% is obtained. This value is consistent with what was observed from the TR-IR measurement, *i.e.* 7.4%.

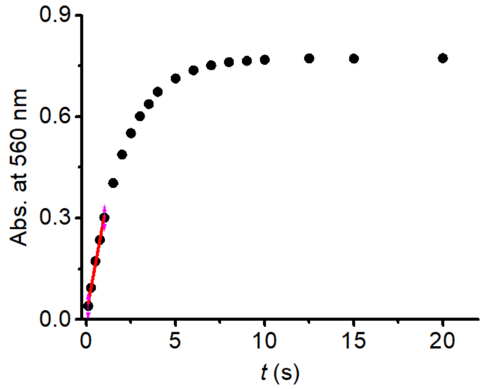

Figure S12. The initial part of the reconstructed ring opening kinetics of **SO**, and the linear fit. The slope *m* = 0.329 ± 0.012 (used in the calculation of the quantum yield of ring opening), R^2 = 0.998.

Quantum yield of ring closure

The photoreversion traces of ring closure monitored at 560 nm were obtained using the same conditions as those of thermal ring closure, except that the sample was given pulses of mic-LED-525 (68.5 mW, 100 ms irradiation, 400 ms interval) while recording the spectrum (Figure 4b). The monitoring of 560 nm absorbance is based on the assumption that the closed form does not absorb at this wavelength, and upon exposure to 525 nm LED, the ring closing reaction is accelerated without significant side-reactions. Thus, the disappearance of the starting material/open isomer is at the same rate as the appearance of the product/closed isomer. The trace was reconstructed in Matlab.

To calculate the ring closing quantum yield of **SO**, the measurements were carried out as follows: after irradiated with mic-LED-405 (150 mW, 2.5 s) to generate the open form, the sample solution was exposed to a pulse of mic-LED-525 (68.5 mW, 100 ms) to initiate photochemical reversion. After the pulse, the sample continued to ring close thermally. During the experiment, the absorbance at 560 nm was recorded as a function of time, giving a kinetic trace. The above procedure was repeated using increasing duration of 525 nm irradiation (200 ms, 400 ms etc.). Each photochemical trace was subtracted from the thermal trace, and the average of the first few values after the light scattering region in each processed photochemical trace gave the difference caused by irradiation of 525 nm of a specified period (Figure S13). By using the equation,[7] $\phi_C = \frac{mVN_Ahc}{P\lambda(1-10^{-A})\varepsilon_{SO(open)}d}$, where m is the linear fit for the disappearance of $\mathbf{SO}_{\mathbf{(open)}}$ by monitoring at 560 nm (m = 0.108 ± 0.002 s^{-1} in Figure S13), P is the power intensity (68.5 ± 1.7 mW), λ is the excitation wavelength (525 nm), A is the absorbance at excitation wavelength (0.60), and $\varepsilon_{SO(open)}$ is the molar absorption coefficient of $\mathbf{SO}_{\mathbf{(open)}}$ at 560 nm (8.9 ± 0.5 × 10^4 M^{-1} cm^{-1}). The calculation gave ϕ_C = 1.1 ± 0.1 %. This value agrees with that obtained from the TR-IR measurements, *i.e.* 1.4% – 2.3%.

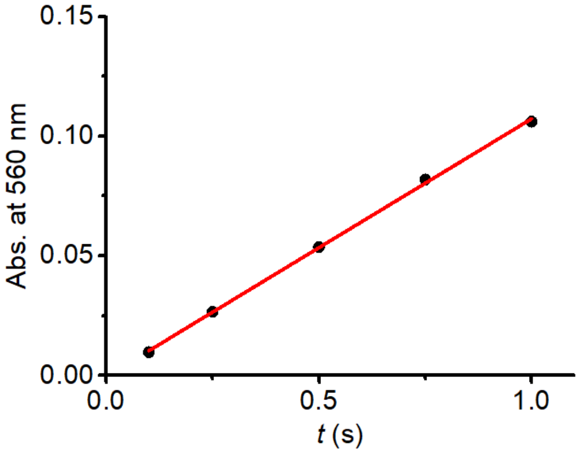

Figure S13. The initial part of the reconstructed ring closing kinetics of **SO**, and the linear fit. The slope m = 0.108 ± 0.002 (used in the calculation of the quantum yield of ring closing), R^2 = 0.999.

S10. FRET efficiency (E) calculations

The following equations were used:[8]

$$E = \frac{1}{1 + (r/R_0)^6} \quad (1)$$

$$R_0 = 0.2108\left[\frac{\kappa^2 \Phi_D J(\lambda)}{n^4}\right]^{\frac{1}{6}} \quad (2)$$

$$J(\lambda) = \int f_D \varepsilon_A(\lambda)\lambda^4 d\lambda \quad (3)$$

where r is the distance between the donor and acceptor, R_0 is the Förster radius (distance at which the energy transfer efficiency is 50%), κ^2 is the dipole orientation factor (assumed to be 2/3 given free rotation between the donor and acceptor), ϕ_D is the fluorescence quantum yield of the donor in the absence of the acceptor (79.5%), n is the refractive index of the solvent (1.34 for PBS buffer), $J(\lambda)$ is the spectral overlap integral, f_D is

the normalized donor emission spectrum in PBS, ε_A is the acceptor molar extinction coefficient, and λ is the wavelength. Because the spectrum of **SO(open)** in PBS is unavailable due to the rapid thermal recovery, we choose the **SO(open)** form in cyclohexane instead to calculate the spectral overlap, assuming there are only minor solvatochromic changes. The calculated Förster radius is 66.4 Å for the dyads.

The distance between the switch and dye in **Dyad 1** was simulated by optimizing the molecular geometry in *Hyperchem* with a molecular mechanics calculation in an MM^+ force field. With a distance of max. 15.4 Å and min. 6.8 Å, the FRET efficiency is *ca.* 100% for both the cases.

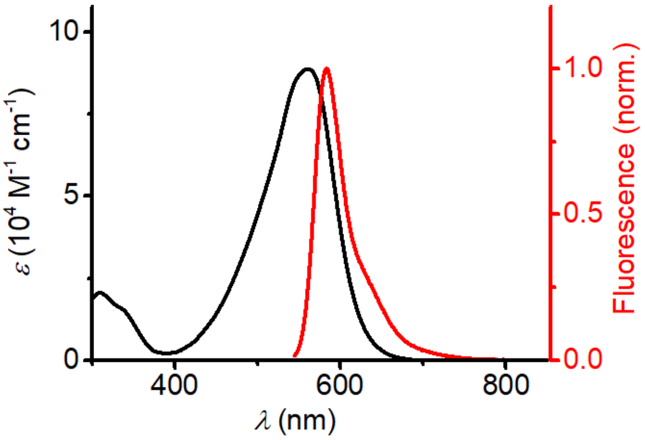

Figure S14. Overlap of the reconstructed spectrum of **SO(open)** in cyclohexane (black) and normalized emission spectrum of **5'-Atto 565** in PBS buffer (red). Based on the spectral overlap, a Förster radius of 66.4 Å was obtained using PBS as the medium. This indicates that FRET efficiencies of ~100% are expected for interchromophore distances smaller than 16 Å.

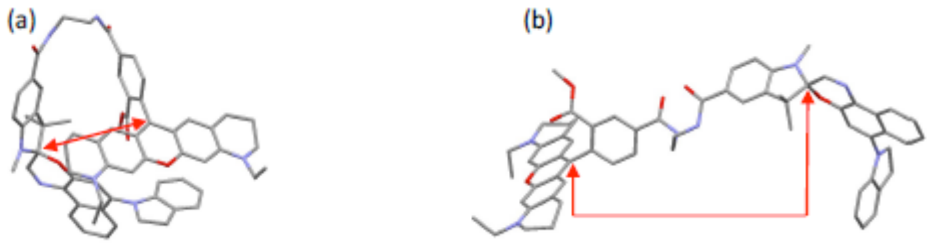

Figure S15. Modeled structures of **Dyad 1**. The distance between the C atoms marked in red was used to calculate FRET efficiency. (a) folded conformer, distance: 6.8 Å. (b) unfolded conformer, distance: 15.4 Å.

S11. Fatigue resistance in the cuvette

The fatigue resistance by thermal reversion is measured as follows: a cyclohexane solution of **SO** (10 µM, 2.0 mL) was placed in the sample holder of a UV-vis spectrometer and stirred at 25 °C. The samples were irradiated using mic-LED-405 (2.1 W/cm^2) for 2.5 s at the beginning of each cycle. Immediately after irradiation, a kinetic trace of absorption intensity at 590 nm was recorded until the ring closure was completed. The above procedure was repeated for > 100 cycles. The raw data were treated in Matlab to extract the maximal and minimal absorbance after 405 nm excitation for each cycle.

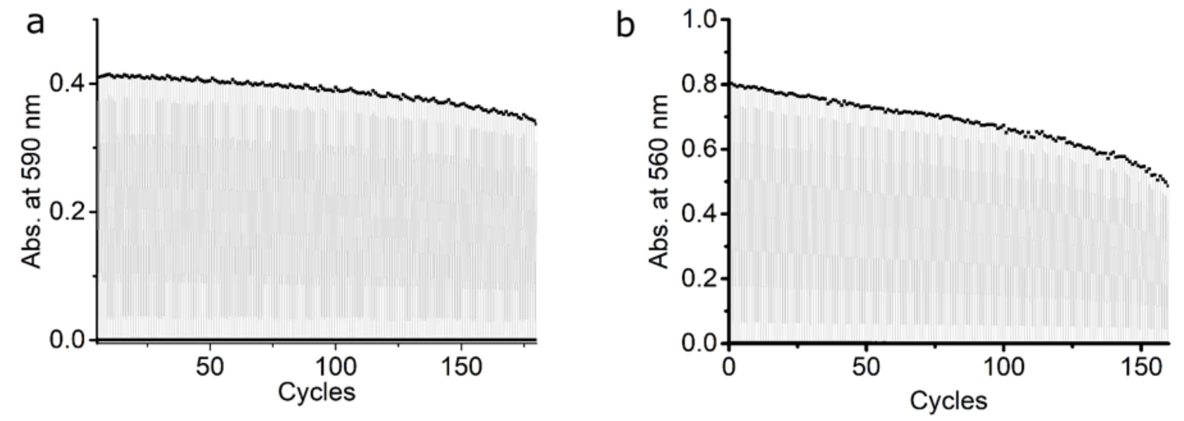

Figure S16. Fatigue resistance of **SO** in the cuvette. a) Fatigue resistance of **SO** in CH_2Cl_2 at 25 °C. Each cycle consists of excitation at 405 nm (2.5 s, 2.1 W/cm^2) and thermal relaxation. The data were treated in Matlab. b) Photochemical fatigue resistance of **SO** in cyclohexane at 10 °C. Each cycle consists of excitation at 405 nm (2.5 s, 2.1 W/cm^2), 500 ms interval, excitation at 525 nm (30 s, 0.97 W/cm^2) and further 5 s waiting until the absorbance returned to ~0.

S12. DFT calculations

DFT calculation where performed as described before,[9] briefly: The molecular structures were optimized at the DFT B3LYP/6-31+G(d,p) level of theory using the software package Gaussian 16.[10] Solvation in CH_2Cl_2 was applied using the polarizable continuum model (PCM).[11] All structures are confirmed ground-state minima according to the analysis of their analytical frequencies computed at the same level, which show no imaginary frequencies. On these minima, the vertical transition energies were calculated by time-dependent density functional theory (TD-DFT) at DFT cam-B3LYP/6-311+G(2d,p) level of theory with the PCM solvation in CH_2Cl_2. The choice of functionals and basis sets are based on the conclusions of reference.[12]

The calculated geometries for switch **SO** in the closed and all four open isomers CTT, CTT, TTT, and TTC are presented at the end of this section together with the Cartesian coordinates. All structures are optimized at the DFT B3LYP/6-31+G(d,p) level of theory.

Isomer stabilities

The relative stabilities of the open-form isomers were calculated and are summarized in Table S3. As reported before for similar spirooxazines,[12] only the TTC and CTC forms are likely to be observed experimentally.

Table S4. Relative stabilities and species distributions of **SO$_{(open)}$** stereoisomers.

SO$_{(open)}$ isomer	Relative Stability (kcal mol^{-1})	Speciation (%)
**TTC**	0	91.3
**CTC**	1.40	8.6
**TTT**	6.45	< 0.1
**CTT**	6.66	<0.1

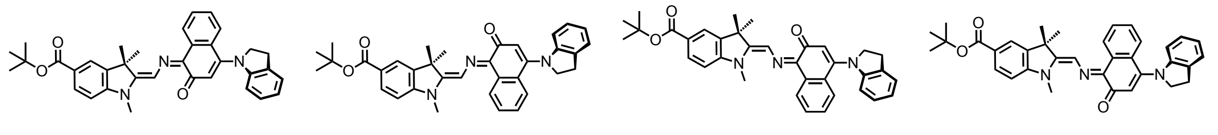

SO$_{(open)}$ (TTC-form) **SO$_{(open)}$** (TTT-form) **SO$_{(open)}$** (CTC-form) **SO$_{(open)}$** (CTT-form)

Figure S17. Structures of the most likely isomers of **SO$_{(open)}$**.

Calculated IR and UV-vis spectra

The calculated IR-spectra were obtained at the DFT B3LYP/6-31+G(d,p) level of theory. Similarly, the UV-vis spectra were calculated as vertical transitions as described before; Figures S18 and S19.

Although the calculated spectra are bound not to be a perfect match to the experimental data, it is obvious from the comparison in Figure S18 (UV) that the different isomers of the open form will not be sufficiently different to quantify the various contributions and whether the photo-conversion proceeds through a sequential mechanism. This limitation has hampered the study of the ultra-fast dynamics of spiropyrans and spirooxazines in the past. A completely different picture exists when considering the vibrational transitions. It is apparent from Figure S21 (IR) that the isomers of the open form have noticeable differences. It follows that transient IR is the ideal technique to investigate the putative mechanism of photo-conversion of spirooxazines and spiropyrans. This is discussed in detail in the following section.

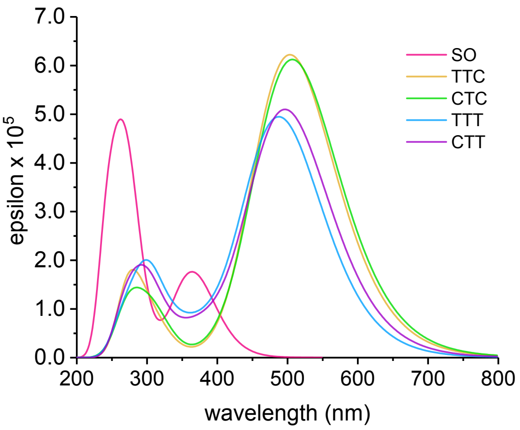

Figure S18. Predicted UV-vis spectra of **SO** in the closed form (SO) and the open form (all four isomers).

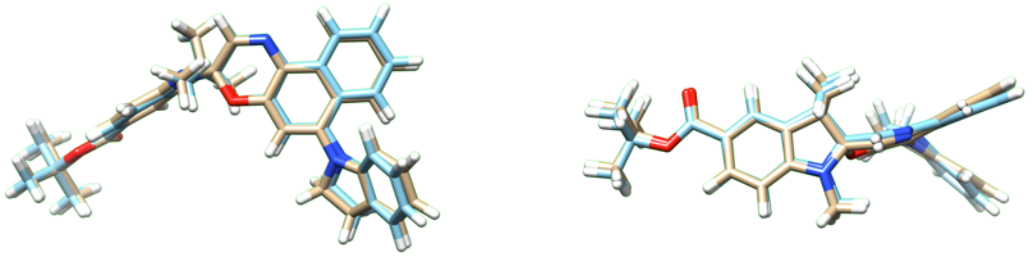

Figure S19. Comparison between the ground-state (light grey) and singlet-excited state (light blue) optimized geometries of $\mathbf{SO}_{\mathbf{(closed)}}$. Structures were aligned to the best match using Chimera.

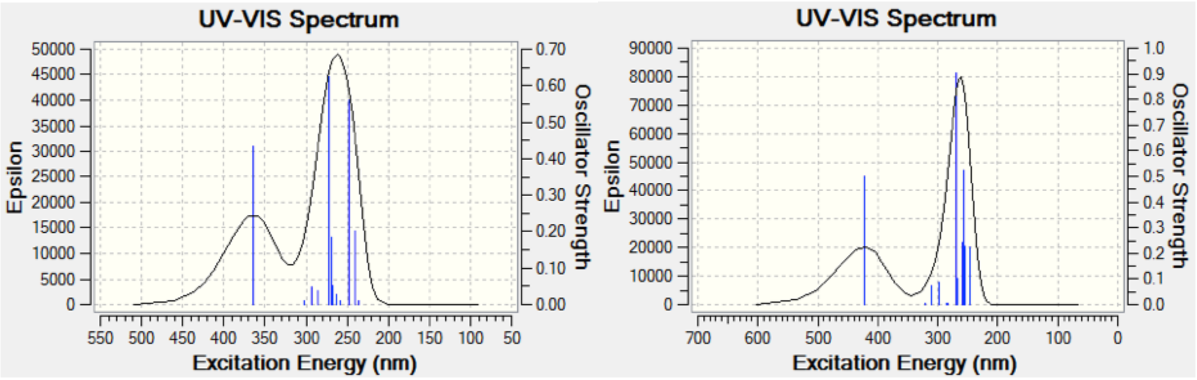

Figure S20. Comparison between the predicted absorption spectra of the vertical electronic transition for the ground-state (left) and singlet-excited state (right) optimized geometries of $\mathbf{SO}_{\mathbf{(closed)}}$.

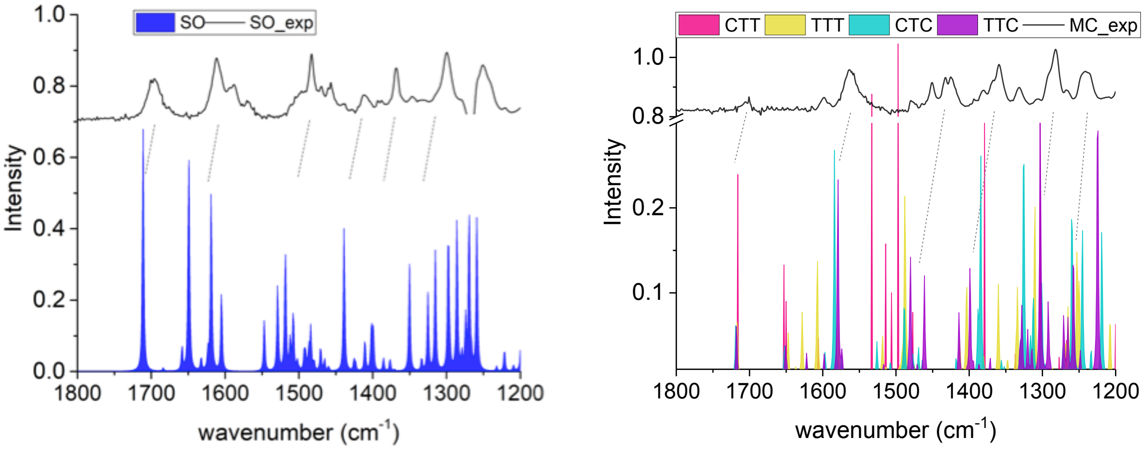

Figure S21. Predicted IR spectra for **SO** in the closed form (left) and the open form (all four isomers, right). Frequencies calculated at the DFT B3LYP/6-31+G(d,p) level of theory. No scaling was applied but dotted lines are drawn as guides for the eye. Notice that the different isomers, notably those that are calculated to be unstable, have very different IR signatures (e.g. the massive CTT band ca. 1500 cm^{-1}). The experimental spectra are shown at the top.

Coordinates of the optimized structures

SO$_{(closed)}$

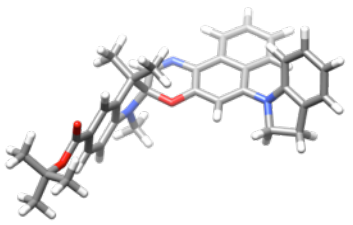

Symbol	X	Y	Z
C	-8.82887000	-1.21430800	-1.09367100
C	-8.64812900	-2.10217100	-0.02923700
C	-7.60035500	-1.93873500	0.89181600
C	-6.71065000	-0.84888400	0.74514900
C	-6.88287000	0.03549200	-0.30002500
C	-7.93689900	-0.14388600	-1.21720400
H	-9.63618400	-1.36606500	-1.80170700
H	-9.32545000	-2.94077400	0.08253500
C	-6.13495800	1.31208800	-0.65626900
C	-4.60784000	1.19965900	-0.55134100
H	-4.32512300	1.03963000	0.49372700
H	-4.21952400	0.36732400	-1.14109900
H	-4.12283100	2.12314500	-0.88477300
C	-6.63194400	2.45761700	0.26051900
H	-6.37768700	2.22012700	1.29746600
H	-6.15190900	3.40811900	0.00479900
H	-7.71686200	2.58584300	0.20106000
N	-7.92379200	0.86548000	-2.17211700
C	-6.63410200	1.52961400	-2.14897800
C	-6.74596400	2.97806900	-2.58220300
C	-4.88235600	1.46439700	-3.86564400
H	-7.56599300	3.54164800	-2.14030900
C	-4.94893300	2.84018000	-4.02215100
C	-3.91396300	0.68687600	-4.52935800
C	-4.01670000	3.48934200	-4.89381400
C	-2.97599000	1.28728200	-5.35488000
H	-3.90060400	-0.38070700	-4.34381500
C	-3.03162800	2.70995600	-5.58673000
C	-4.06765300	4.89148300	-5.12168900
C	-2.18227900	3.36098900	-6.52155400
C	-3.20660800	5.49567800	-6.01488100
H	-4.80862300	5.47396900	-4.58640600
H	-1.46818600	2.76987300	-7.08246500
C	-2.26411800	4.72239800	-6.73303800
H	-3.26273400	6.56797600	-6.17933400
H	-1.60940100	5.19911300	-7.45637000
H	-5.90440500	-0.72997600	1.46172500
C	-7.38061000	-2.87453300	2.02485400
O	-8.30114000	-3.85786400	2.06230500
O	-6.46288200	-2.75624600	2.83002300
C	-8.68909300	0.76438700	-3.40820600
H	-8.35596200	-0.07297900	-4.03518400
H	-9.74849900	0.63355700	-3.17345100
H	-8.58224000	1.69099900	-3.97559400
O	-5.76333500	0.79528000	-3.06832200
N	-5.96996900	3.57655900	-3.40822500
C	-0.61411700	0.59346000	-5.78767400
C	0.03989000	-0.47292900	-6.43593900
C	0.10641000	1.52694600	-5.03731500
C	1.42012300	-0.59988500	-6.35993200
C	1.50068900	1.38593400	-4.96811600
H	-0.39001800	2.33673900	-4.51487300
C	2.15960300	0.34014400	-5.62087000
H	1.92347800	-1.42327700	-6.86027500
H	2.07256400	2.10285800	-4.38553900
H	3.23898000	0.24743500	-5.54906200
N	-2.00389100	0.50462800	-6.00900900
C	-8.30163900	-4.92166800	3.09485800
C	-7.01559600	-5.74798600	2.99487500
H	-6.14042700	-5.16148800	3.27715100
H	-7.08991400	-6.61021300	3.66563800
H	-6.88026800	-6.12222800	1.97496800
C	-9.51768700	-5.76410900	2.70108600
H	-9.63140300	-6.59967200	3.39825700
H	-10.43149500	-5.16276100	2.72975900
H	-9.39801400	-6.16947000	1.69172100
C	-8.49827400	-4.30428300	4.48305900
H	-7.63586900	-3.70705400	4.78166600
H	-9.39191800	-3.67198100	4.49677100
H	-8.63873100	-5.10604900	5.21542700
C	-2.29866600	-0.87797600	-6.45023700
H	-3.17641000	-0.88536100	-7.10009600
H	-2.49285100	-1.53150600	-5.58663800
C	-0.99599600	-1.31316500	-7.15162600
H	-1.02849200	-1.05867700	-8.21968300
H	-0.82262100	-2.38913800	-7.06936900

SO, open form TTC

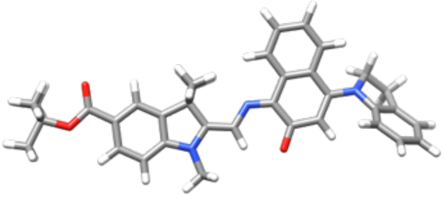

Symbol	X	Y	Z
C	-8.96271800	-0.98721700	-0.69689800
C	-8.66815200	-2.02872900	0.18563900
C	-7.47568500	-2.04274100	0.92911900
C	-6.55104200	-0.98668200	0.78761500
C	-6.83321500	0.05000500	-0.08239500
C	-8.03017700	0.04554300	-0.81607700
H	-9.88833600	-1.00002100	-1.26047700
H	-9.37350500	-2.84319600	0.29848000
C	-6.03399200	1.29550000	-0.43126800
C	-5.81811600	2.18671700	0.81588900
H	-5.29704100	3.10376000	0.53552500
H	-6.77290500	2.45315100	1.27930800
H	-5.21826000	1.64511600	1.55418800
C	-4.67634800	0.91864400	-1.07353200
H	-4.14277100	1.82011200	-1.37961000
H	-4.06673200	0.37061200	-0.34807700
H	-4.81959200	0.28129500	-1.95136800
N	-8.07992000	1.19392300	-1.61717400
C	-6.96302900	1.96805700	-1.44852300
C	-6.78838900	3.16057800	-2.14022100
C	-6.24276800	5.73728200	-3.51455700
H	-7.54720700	3.49448500	-2.83788500
C	-5.38573000	5.04119900	-2.51818400
C	-5.80658800	7.03056300	-3.98157600
C	-4.09996900	5.66644100	-2.15632000
C	-4.64215800	7.63792600	-3.58164900
H	-6.49586400	7.54602300	-4.64069100
C	-3.71190200	6.92459700	-2.69832600
C	-3.20097700	5.00530900	-1.29314000
C	-2.42035800	7.42105300	-2.42476600
C	-1.95007100	5.53346100	-1.00014100
H	-3.50325800	4.05517300	-0.87150400
H	-2.09084100	8.33844200	-2.89453400
C	-1.54617600	6.74248800	-1.58436600
H	-1.27987600	4.99675000	-0.33470600
H	-0.55672900	7.14555700	-1.39115500
H	-5.63394500	-1.00946200	1.36684100
C	-7.13927200	-3.14469700	1.87532200
O	-8.10644600	-4.07610000	1.93486900
O	-6.09630400	-3.18065800	2.51716300
C	-9.17757400	1.53111000	-2.51644800
H	-9.94366500	0.76105500	-2.45833000
H	-9.61908400	2.49066100	-2.23243600
H	-8.81772000	1.59501000	-3.54743800
O	-7.33748900	5.25925400	-3.90863300
N	-5.67266100	3.89167600	-1.93261200
C	-4.83503100	9.51813700	-5.19133300
C	-5.08432700	10.88509800	-4.99019500
C	-5.03311600	8.92551600	-6.44223000

C	-5.54159500	11.67655800	-6.03893100
C	-5.49651000	9.72993400	-7.49001500
H	-4.83065000	7.87206800	-6.60260500
C	-5.75009100	11.09359400	-7.29786400
H	-5.73709100	12.73438800	-5.88432200
H	-5.65271200	9.28531500	-8.46863700
H	-6.10468700	11.70107100	-8.12497900
N	-4.30572000	8.92262900	-4.02195900
C	-8.01460400	-5.28043600	2.79923000
C	-6.82418000	-6.14107800	2.36572500
H	-5.87474500	-5.64502100	2.57110500
H	-6.84872100	-7.08857600	2.91378600
H	-6.88491800	-6.36649700	1.29616900
C	-9.33589900	-5.99620700	2.50907900
H	-9.39441000	-6.91721200	3.09670700
H	-10.18704400	-5.36186300	2.77464500
H	-9.41144000	-6.25692100	1.44904600
C	-7.93645100	-4.86120400	4.27033700
H	-6.99698100	-4.35421700	4.49390000
H	-8.76927800	-4.19724100	4.52332300
H	-8.01043100	-5.75313400	4.90098300
C	-3.96239900	10.02510600	-3.07059700
H	-2.89536900	10.26123400	-3.14652400
H	-4.18138500	9.72223600	-2.04639300
C	-4.80038400	11.23360800	-3.54650300
H	-4.25354100	12.17257000	-3.42555300
H	-5.73592800	11.31816600	-2.97835700

SO, open form CTC

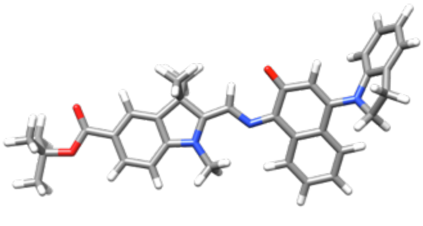

Symbol	X	Y	Z
C	-8.92595500	-0.69105300	-0.49667900
C	-8.66373200	-1.88753100	0.17521100
C	-7.48409100	-2.06650300	0.91716600
C	-6.54042900	-1.02058800	0.99430600
C	-6.79178400	0.16773600	0.33410100
C	-7.97103800	0.32421000	-0.40905400
H	-9.84737600	-0.57203700	-1.05450000
H	-9.38688700	-2.69266300	0.12605100
C	-5.97987600	1.44578300	0.24380900
C	-5.76536600	2.08079800	1.63851400
H	-5.22129600	3.02567100	1.55384200
H	-6.72006900	2.27312800	2.13652500
H	-5.17899500	1.40292400	2.26657000
C	-4.61551500	1.20077200	-0.44703000
H	-4.06080300	2.13771300	-0.54967400
H	-4.01625700	0.51113600	0.15558700
H	-4.74809700	0.76408600	-1.44114000
N	-7.99277100	1.60152100	-0.99284400
C	-6.88947200	2.32315400	-0.63066400
C	-6.54934400	3.63309700	-0.95114100
C	-5.78010300	6.37717000	-1.79891500
H	-5.58463500	3.98262200	-0.60156100
C	-7.08168800	5.69101700	-2.01409900
C	-5.60333500	7.67450300	-2.40569700
C	-8.15097300	6.43995300	-2.69833700
C	-6.57634900	8.32464500	-3.12332600
H	-4.61045700	8.10020000	-2.31504800
C	-7.92128300	7.74444000	-3.22088200
C	-9.44824300	5.89608900	-2.80546000
C	-9.00547100	8.47075300	-3.75525300
C	-10.49373300	6.61184000	-3.37543600
H	-9.62053100	4.90463000	-2.40693200
H	-8.85363900	9.48980300	-4.08615100
C	-10.27726900	7.91676900	-3.84035300
H	-11.48243400	6.16615600	-3.43791300
H	-11.09555500	8.49804800	-4.25425300
H	-5.63525900	-1.16974400	1.57352700
C	-7.18377000	-3.33431600	1.64289700
O	-8.15715200	-4.24850600	1.49212300
O	-6.16157600	-3.50512000	2.29600500
C	-9.07740500	2.04633300	-1.86202000
H	-8.67440200	2.69500800	-2.63643600
H	-9.54586700	1.17310100	-2.31649900
H	-9.82678500	2.60561100	-1.29253500
O	-4.83291700	5.83862000	-1.17139200
N	-7.36670400	4.45415500	-1.64257700
C	-5.27834600	10.42759400	-3.36560100
C	-4.69464200	11.02893300	-4.49209900
C	-4.87264600	10.77251100	-2.07268600
C	-3.69144400	11.98046000	-4.33878400
C	-3.86014000	11.72856500	-1.92946800
H	-5.32975400	10.31754000	-1.20062700
C	-3.27080600	12.33172200	-3.04731200
H	-3.23705700	12.44534700	-5.20971500
H	-3.53552100	12.00925800	-0.93165700
H	-2.49007200	13.07445400	-2.91433400
N	-6.32407300	9.55266900	-3.74393000
C	-8.10115300	-5.59650100	2.11346400
C	-6.90055200	-6.37283800	1.56425600
H	-5.95636400	-5.93616500	1.89214800
H	-6.94945300	-7.40673200	1.92118000
H	-6.92468400	-6.39044100	0.46992500
C	-9.41493800	-6.22865000	1.64812400
H	-9.49762600	-7.24457900	2.04554100
H	-10.27184500	-5.64779200	2.00262000
H	-9.45522300	-6.28022200	0.55585300
C	-8.07221500	-5.46568100	3.63917800
H	-7.13839000	-5.02160200	3.98618400
H	-8.90982900	-4.85220800	3.98629600
H	-8.17268400	-6.46045900	4.08538300
C	-5.31689200	10.44883300	-5.74215500
H	-5.54896500	11.20058500	-6.50129900
H	-4.64640900	9.70993400	-6.19999900
C	-6.59935100	9.78043400	-5.19668600
H	-7.44951600	10.46186000	-5.31052200
H	-6.83921300	8.83725200	-5.68818600

SO, open form TTT

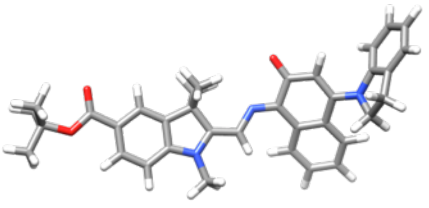

Symbol	X	Y	Z
C	-8.71267500	-1.21816300	-1.01565500
C	-8.34851900	-2.24471400	-0.14140800
C	-7.35665100	-2.05665800	0.83600500
C	-6.71129900	-0.80594400	0.94361900
C	-7.06382400	0.21726500	0.08403600
C	-8.05623900	0.00852700	-0.88803700
H	-9.48130500	-1.38897000	-1.76058000
H	-8.84147100	-3.20633900	-0.21909300
C	-6.54391700	1.64288300	-0.02204800
C	-6.81974900	2.43234700	1.28055800
H	-6.48797700	3.46646400	1.16767100
H	-7.88626700	2.42656700	1.52665100
H	-6.27479800	1.97234800	2.11123500
C	-5.03194200	1.66605300	-0.35587000
H	-4.69243300	2.69646900	-0.47526400
H	-4.47189300	1.19526200	0.45852300
H	-4.82448600	1.11275800	-1.27718100
N	-8.22670400	1.18257700	-1.62983500
C	-7.38513300	2.17654900	-1.18900300
C	-7.37191900	3.44224100	-1.74393200
C	-5.07771500	6.26732800	-1.28233500
H	-8.04307000	3.65202100	-2.57396500

C	-6.34607500	5.59220200	-1.71747200
C	-4.58645900	7.32536500	-2.14109200
C	-7.24694600	6.36348800	-2.59767400
C	-5.31327800	7.84509100	-3.18166900
H	-3.56696400	7.64471600	-1.96031700
C	-6.73509700	7.47391600	-3.32927100
C	-8.63450800	6.12138100	-2.64052300
C	-7.61886800	8.27519500	-4.07286200
C	-9.49181100	6.91445300	-3.40076700
H	-9.05972600	5.33635200	-2.02852800
H	-7.24287200	9.15285800	-4.58296100
C	-8.98205800	7.99354400	-4.12783000
H	-10.55745400	6.70559000	-3.40242300
H	-9.64330700	8.63009100	-4.70752900
H	-5.94720800	-0.67181800	1.70219300
C	-6.95020000	-3.13752600	1.77710800
O	-7.63857000	-4.27510900	1.57354500
O	-6.08574300	-2.99144900	2.63315600
C	-9.16861300	1.34134500	-2.72933600
H	-9.68009900	0.39816200	-2.90951400
H	-9.91494100	2.10473200	-2.48763300
H	-8.64209100	1.63232600	-3.64331800
O	-4.40685200	5.86165700	-0.31305500
N	-6.47776300	4.35846400	-1.29727300
C	-3.61799200	9.54922600	-3.81604500
C	-2.85730600	9.69420000	-4.98716100
C	-3.24350100	10.18531800	-2.62883400
C	-1.70297500	10.47035200	-4.98190700
C	-2.07870500	10.96216200	-2.63365300
H	-3.83825800	10.08962100	-1.72707800
C	-1.31062300	11.10716500	-3.79506600
H	-1.11139000	10.58175600	-5.88666900
H	-1.77508200	11.46486900	-1.72002400
H	-0.41263200	11.71723300	-3.77839700
N	-4.78287100	8.78317900	-4.06513900
C	-7.42097500	-5.50355300	2.37809500
C	-5.98755400	-6.00672500	2.18278100
H	-5.25929600	-5.32136400	2.61820100
H	-5.88147100	-6.98277200	2.66739900
H	-5.76788200	-6.13123900	1.11755000
C	-8.42693400	-6.48138900	1.76610500
H	-8.36677400	-7.44482700	2.28108700
H	-9.44777900	-6.09979400	1.86460200
H	-8.21487900	-6.64251700	0.70475900
C	-7.75551000	-5.23104500	3.84773800
H	-7.04242200	-4.54083900	4.30017700
H	-8.76343500	-4.81369700	3.93933000
H	-7.72762700	-6.17495300	4.40180500
C	-4.91905600	8.63913300	-5.54784400
H	-5.60767300	9.39931400	-5.93237000
H	-5.31132100	7.65321500	-5.79749300
C	-3.49738800	8.89238400	-6.09779900
H	-3.52557200	9.42081800	-7.05437300
H	-2.95777700	7.94922800	-6.25380700

SO, open form CTT

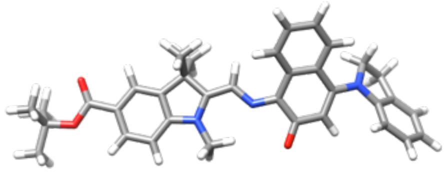

Symbol	X	Y	Z
C	-8.40651100	-0.97203900	-1.14079800
C	-8.46080200	-1.97895700	-0.17353300
C	-7.57002200	-1.99764600	0.91328600
C	-6.59715900	-0.98196000	1.04013300
C	-6.53593000	0.01802100	0.08842200
C	-7.43244600	0.01729400	-0.98982800
H	-9.10186600	-0.96982400	-1.97230300
H	-9.20512000	-2.76119400	-0.26226200
C	-5.61938500	1.22139200	-0.03977000
C	-5.77818400	2.18087000	1.16421400
H	-5.14950200	3.06762200	1.04065000
H	-6.81671800	2.50531100	1.27647000
H	-5.47425300	1.67285700	2.08480700
C	-4.14130600	0.78832200	-0.18806900
H	-3.49079400	1.65882700	-0.31426900
H	-3.82064800	0.25220100	0.71069000
H	-4.00775200	0.12724700	-1.04918000
N	-7.18338400	1.11647100	-1.81958700
C	-6.14522200	1.87007800	-1.33439700
C	-5.57865100	3.02218100	-1.85605900
C	-6.53334000	5.32517800	-4.54576400
H	-4.75310400	3.43462200	-1.27920800
C	-5.60459700	4.73003300	-3.52919300
C	-6.40197400	6.74678600	-4.78403200
C	-4.34330600	5.44880300	-3.26746700
C	-5.37240900	7.49858400	-4.27659800
H	-7.21151300	7.21146300	-5.33419100
C	-4.22960100	6.82255900	-3.63028100
C	-3.19457400	4.79056000	-2.78457800
C	-2.99139300	7.47209900	-3.48550700
C	-1.98155100	5.45809800	-2.62777800
H	-3.23231600	3.72849400	-2.58095300
H	-2.88510300	8.49804300	-3.81398300
C	-1.87891400	6.80830400	-2.97317800
H	-1.11362900	4.91709300	-2.26281400
H	-0.93369700	7.33293000	-2.87426700
H	-5.91738700	-1.00815400	1.88543500
C	-7.60912000	-3.06103300	1.95663900
O	-8.57840800	-3.96384800	1.72654300
O	-6.83851900	-3.09437500	2.90896000
C	-7.98635800	1.35116000	-3.01983300
H	-7.61209800	2.23281600	-3.53308700
H	-7.92784700	0.47401100	-3.67259600
H	-9.03182200	1.51027400	-2.73459900
O	-7.45468100	4.66425200	-5.06692000
N	-6.06089200	3.64388600	-2.95519100
C	-6.08688900	9.61984300	-5.35818700
C	-6.52854300	10.83029300	-4.80054200
C	-6.34050500	9.30697200	-6.69689500
C	-7.24141000	11.74010200	-5.57451500
C	-7.06088700	10.22838700	-7.46647500
H	-5.98547800	8.38086400	-7.13545700
C	-7.51072800	11.43492000	-6.91705600
H	-7.58617700	12.67649900	-5.14407800
H	-7.26421000	10.00160800	-8.50899700
H	-8.06441600	12.13762300	-7.53225900
N	-5.32254200	8.88375900	-4.42018900
C	-8.82873300	-5.12450400	2.61790900
C	-7.59855900	-6.03661400	2.64294800
H	-6.75245100	-5.55501800	3.13471300
H	-7.84382400	-6.95364700	3.18852800
H	-7.30825300	-6.31514900	1.62481800
C	-10.00591200	-5.82011400	1.93027800
H	-10.29379800	-6.70946600	2.49877000
H	-10.87054700	-5.15204000	1.87176100
H	-9.73478800	-6.13025900	0.91653300
C	-9.22808200	-4.63361800	4.01273700
H	-8.39673500	-4.13967500	4.51727600
H	-10.06991900	-3.93694600	3.94632600
H	-9.54380400	-5.49039700	4.61689200
C	-5.03153000	9.79014700	-3.26613400
H	-4.03986800	10.23905200	-3.38847100
H	-5.04940900	9.22993200	-2.33127000
C	-6.11694000	10.88692300	-3.34680500
H	-5.72542900	11.86161400	-3.04388200
H	-6.96826200	10.65233700	-2.69468700

S13. Ultrafast spectroscopy

Experimental procedures

The sample of **SO** used for time-resolved IR and transient absorption had an absorbance of about 1.3 at 405 nm in CD_2Cl_2. TA samples were prepared with a typical absorbance of 0.5 at 405 nm in CH_2Cl_2. The UV-vis absorption and FT-IR spectra were measured before and after the experiments to check for any noticeable decomposition. A flow system, along with cell raster scanning, was set up to minimize the interference from the long-lived $\mathbf{SO_{(open)}}$ form and effects of photodegradation.

The instruments have been described in detail in references.[13,14] Briefly:

ULTRA (TR^{M}PS-TA)

The TA measurements were performed on the ULTRA[15] setup in the Time-Resolved Multiple Probe Spectroscopy mode[13] (hereafter TR^{M}PS) at the Central Laser Facility (STFC Rutherford Appleton Laboratories, Oxfordshire, UK). The TA experiments were driven by a 10 kHz repetition rate Ti:Sapph amplifier (Thales) as a probe source, producing 40 fs pulses at 800 nm. A fraction of the Ti:Sapph laser output was used to generate a white light continuum (WLC) in a CaF_2 plate to probe the sample transient absorption. The WLC was passed through the photoexcited spot on the sample (probe spot size approx. 80 μm) and dispersed through the 0.25 m (f/4) grating spectrograph and detected using a silicon diode array (Quantum Detectors). The long-pass filter was used in front of the spectrograph to block the scattered excitation radiation. No referencing was required for the TA experiments thank to sufficiently stable white light. The excitation source for the TA experiments was the second harmonic (400 nm) of the 1 kHz titanium sapphire amplifier (Spectra Physics Spitfire XP, 100 fs pulse length), pulse energy at sample attenuated down to 0.2–1 μJ and focused down to 150 × 150 μm^2 spot). Both ULTRA amplifier and Spitfire amplifier were optically synchronized by sharing the same seed from 68 MHz Ti:Sapph oscillator. The seed beam was delayed with an optical delay line before the 1 kHz amplifier to accommodate for the 100 fs − 14.7 ns time delays between pump and probe. To go beyond 14.7 ns and up to 100 μs, subsequent seed pulses are selected from the 68 MHz seed pulse train accompanied by the appropriate setting of the optical delay line. The polarization of the excitation beam at sample was set to be at 54.7° with respect to the probe in TA experiments.

LIFETime (TR^{M}PS-TRIR)

The TRIR experiments were performed in TR^{M}PS mode on LIFEtime setup at the Central Laser Facility STFC.[14] The LIFEtime setup is based on a dual-amplifier 100 kHz Yb:KGW laser system (Pharos & Pharos SP, Light Conversion Ltd.). The two Yb:KGW amplifiers are optically synchronized through sharing the same oscillator seed beam. The output beam of Pharos amplifier is split 50/50 and used to pump the two identical OPAs (Orpheus ONE, Light Conversion Ltd.) followed with GaSe DFG stages. The two identical OPA+DFG systems produce two independently tunable mid-IR probe beams for TRIR experiments, each covering > 200 cm^{-1} useable bandwidth. The Pharos SP amplifier is used to pump Orpheus HP OPA generating the excitation beam for the TRIR experiments tunable from UV to mid-IR. In the current study the excitation wavelength was set at 320, 405, and 590 nm. The repetition rate of the output beam of Pharos SP amplifier was divided down programmatically by an internal pulse picker to excitation repetition rate of 0.1–1 kHz to enable Time-Resolved Multiple Probe Spectroscopy (TR^{M}PS) mode. Relative pump–probe timing control between the two Yb:KGW amplifiers is programmable from 100 fs to 10 ms, using a combination of oscillator roundtrip timing to achieve steps of 12 ns and translation stage optical delay of the pump similar to our previous work.[13] Here, we opted for optical delay of the OPA output but not of the amplifier seed beam. The excitation beam was focused to approx. 100 μm spot at the sample and overlapped at the sample with the two mid-IR probe beams (each probe beam is approx. 100 μm in size). The excitation pulse energy at sample was set to 0.4–1.0 μJ. The polarization of the excitation beam at the sample was at 54.7° with respect to the probe beams. After the sample, the two probe beams enter independently tunable homemade spectrographs (0.15 m, f/6) and spectra are measured on two 128-element MCT detector arrays (IR Associates). The spectrum acquired by each MCT detector is integrated and digitized by an FPAS system (Infrared Systems Development Corporation). Thanks to the excellent stability of the mid-IR probe beams, no referencing has been applied in the measurements shown here.

For the TR-IR measurements, the sample solution was excited using 405 nm of 0.7 μJ (or 590 nm, 0.6 μJ) at the frequency of 100 Hz (or 1 kHz), and probed in the ca. 1200–1600 and 1400–1800 cm^{-1} regions at 100 KHz. For TA measurements, the sample was pumped at 400 nm (0.2 μJ, 1 KHz repetition rate), and probed in the visible region (10 KHz probe repetition rate).

Data treatment

All data sets were initially corrected for time zero and group velocity dispersion (*i.e.* chirp correction) prior to analysis of the data. The TR-IR data were preliminarily analyzed using the software Surface Xplorer Pro and home-made UltraView.

Single wavenumber traces were fitted using OriginLab to multiexponential functions as in the following equation:

$$y = \sum A_i e^{-x/t_i} + y_0$$

where i is either 1 or 2, x is the time (ns in the time-resolved IR data; ps in the transient absorption data), t_i is the lifetime, A_i is the pre-exponential factor. Figures S22 and S23 show some examples of TRIR and TA single-wavelength data at selected time ranges where the data can be roughly fitted to exponential decays. The data were further analyzed by target analysis using a custom written Matlab script.[16]

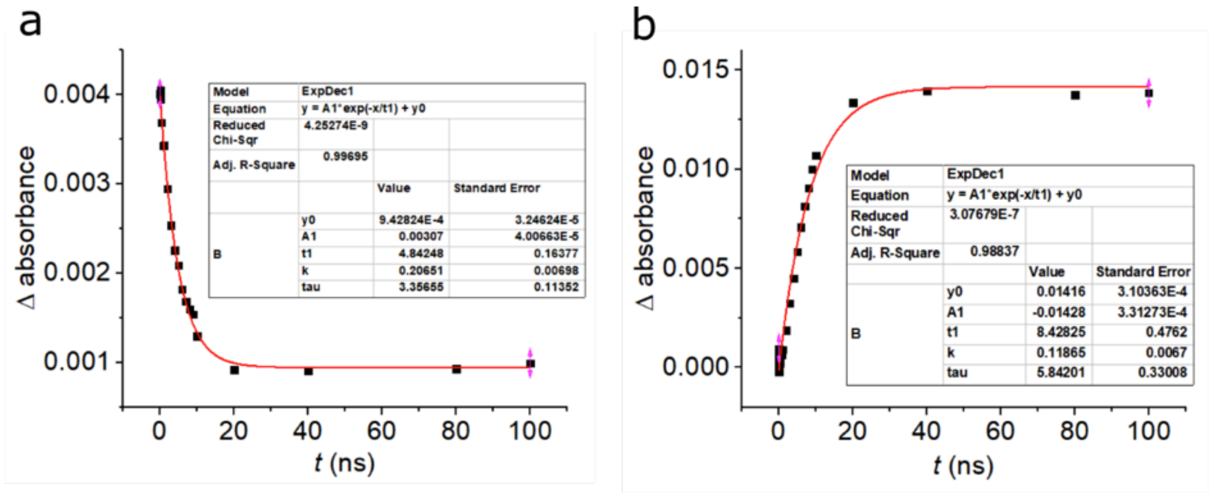

Figure S22. Fitting of TA kinetics at selected single wavelengths to exponential decays, over a selected time range of 0–100 nm. a) kinetics at 432 nm. b) kinetics at 591 nm. The lifetimes from this simple analysis are slightly different from the values obtained from target analysis.

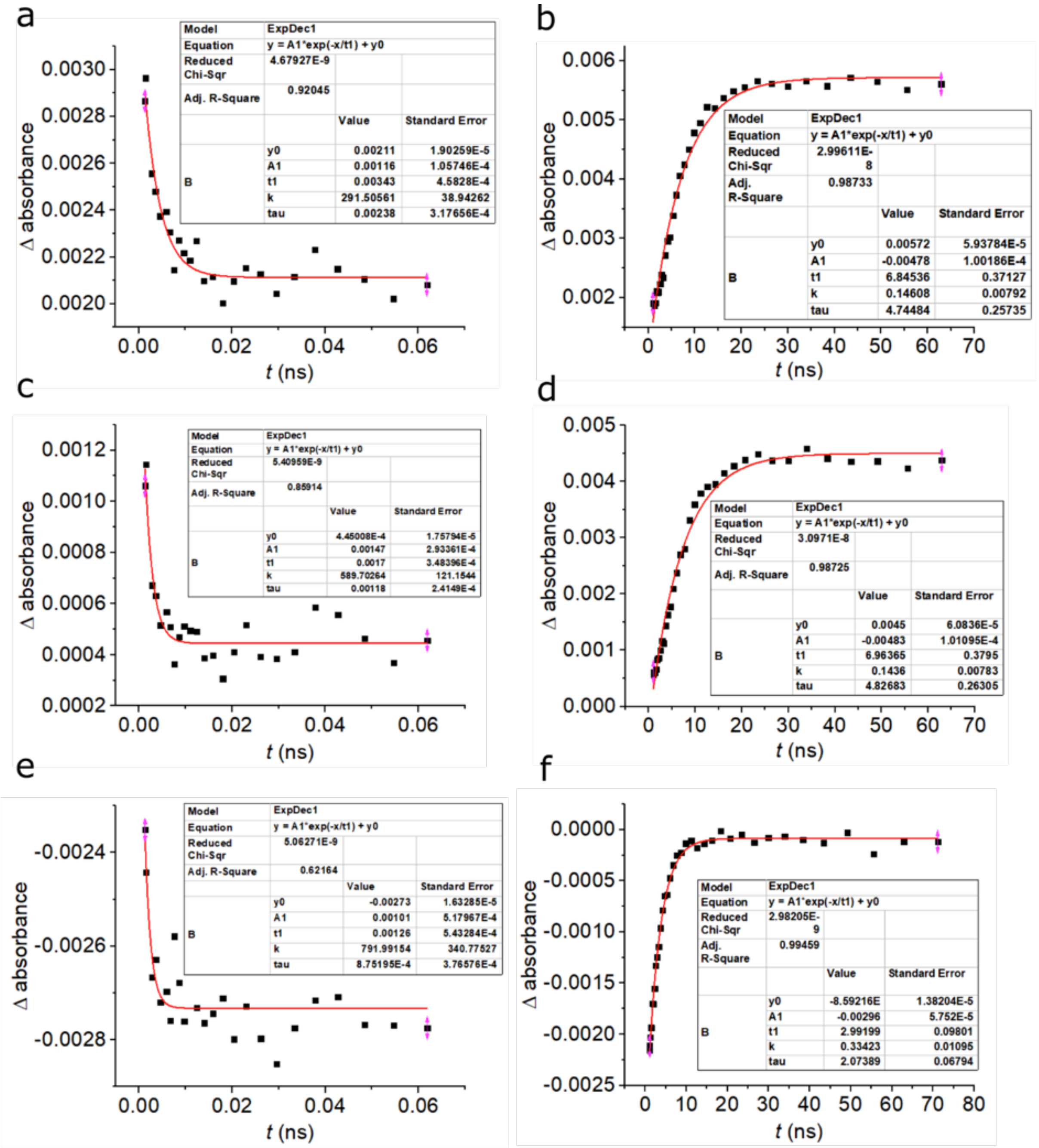

Figure S23. Fitting of TRIR kinetics at selected single wavelengths to exponential decays, over selected time ranges. a, b) kinetics at 1279 cm^{-1}. c, d) kinetics at 1355 cm^{-1}. e, f) kinetics at 1488 cm^{-1}. Some data fit well and the lifetimes agree with those obtained from target analysis, whereas some do not.

Additional results

While TRIR could only be carried out in deuterated CD_2Cl_2 because of intense absorptions from the solvents, TA allowed us to study the influence of different solvents in the ring-opening photochemistry of **SO**. The photo-induced ring-opening of **SO** was additionally measured in acetone, DMSO, THF, and toluene and the resulting TA spectra are presented in Figure S24. Qualitatively, the TA spectra look strikingly similar in term of intermediate species and timescales. The main outlier is DMSO, which appears to hamper the formation of the photoproduct. Additionally, the shape of the excited state absorption is less defined as compared to other solvents, notable CH_2Cl_2 and toluene. In fact, the difference of the spectrum in DMSO is marked enough for the proposed model (see main text) to fail when attempting to simulate the data (in DMSO), suggesting a different reaction pathway. Moreover, when considering that acetone also induces the same features to a lesser extent and one can tentatively suggest that polar solvents are not ideal for this photo-

conversion. From steady state measurements we know that the thermal back reaction is slower (more than 50 times slower cyclohexane to MeOH) and that the fatigue resistance is best in apolar solvents.

Target analysis of the TA spectra in acetone, THF and toluene reveals similar values for the kinetic constants (see Figure S25). Minor differences can be observed on the speciation plots, with the notable difference in accumulation of the X-intermediate for the acetone and toluene cases. Nevertheless, this differences are minor and it can be concluded that solvent has only minor influence on the photo-isomerization reaction of **SO**.

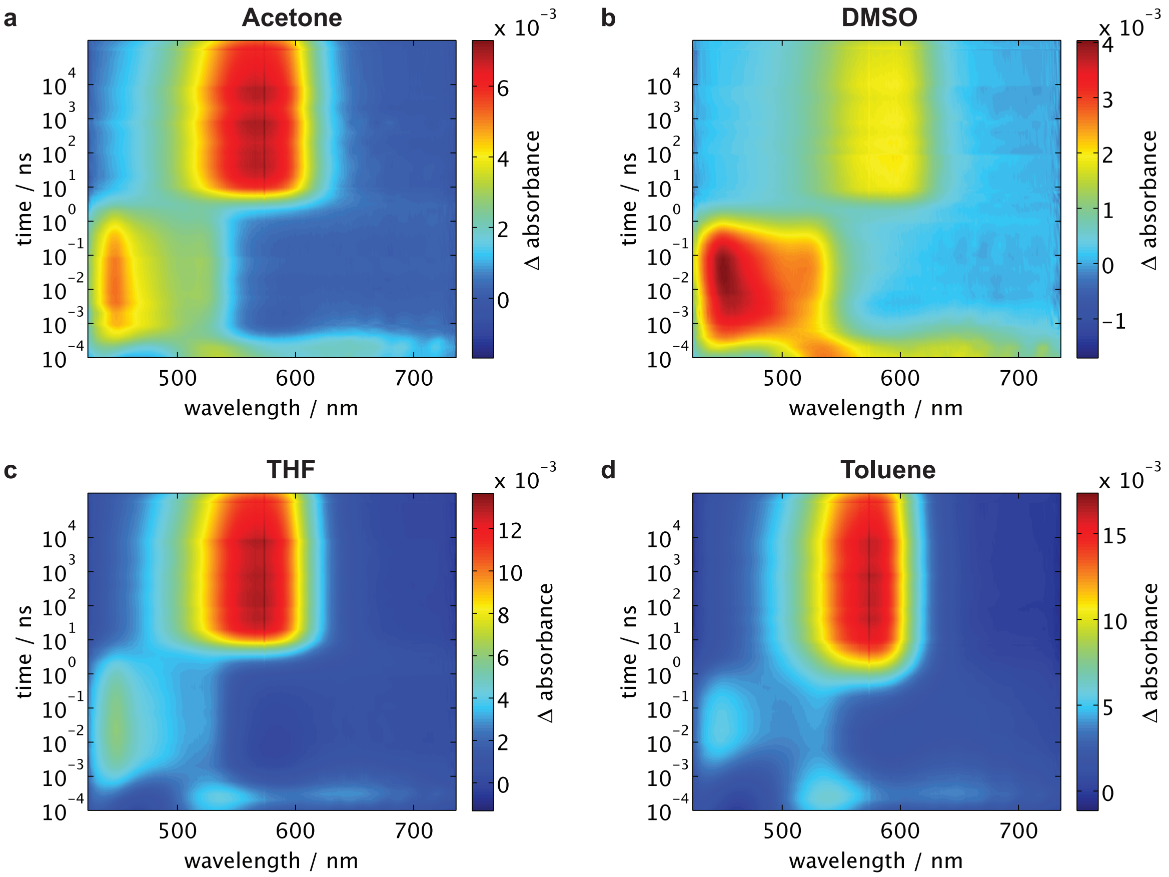

Figure S24. TA spectra corresponding to **SO** in different solvents.

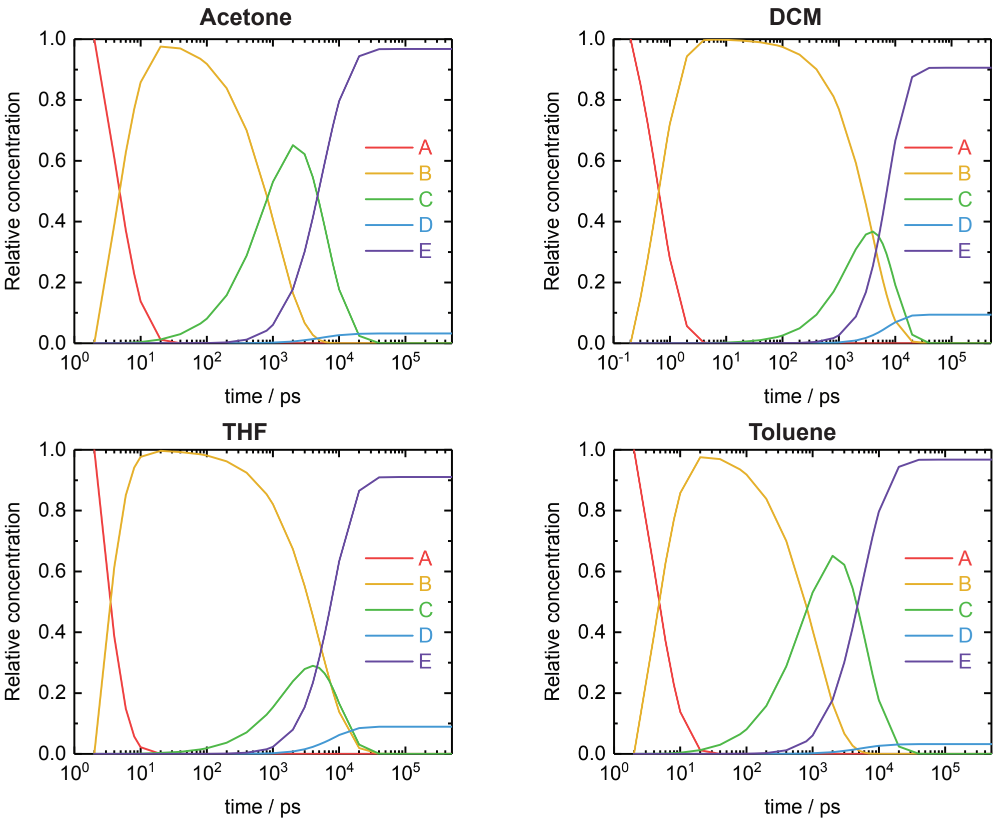

Figure S25. Speciation plots for **SO** in different solvents. Fitted parameters: τ_1 = 1.3; 2.8; 2.1; 4.0 ps. τ_2 = 1.9; 2.3; 5.0; 1.1 ns. τ_3 = 4.2; 4.5; 3.6; 5.2 ns. and τ_4 = 37; 40; 36; 160 ns for acetone, CH_2Cl_2, THF, and toluene correspondingly.

S14. Crystallographic and structural analysis

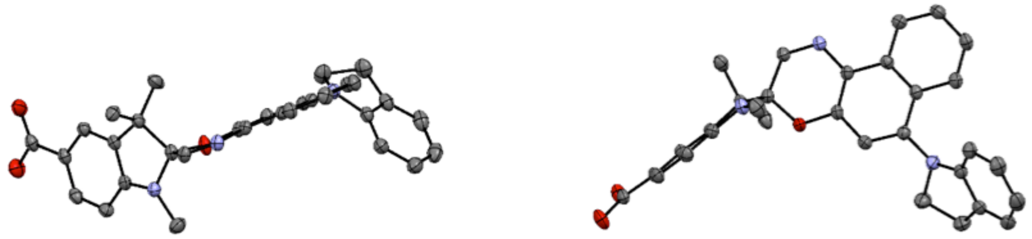

Figure S26. Crystal structure of compound **7** (hydrogens omitted for clarity). The crystals suitable for X-ray diffraction studies were grown from slow evaporation of a solution of compound **7** in a solvent mixture of CH_3OH and $CHCl_3$ at 20 °C. Single crystal data: $C_{31}H_{27}N_3O_3$, M_r = 489.57, triclinic, **P** −1, a = 7.6914(3) Å, b = 8.8535(4) Å, c = 18.0798(9) Å, α = 91.235(4)°, β = 97.2.1(3)°, γ = 92.431(3)°, V = 1218.20(10) Å, data/restraints/parameters: 5026/0/334, R_{int} = 3.4%, final R_1 = 4.07%, wR_2 = 10.51% ($I > 2\sigma(I)$), $\Delta\rho_{min,max}$ = −0.24,+0.29 e Å^3.

Single crystal X-ray diffraction data were collected at 150 K using an Oxford Diffraction/Agilent SuperNovae A (Cu) X-ray source. The raw frame data were integrated and reduced using CrysAlisPro (Agilent Technologies, 2010). The structure was solved using charge flipping[17,18] with SuperFlip method.[19] It was refined by full-matrix least-squares on F^2 in CRYSTALS.[20–22] The crystallographic data have been deposited with the Cambridge Crystallographic Data Centre (CCDC 1812758), and copies of these data can be obtained free of charge *via* www. ccdc.cam.ac.uk/data_request/cif.

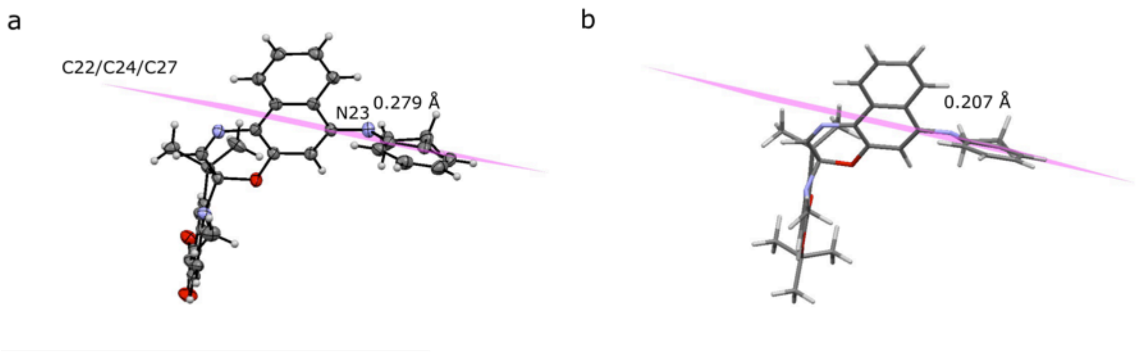

Figure S27. Distorted trigonal configuration adopted by N23 on the naphthoxazine half of compound **7** a) in the solid state; b) from DFT calculations.

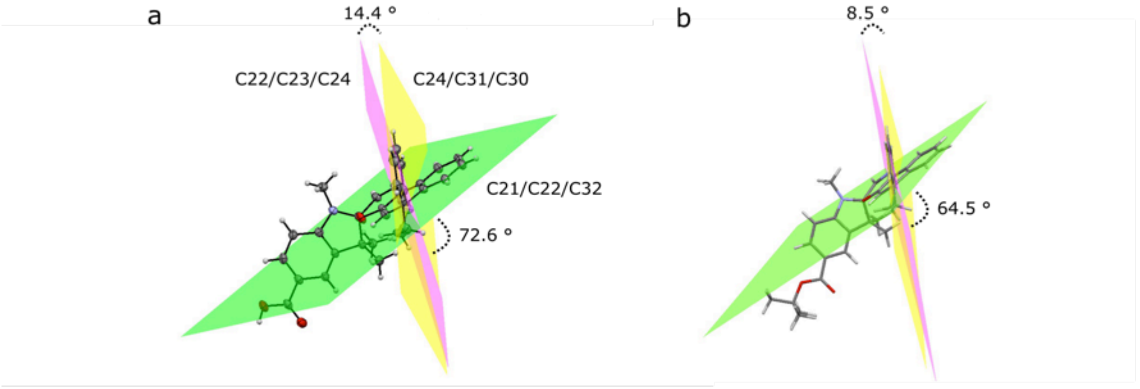

Figure S28. Intersection angles between the planes C21/C22/C32, C22/C23/C24 and C24/C31/C30 a) in the solid state; b) from DFT calculations.

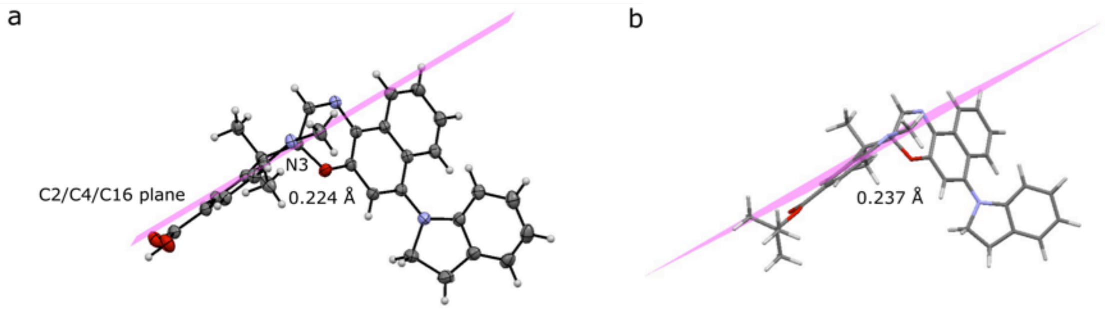

Figure S29. Distorted trigonal configuration adopted by N3 on the indole half of compound **7** a) in the solid state; b) from DFT calculations.

S15. Cell culture and staining procedure

Human embryonic kidney (HEK) cells were cultured in Dulbecco's modified Eagle medium (DMEM), supplemented with 10% fetal calf serum (FCS). The cells were grown to confluence at 37 °C with 5% CO_2 in the presence of a poly-D-lysine-coated microscope coverslip 24 h before imaging. For staining, the dyad was added to the growth medium as DMSO stock solution to reach a final concentration of 5 μM and the cells were incubated for 15 min. The coverslip was taken out of the growth medium, rinsed with dye-free medium, and used for imaging at 37 °C.

S16. Optical microscopy

Images in Figures 7, S31 and S33 below were acquired on a Zeiss 780 confocal microscope, operated with Zen software. The excitation light sources were diode lasers (405 nm, 815 μW at 100%) and (561 nm, 920 μW at 100%). The data were imported to ImageJ (Fiji software), and the color 'magenta hot' was used to present the images.

The fatigue resistance and quenching efficiencies in live cells were analyzed and presented in Figure 8 in the manuscript. The fatigue resistance in cell imaging is defined as how many switching cycles a dyad (as an entire dyad of the switch and dye) can survive before photodegradation. This property is expressed as the normalized fluorescence of the same field of view over consecutive cycles, relative to the fluorescence readout at the first bright state. The quenching efficiency, on the other hand, describes the performance of the switch *without* considering the effects from possible aggregation, motion of the cells, and decomposition of the dye. This presents the extent to which the switch depletes the fluorescence in a single cycle, and is defined as QE = 1 − fluorescence of the dark state/fluorescence of the bright state of the same cycle.

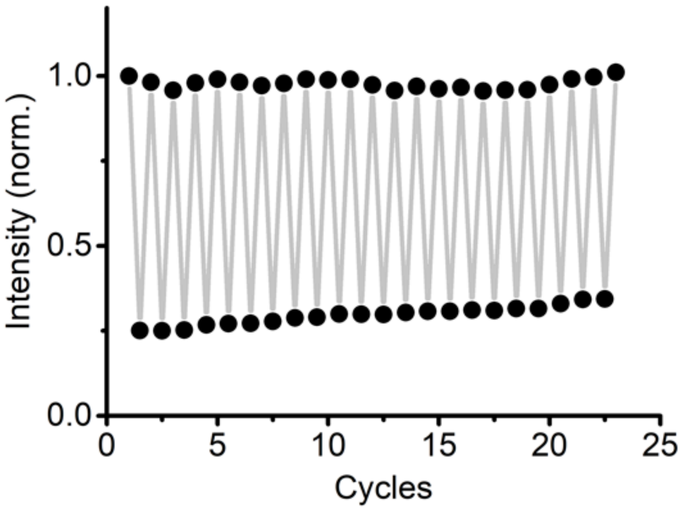

Figure S30. Fatigue resistance of **Dyad 1** in live HEK cells under a confocal microscope, using fluorescence quantification of the images in Figure S31.

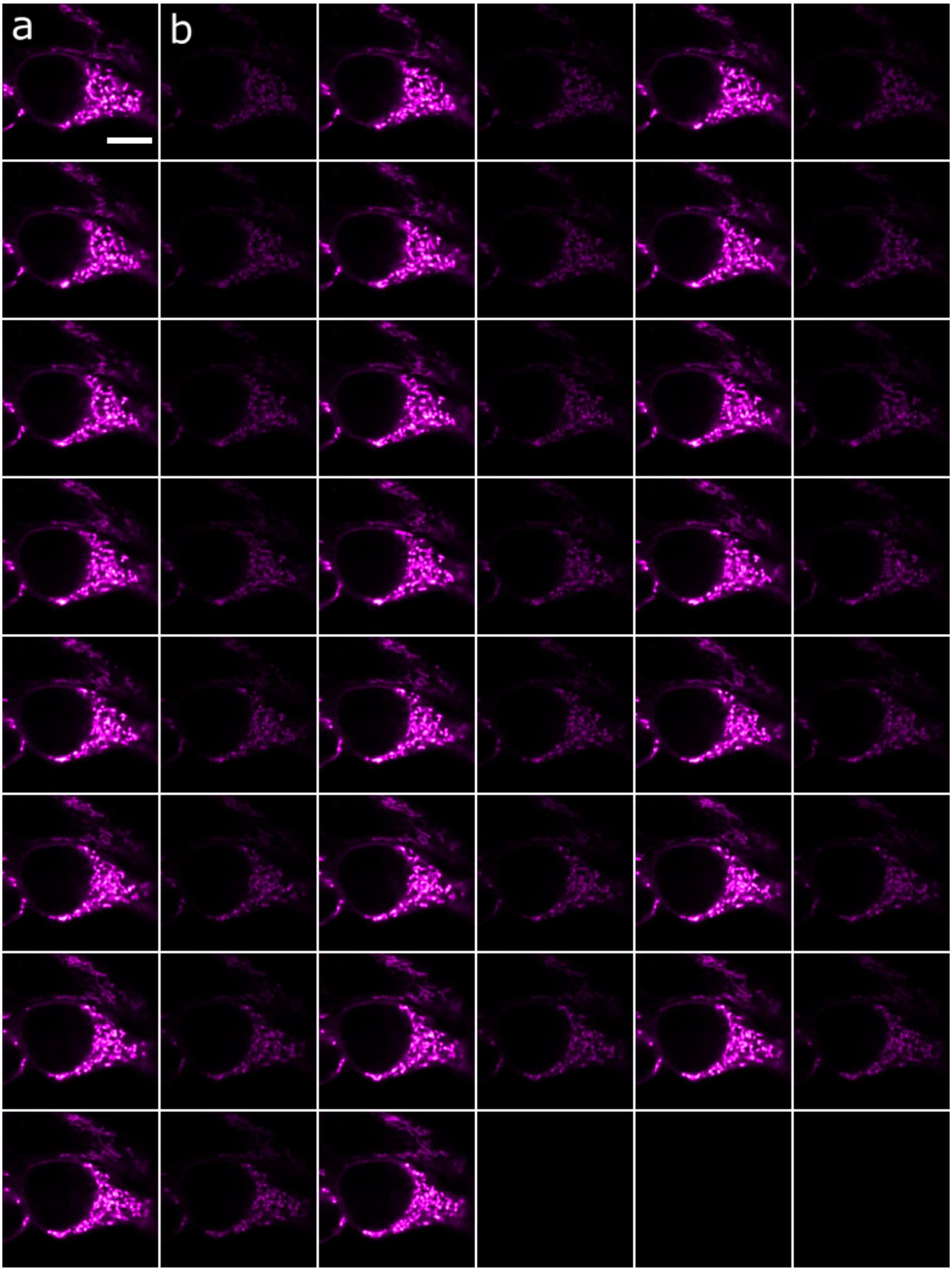

Figure S31. Confocal images of **Dyad 1** in live HEK cells. a) Bright state by excitation at 561 nm. b) Dark state by irradiation at 405 nm immediately followed by excitation at 561 nm. Switching cycles were repeated as in a) and b). Scale bar, 10 μm.

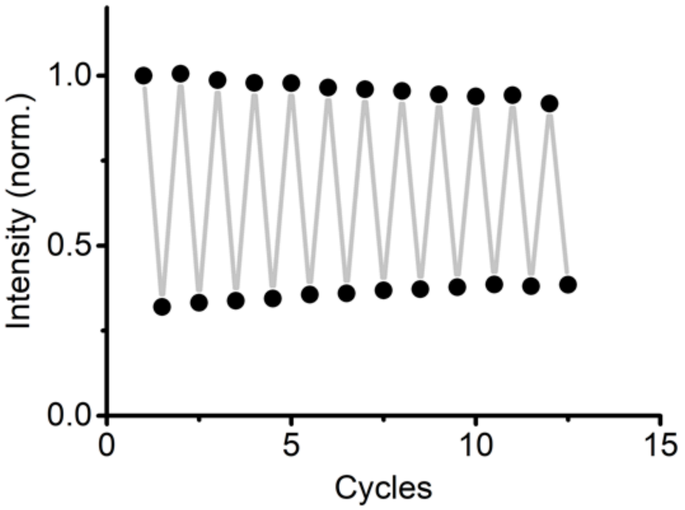

Figure S32. Fatigue resistance of **Dyad 2** in live HEK cells under a confocal microscope, using fluorescence quantification of the images in Figure S33.

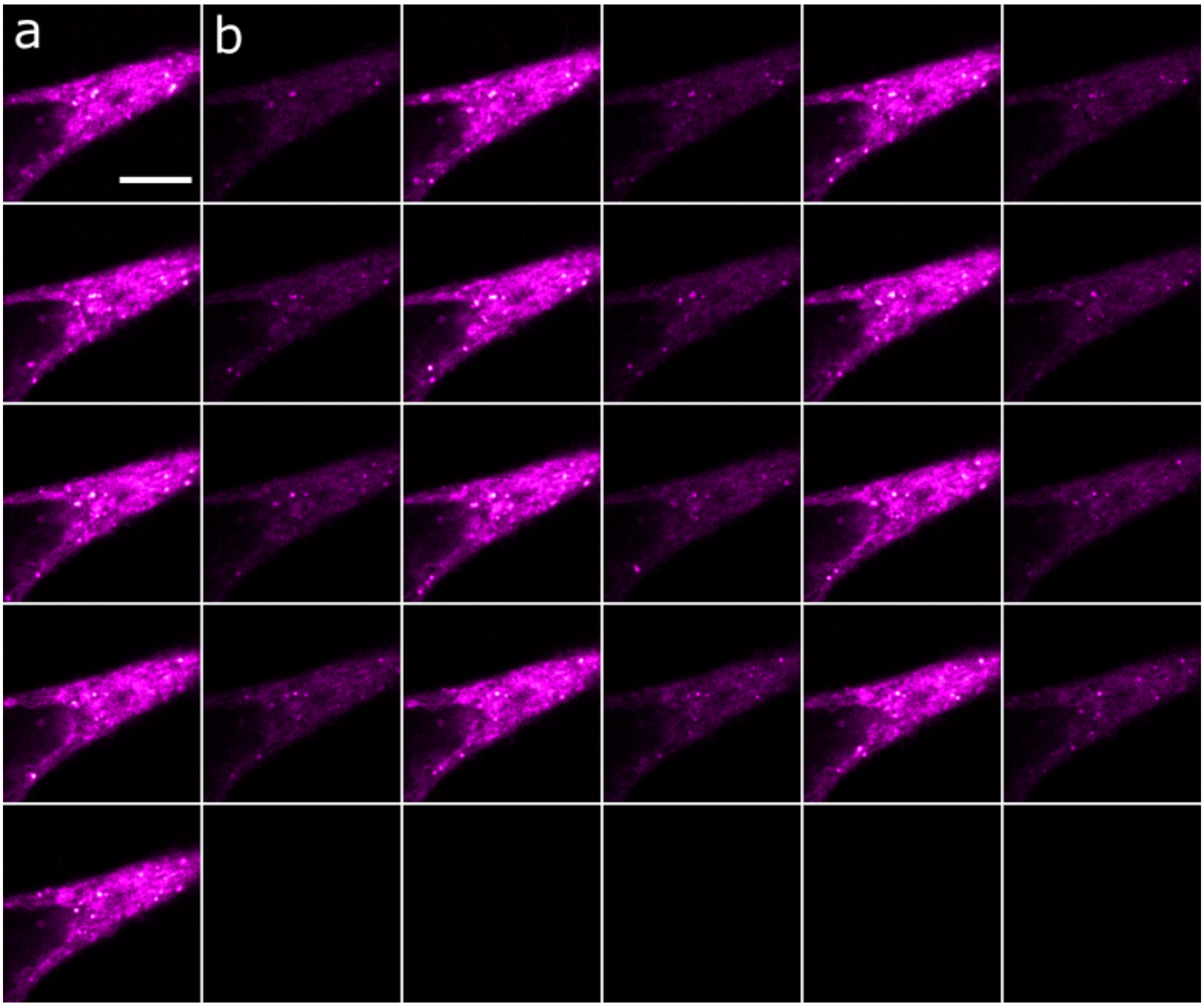

Figure S33. Confocal images of **Dyad 2** in live HEK cells. a) Bright state by excitation at 561 nm. b) Dark state by irradiation at 405 nm immediately followed by excitation at 561 nm. Switching cycles were repeated as in a) and b). Scale bar, 10 μm.

S17. Experimental synthetic procedures

1,3,3-Trimethyl-2-methyleneindoline-5-carboxylic acid[23] was prepared according to the reported procedure.

***tert*-Butyl 2,3,3-trimethyl-3*H*-indole-5-carboxylate (2)**

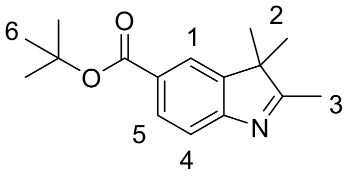

tert-Butyl 2,2,2-trichloroacetimidate (2.20 g, 10 mmol) was dried in a flask under high vacuum and subsequently dissolved in cyclohexane (10 mL). Meanwhile, 2,3,3-trimethyl-3*H*-indole-5-carboxylic acid (**1**) (1.00 g, 4.9 mmol) was dried and dissolved in THF (5 mL). The first solution was added dropwise to the second solution under nitrogen. $BF_3 \cdot OEt_2$ (0.18 mL, 1.6 mmol) was added and the mixture was stirred for 20 min at room temperature. After stirring, the mixture was diluted with 5% $NaHCO_3$ (aq., 200 mL) and the product was extracted with EtOAc (200 mL). The organic phase was washed with brine, dried using Na_2SO_4 and the residue purified by silica gel chromatography (95:5 to 8:2 40–60 °C petroleum ether/EtOAc), yielding the product as a white solid (817 mg, 64%). ^1H NMR (400 MHz, $CDCl_3$) δ (ppm): 7.98 (dd, *J* = 8.1, 1.7 Hz, 1H, H5), 7.91 (dd, *J* = 1.7, 0.6 Hz, 1H, H1), 7.53 (d, *J* = 8.1 Hz, 1H, H4), 2.31 (s, 3H, H3), 1.60 (s, 9H, H6), 1.33 (s, 6H, H2). ^{13}C {^1H} NMR (101 MHz, $CDCl_3$) δ (ppm): 191.4, 166.1, 157.3, 145.6, 129.9, 128.8, 122.5, 119.4, 81.0, 53.8, 28.3, 23.0, 15.7.

5-(*tert*-Butoxycarbonyl)-1,2,3,3-tetramethyl-3*H*-indol-1-ium iodide (3)

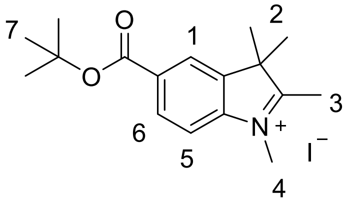

tert-Butyl 2,3,3-trimethyl-3*H*-indole-5-carboxylate (**2**) (817 mg, 3.15 mmol) was dissolved in 1,2-dichlorobenzene (12 mL) and CH_3I was added (3.92 mL, 63.1 mmol). The tube was sealed and heated to 80 °C for 24 h. After cooling to room temperature, the mixture was washed with cold Et_2O and filtered, yielding the product as a beige solid (978 mg, 77%). R_f (SiO_2, 9:1 CH_2Cl_2/CH_3OH) = 0.7. ^1H NMR (400 MHz, $CDCl_3$) δ (ppm): 8.21 (d, *J* = 8.4 Hz, 1H, H6), 8.11 (s, 1H, H1), 7.76 (d, *J* = 8.4 Hz, 1H, H5), 4.31 (s, 3H, H4), 3.13 (s, 3H, H3), 1.70 (s, 6H, H2), 1.62 (s, 9H, H7). ^{13}C{^1H} (101 MHz, $CDCl_3$) δ (ppm): 164.1, 144.7, 141.4, 134.0, 131.3, 124.2, 115.3, 82.8, 55.0, 38.0, 28.3, 23.3, 17.9. HRMS (ESI +ve) *m/z*: 274.18004, ([M-I]$^+$, $C_{17}H_{24}O_2N$ requires 274.18016).

***tert*-Butyl 1,3,3-trimethyl-2-methyleneindoline-5-carboxylate (4)**

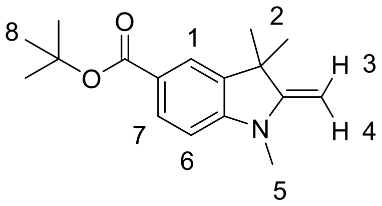

5-(*tert*-Butoxycarbonyl)-1,2,3,3-tetramethyl-3*H*-indol-1-ium iodide (**3**) (190 mg, 0.49 mmol) was dissolved in aqueous KOH solution (0.3 M, 4.9 mL) and isopropyl alcohol (5.0 mL). After stirring at room temperature for 2 h, the mixture was concentrated *in vacuo* and extracted with CH_2Cl_2. Evaporation of the solvent afforded a white solid (132 mg, 99%). The product turns pink in the presence of light. ^1H NMR (400 MHz, $CDCl_3$) δ (ppm): 7.83 (dd, *J* = 8.3, 1.8 Hz, 1H, H7), 7.68 (d, *J* = 1.7 Hz, 1H, H1), 6.50 (d, *J* = 8.3 Hz, 1H, H6), 3.95 (2nd order m, 2H, H3, H4), 3.07 (s, 3H, H5), 1.58 (s, 9H, H8), 1.35 (s, 6H, H2, H2'). ^{13}C{^1H} (101 MHz, $CDCl_3$) δ (ppm): 166.3, 162.3, 149.9, 137.3, 130.7, 123.0, 122.0, 104.0, 80.0, 75.6, 43.7, 29.8, 28.8, 28.3.

tert-Butyl 6'-(indolin-1-yl)-1,3,3-trimethylspiro[indoline-2,3'-naphtho[2,1-_b_][1,4]oxazine]-5-carboxylate (SO)

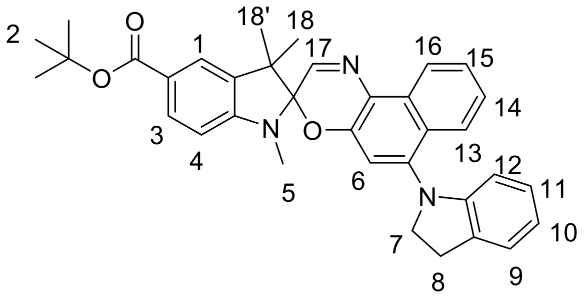

Zinc salt of 1-nitrosonaphthalen-2-olate (**5**) was prepared by following the reported procedure.[1] The zinc complex (500 mg, 1.22 mmol) was dissolved in EtOH (25 mL) and indoline (**6**) (0.48 mL, 4.3 mmol) added. The mixture was heated at 78 °C for 3 h under aerobic conditions. *tert*-Butyl 1,3,3-trimethyl-2-methyleneindoline-5-carboxylate (**4**) (534 mg, 1.95 mmol) in EtOH (28 mL) and Na_2SO_4 (800 mg) were added. The system was purged with argon and left to be heated at 78 °C for 21 h. The mixture was cooled to room temperature, and the precipitates were filtered, and washed with cold acetone (5 mL). The residue was purified by silica gel chromatography (3:7 to 7:3 CH_2Cl_2/40–60 °C petroleum ether) to afford the product as a pale yellow solid (203 mg, 22%). R_f (SiO_2, CH_2Cl_2) = 0.4. ^1H NMR (400 MHz, $CDCl_3$) δ (ppm): 8.61 (d, J = 8.6 Hz, 1H, H16), 7.96 (d, J = 8.3 Hz, 1H, H13), 7.92 (dd, J = 8.4, 1.7 Hz, 1H, H3), 7.69 (d, J = 1.7 Hz, 1H, H1), 7.67 (s, 1H, H17), 7.60 (dd, J = 8.4, 6.8 Hz, 1H, H15), 7.35 (dd, J = 8.4, 6.8 Hz, 1H, H14), 7.20 (d, J = 6.7 Hz, 1H, H9), 6.94 (dd, J = 7.4, 7.4 Hz, 1H, H11), 6.91 (s, 1H, H6), 6.74 (dd, J = 7.4, 7.4 Hz, 1H, H10), 6.53 (d, J = 8.2 Hz, 1H, H4), 6.30 (d, J = 7.8 Hz, 1H, H12), 3.93 (br. s, 2H, H7), 3.18 (br. s, 2H, H8), 2.83 (s, 3H, H5), 1.58 (s, 9H, H2), 1.40–1.36 (2 x s overlapped, 6 H, H18, H18'). $^{13}C\{^1H\}$ (101 MHz, $CDCl_3$) δ (ppm): 166.2, 151.3, 150.0, 148.6, 144.6, 144.4, 135.9, 131.8, 131.0, 127.7, 127.0, 125.50, 125.48, 124.8, 123.9, 123.6, 123.1, 122.0, 120.6, 119.3, 110.0, 108.6, 106.2, 98.9, 80.4, 55.3, 51.5, 29.7, 29.0, 28.5, 25.5, 20.9. HRMS (ESI +ve) *m/z*: 546.27533, ($[M+H]^+$, $C_{35}H_{36}O_3N_3$ requires 546.27512). UV-vis (CH_3CN) λ / nm (ε / M^{-1} cm^{-1}) 291 (2.8 × 10^4), 387 (1.3 × 10^4); UV-vis (CH_2Cl_2) λ / nm (ε / M^{-1} cm^{-1}) 292 (2.5 × 10^4), 390 (1.4 × 10^4); UV-vis (toluene) λ / nm (ε / M^{-1} cm^{-1}) 391 (1.4 × 10^4); UV-vis (cyclohexane) λ / nm (ε / M^{-1} cm^{-1}) 279 (3.3 × 10^4), 387 (1.5 × 10^4).

*Synthesis by de-protection of switch **SO**:*

6'-(Indolin-1-yl)-1,3,3-trimethylspiro[indoline-2,3'-naphtho[2,1-b][1,4]oxazine]-5-carboxylic acid (7)

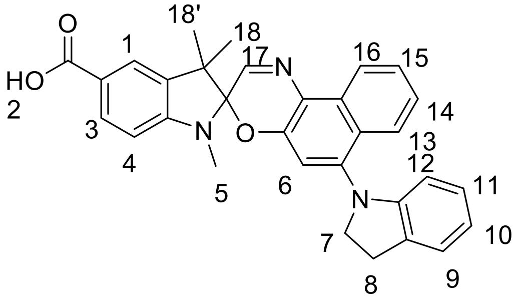

tert-Butyl 6'-(indolin-1-yl)-1,3,3-trimethylspiro[indoline-2,3'-naphtho[2,1-*b*][1,4]oxazine]-5-carboxylate (**SO**) (94 mg, 0.17 mmol) was dried with silica gel (1.0 g) under high vacuum at room temperature. Dry toluene (20 mL) was added and the resulting mixture was heated under reflux overnight. The silica was filtered off and the solvent was removed *in vacuo*. The residue was purified by silica gel chromatography (CH_2Cl_2 to 9:1 CH_2Cl_2/CH_3OH) to yield the product as a pale yellow solid (65 mg, 77%). R_f(SiO_2, 9:1 CH_2Cl_2/CH_3OH) = 0.5. ^1H NMR (400 MHz, 1:1 CD_3OD/$CDCl_3$) δ (ppm): 8.54 (dt, J = 8.5, 0.9 Hz, 1H, H16), 7.98 – 7.89 (overlapped, 2H, H3, H13), 7.72 (d, J = 1.6 Hz, 1H, H1), 7.67 (s, 1H, H17), 7.62 – 7.56 (m overlapped with solvent, 1H, H15), 7.34 (ddd, J = 8.3, 6.8, 1.3 Hz, 1H, H14), 7.16 (dd, J = 7.3, 1.3 Hz, 1H, H9), 6.92 (s, 1H, H6), 6.89 (ddd, J = 7.8, 7.8, 1.3 Hz, 1H, H11), 6.70 (ddd, J = 7.4, 7.4, 1.0 Hz, 1H, H10), 6.58 (d, J = 8.3 Hz, 1H, H4), 6.25 (d, J = 7.9 Hz, 1H, H12), 3.91 (br. s, 2H, H7), 3.15 (br. s, 2H, H8), 2.84 (s, 3H, H5), 1.37 (s, 3H, H18), 1.36 (s, 3H, H18'). $^{13}C\{^1H\}$ NMR (101 MHz, 1:1 CD_3OD/$CDCl_3$) δ (ppm): 151.5, 149.8, 148.7, 144.3, 135.8, 131.6, 131.5, 130.9, 127.5, 127.5, 126.7, 125.5, 124.6, 124.5, 123.6, 123.3, 121.6, 120.4, 119.1, 109.7, 108.4, 106.1, 98.7, 55.2, 51.2, 29.1, 28.7, 25.0, 20.5. HRMS (ESI +ve) *m/z*: 490.21262 ($[M+H]^+$, $C_{31}H_{28}O_3N_3$ requires 490.21252). UV-vis (cyclohexane) of the closed form λ / nm (ε / M^{-1} cm^{-1}) 281 (4.6 × 10^4), 388 (2.0 × 10^4); UV-vis (cyclohexane) of the PSS λ / nm (ε / M^{-1} cm^{-1}) 560 (8.0 × 10^4).

Synthesis using one-pot reaction involving a zinc-chelate complex[1]

6'-(Indolin-1-yl)-1,3,3-trimethylspiro[indoline-2,3'-naphtho[2,1-*b*][1,4]oxazine]-5-carboxylic acid (7)

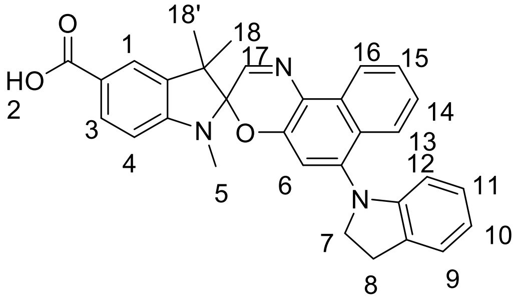

Zinc salt of 1-nitrosonaphthalen-2-olate (**5**) (1.45 g, 3.53 mmol) was dissolved in EtOH (74 mL) and indoline (**6**) (1.38 mL, 12.4 mmol) added. The mixture was heated at 78 °C for 3 h under aerobic conditions. 1,3,3-Trimethyl-2-methyleneindoline-5-carboxylic acid (**S1**) (1.15 g, 5.29 mmol) in EtOH (74 mL) and Na_2SO_4 (4.6 g) were added. The system was purged with argon and left to be heated at 78 °C for 27 h. The mixture was cooled to room temperature, and Na_2SO_4 was removed by filtration. After solvent removal of the filtrate, the crude was purified by silica gel chromatography (CH_2Cl_2, 99.5:0.5 to 9:1 CH_2Cl_2/CH_3OH), and recrystallized from CH_2Cl_2 and then CH_3OH several times to get a pale yellow solid (737 mg, 28%). Characterization data match those given above.

3-Carboxy-4-(1,11-diethyl-3,4,8,9,10,11-hexahydro-2*H*-pyrano[3,2-*g*:5,6-*g'*]diquinolin-1-ium-6-yl)benzoate, chloride salt (5'-Atto 565, x) and **4-carboxy-3-(1,11-diethyl-3,4,8,9,10,11-hexahydro-2*H*-pyrano[3,2-*g*:5,6-*g'*]diquinolin-1-ium-6-yl)benzoate (6'-Atto 565), chloride salt**[3]

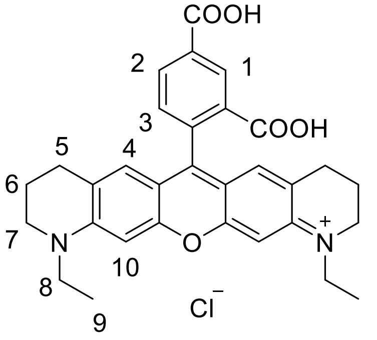

5'-Atto 565

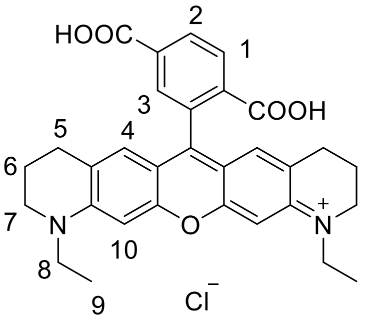

6'-Atto 565

1-Ethyl-1,2,3,4-tetrahydroquinolin-7-ol (**SI2**) (2.32 g, 13.1 mmol) and trimellitic anhydride (1.26 g, 6.54 mmol) were dissolved in butyric acid (100 mL), and conc. H_2SO_4 (50 µL) was added. The mixture was heated to 164 °C for 28 h, and the solvents removed by vacuum distillation to generate a dark purple gum. Separation of the regioisomers was achieved by silica gel chromatography (1.5:5:93.5 to 3:15:82 NEt_3/CH_3OH/CH_2Cl_2). Each of the resulting bands was dissolved in 10% ^{i}PrOH/$CHCl_3$, washed with 3 M aqueous HCl solution and H_2O, dried over Na_2SO_4, and the solvents were removed *in vacuo*. The solids were further purified by recrystallization from warm $CHCl_3$.

5'-Atto 565, 706 mg, yield: 20%.

R_f(SiO_2, 3:10:87 NEt_3/CH_3OH/CH_2Cl_2) = 0.15. ^{1}H NMR (400 MHz, CD_3OD) δ (ppm): 8.91 (d, J = 1.6 Hz, 1H, H1), 8.42 (dd, J = 7.6, 1.6 Hz, 1H, H2), 7.49 (d, J = 7.6 Hz, 1H, H3), 6.93 (s, 2H, H4), 6.74 (s, 2H, H10), 3.67 (q, J = 7.1 Hz, 4H, H8), 3.58 (t, J = 5.6 Hz, 4H, H7), 2.69 (t, J = 6.2 Hz, 4H, H5), 1.99–1.89 (m, 4H, H6), 1.33 (t, J = 7.2 Hz, 6H, H9). UV-vis (PBS) λ / nm (ε / M^{-1} cm^{-1}) 564 (1.8 × 10^5). Fluorescence emission (PBS) λ / nm (ϕ_f): 589 (80%). UV-vis (CH_3OH) λ / nm (ε / M^{-1} cm^{-1}) 554 (1.7 × 10^5). Fluorescence emission (CH_3OH) λ / nm (ϕ_f): 578 (85%). UV-vis (H_2O) λ / nm (ε / M^{-1} cm^{-1}) 563 (1.6 × 10^5). Fluorescence emission (H_2O) λ / nm (ϕ_f): 589 (83%). HRMS (ESI +ve) m/z: 511.22287 ([M-Cl]$^+$, $C_{31}H_{31}O_5N_2$ requires 511.22275).

6'- Atto 565, 1.00 g, yield: 28%.

R_f (SiO_2, 3:10:87 $NEt_3/CH_3OH/CH_2Cl_2$) = 0.25. ^1H NMR (400 MHz, CD_3OD) δ (ppm): 8.44–8.37 (2nd order, 12H, H1, H2), 7.93 (d, J = 0.9 Hz, 1H, H3), 6.93 (s, 2H, H4), 6.75 (s, 2H, H10), 3.67 (q, J = 7.1 Hz, 4H, H8), 3.58 (t, J = 5.6 Hz, 4H, H7), 2.70 (t, J = 6.2 Hz, 4H, H5), 1.99–1.89 (m, 4H, H6), 1.34 (t, J = 7.2 Hz, 6H, H9). UV-vis (H_2O) λ / nm (ε / M^{-1} cm^{-1}) 562 (1.3×10^5). Fluorescence emission (H_2O) λ / nm (ϕ_f): 586 (83%). HRMS (ESI +ve) m/z: 511.22260 ($[M-Cl]^+$, $C_{31}H_{31}O_5N_2$ requires 511.22275).

1,11-Diethyl-6-(4-((2-(6'-(indolin-1-yl)-1,3,3-trimethylspiro[indoline-2,3'-naphtho[2,1-*b*][1,4]oxazine]-5-carboxamido)ethyl)carbamoyl)-2-(methoxycarbonyl)phenyl)-3,4,8,9,10,11-hexahydro-2*H*-pyrano[3,2-*g*:5,6-*g'*]diquinolin-1-ium chloride (Dyad 1)

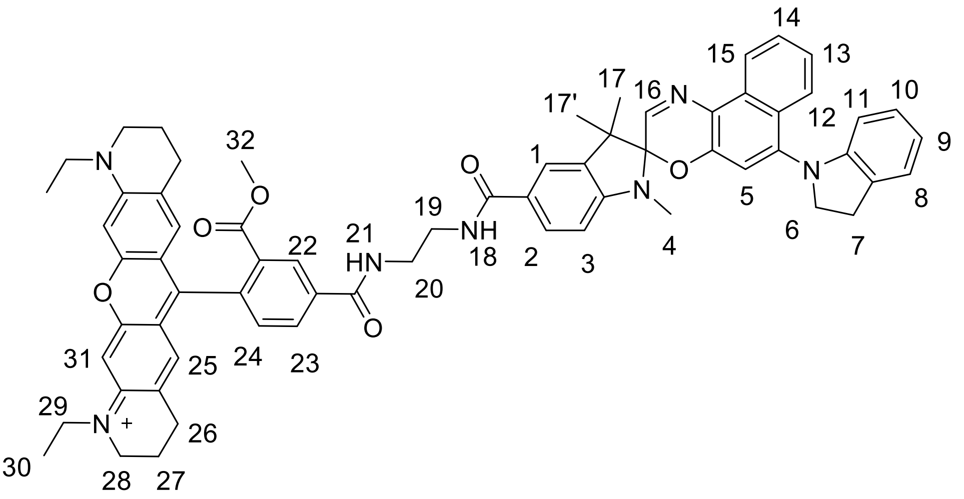

6-(2,4-Dicarboxyphenyl)-1,11-diethyl-3,4,8,9,10,11-hexahydro-2*H*-pyrano[3,2-*g*:5,6-*g'*]diquinolin-1-ium chloride (**5'-Atto565**) (12 mg, 23 μmol) was dissolved in dry DMF (2 mL) and HBTU (17 mg, 45 μmol) was added. After stirring for 2 min at room temperature, *N*-Boc-ethylenediamine (0.19 mL, 58 μmol) and triethylamine (40 μL, 0.29 mmol) were added. The mixture was left to stir for another 10 min and the reaction was monitored by TLC. After consumption of the starting material, the solvent was distilled off *in vacuo* using a short path distillation setup. The crude product was purified by silica gel chromatography (3:10:87 $NEt_3/CH_3OH/CH_2Cl_2$). The compound (16 mg) was slightly impure and consequently the yield was later reported over 3 steps.

The compound above (8.0 mg, 11 μmol) was stirred in a 1:1 mixture of CH_2Cl_2 and TFA for 2 h at room temperature. A crude NMR confirmed the Boc-deprotected compound. In the meanwhile, 6'-(indolin-1-yl)-1,3,3-trimethylspiro[indoline-2,3'-naphtho[2,1-*b*][1,4]oxazine]-5-carboxylic acid (**7**) (3.5 mg, 7.2 μmol) was dissolved in dry DMF (1 mL) and HBTU (6.3 mg, 17 μmol) was added. After 5 min of stirring, the amine solution was added with triethylamine (40 μL, 0.29 mmol). The reaction was monitored by TLC (3:17 CH_3OH/CH_2Cl_2). CH_3OH was added and after complete consumption of the starting material, the solvent was distilled off *in vacuo* using a distillation apparatus, and the crude product was purified by silica gel chromatography (3:10:87 $NEt_3/CH_3OH/CH_2Cl_2$). Further purification was carried out using semi-preparative HPLC (normal phase silica, gradient CH_2Cl_2/CH_3OH) to afford the product as a pink solid (1.2 mg, 16%). ^1H NMR (500 MHz, $CDCl_3$) δ (ppm): 8.73 (d, J = 1.8 Hz, 1H, H22), 8.53 (d, J = 8.4 Hz, 1H, H15), 8.19 (dd, J = 7.9, 1.8 Hz, 1H, H23), 7.87 (d, J = 8.5 Hz, 1H, H12), 7.82 (dd, J = 8.3, 1.8 Hz, 1H, H2), 7.65 (d, J = 1.8 Hz, 1H, H1), 7.60 (s, 1H, H16), 7.57 (br.s, 1H, H21), 7.54 – 7.49 (m, 1H, H14), 7.30 – 7.25 (m, 1H, H13), 7.23 (d, J = 7.8 Hz, 1H, H24), 7.18 (overlapped with solvent, 1H, H18), 7.10 (d, J = 7.1 Hz, 1H, H8), 6.83 (t and s overlapped, 2H, H5, H10), 6.65 (m and s overlapped, 3H, H9, H25), 6.60 (s, 2H, H31), 6.51 (d, J = 8.2 Hz, 1H, H3), 6.20 (d, J = 8.0 Hz, 1H, H11), 3.85 (br. s, 2H, H6), 3.79 – 3.70 (m, 5H, H19, H20), 3.64 (s, 3H, H32), 3.54 – 3.45 (m, 4H, H29), 3.45 – 3.49 (m, 5H, H28), 3.12 (br. s, 4H, H7), 2.72

(s, 3H, H4), 2.67 – 2.57 (m, 4H, H26), 1.91 – 1.83 (m, 4H, H27), 1.29 – 1.13(overlapped, 38 H, H17, H17', H30). LRMS (MALDI) *m/z*: 1038.85, ([M-anion]$^+$, $C_{65}H_{64}O_6N_7$ requires 1038.85). UV-vis (PBS) λ / nm (ε / M^{-1} cm^{-1}): 371 (4.5×10^4), 576 (1.4×10^5); UV-vis (CH_3OH) λ / nm (ε /M^{-1} cm^{-1}): 366 (3.8×10^4), 566 (2.2×10^5); UV-vis (CH_2Cl_2) λ / nm (ε / M^{-1} cm^{-1}): 368 (3.5×10^4), 567 (2.5×10^5).

6-(5-((2-((*tert*-Butoxycarbonyl)amino)ethyl)carbamoyl)-2-carboxyphenyl)-1,11-diethyl-3,4,8,9,10,11-hexahydro-2*H*-pyrano[3,2-g:5,6-*g'*]diquinolin-1-ium chloride (8)

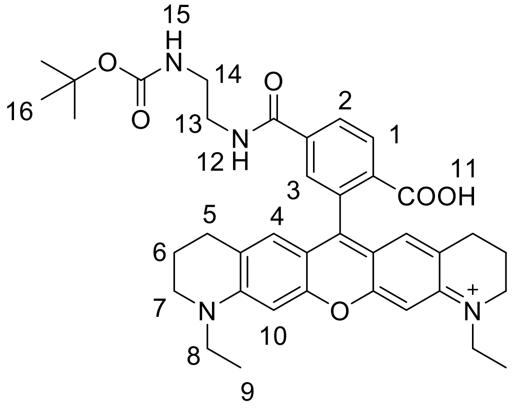

6-(2,5-Dicarboxyphenyl)-1,11-diethyl-3,4,8,9,10,11-hexahydro-2*H*-pyrano[3,2-g:5,6-*g'*]diquinolin-1-ium chloride (**6'-Atto565**) (130 mg, 0.24 mmol) was dissolved in dried DMF (5 mL), and triethylamine (83 µL, 0.6 mmol) and HBTU (99 mg, 0.26 mmol) were added After stirring for 2 min at room temperature, a DMF (5 mL) solution of *N*-Boc-ethylenediamine (38 µL, 0.24 mmol) and triethylamine (83 µL, 0.6 mmol) was added. The mixture was left to stir for 30 min. The reaction was quenched with H_2O (1 mL). The solvent was removed under strong N_2 flow at 40 °C. The crude product was purified by silica gel chromatography (1:9 CH_3OH/CH_2Cl_2) to yield a pink solid (82 mg, 32%). ^1H NMR (500 MHz, $CDCl_3$) δ (ppm): 8.19 (d, *J* = 8.1 Hz, 1H, H1), 7.96 (d, *J* = 8.0 Hz, 1H, H2), 7.72 (br. s, 1H, H15), 7.63 (s, 1H, H3), 6.66 (s, 2H, H4), 6.50 (s, 2H, H10), 5.26 (t, *J* = 6.0 Hz, 1H, H12), 3.55 – 3.27 (overlapped, 13H, H7, H8, H13, H14), 2.70 – 2.51 (m, 4H, H5), 1.96 – 1.81 (m, 4H, H6), 1.38 (s, 9H, H16), 1.25 (t, *J* = 7.0 Hz, 8H, H9). HRMS (ESI +ve) *m/z*: 653.33307 ([M-anion]$^+$, $C_{38}H_{45}O_6N_4$ requires 653.33336).

6-(2-Carboxy-4-((2-(6'-(indolin-1-yl)-1,3,3-trimethylspiro[indoline-2,3'-naphtho[2,1-*b*][1,4]oxazine]-5-carboxamido)ethyl)carbamoyl)phenyl)-1,11-diethyl-3,4,8,9,10,11-hexahydro-2*H*-pyrano[3,2-*g*:5,6-*g'*]diquinolin-1-ium (Dayd 2)

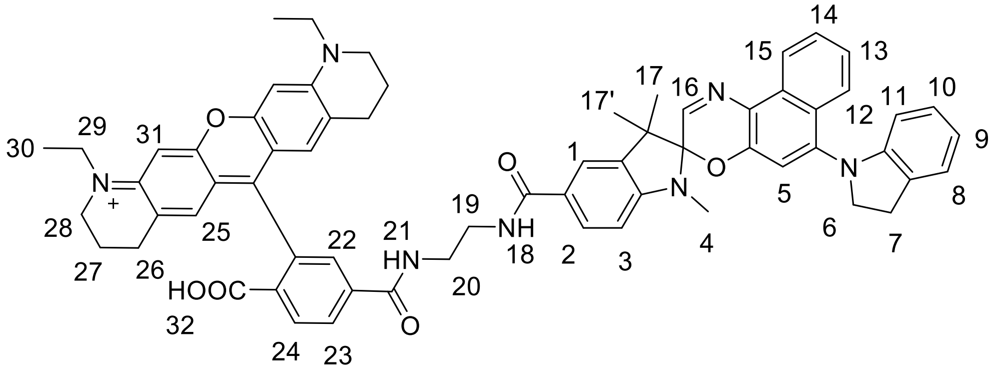

6-(5-((2-((*tert*-Butoxycarbonyl)amino)ethyl)carbamoyl)-2-carboxyphenyl)-1,11-diethyl-3,4,8,9,10,11-hexahydro-2*H*-pyrano[3,2-*g*:5,6-*g'*]diquinolin-1-ium chloride (**8**) (7.7 mg, 14 µmol) was stirred in a 1:1 mixture of CH_2Cl_2 and TFA for 2 h at room temperature. A crude NMR confirmed the Boc-deprotected compound. At the same time, 6'-(indolin-1-yl)-1,3,3-trimethylspiro[indoline-2,3'-naphtho[2,1-*b*][1,4]oxazine]-5-carboxylic acid (**7**) (5.0 mg, 10 µmol) was dissolved in dry DMF (2 mL), DIPEA (2.7 µL, 15 µmol) and HBTU (3.9 mg, 10 µmol) was added. After 5 min of stirring, the amine solution was added with DIPEA (2.7 µL, 15 µmol). The reaction was left to stir for 30 min before being quenched with H_2O (1 mL). The solvent was removed by strong N_2 at 40 °C. The crude product was purified by silica gel chromatography (1:10:89 $CH_3COOH/CH_3OH/CH_2Cl_2$) to yield a pink solid (5.3 mg, 51%). ^1H NMR (500 MHz, $CDCl_3$) δ (ppm): 8.53 (br.s and d overlapped, *J* = 8.3 Hz, 2H, H15, NH or CO_2H), 8.23 (s, 1H, NH or CO_2H), 8.02 (d, *J* = 7.7 Hz, 1H, H24), 7.93 – 7.86 (overlapped, 2H, H12, H23), 7.72 (d, *J* = 8.3 Hz, 1H, H2), 7.68 (s, 1H, H22), 7.59 (s, 1H, H1), 7.57 – 7.50 (s and ddd overlapped, *J* = 8.3, 7.0, 1.2 Hz, 2H, H14,

H16), 7.28 (ddd, *J* = 8.3, 6.8, 1.3 Hz, 1H, H13), 7.12 (d, *J* = 7.3 Hz, 1H, H8), 6.89 – 6.83 (m, 1H, H10), 6.82 – 6.77 (overlapped s, 3H, H5, H25), 6.66 (ddd, *J* = 7.4, 7.4, 1.0, 1H, H9), 6.47 (d, *J* = 1.9 Hz, 2H, H31), 6.35 (d, *J* = 8.1 Hz, 1H, H3), 6.21 (d, *J* = 7.9 Hz, 1H, H11), 3.85 (br. s, 2H, H6), 3.57 – 3.44 (m, 4H, H19, H20), 3.38 (q, *J* = 7.2 Hz, 3H, H29), 3.29 (t, *J* = 5.1 Hz, 2H, H28), 3.10 (br. s, 2H, H7), 2.68 (s, 3H, H4), 2.60 – 2.42 (m, 4H, H26), 1.86 – 1.66 (m, 4H, H27), 1.25 – 1.14 (overlapped, 25H, H17, H17', H30). HRMS (ESI +ve) *m/z*: 1024.47485 ([M-ion]$^+$, $C_{64}H_{62}O_6N_7$ requires 1024.47561).

S18. References

[1] M.-L. Pang, H.-J. Zhang, P.-P. Liu, Z.-H. Zou, J. Han, J.-B. Meng, *Synthesis* **2010**, *20*, 3418–3422.

[2] M. J. Uddin, L. J. Marnett, *Org. Lett.* **2008**, *10*, 4799–4801.

[3] "ATTO-TEC GmbH - ATTO 565," can be found under https://www.atto-tec.com/attotecshop/product_info.php?language=en&info=p104_atto-565.html&, **n.d.**

[4] Y. Xiong, P. Rivera-Fuentes, E. Sezgin, A. Vargas Jentzsch, C. Eggeling, H. L. Anderson, *Org. Lett.* **2016**, *18*, 3666–3669.

[5] "Solvent Center - Let us help you find just the right solvent for your specific need.," can be found under https://www.sigmaaldrich.com/chemistry/solvents.html, **n.d.**

[6] J. L. Dean, *Lange's Handbook of Chemistry*, McGraw Hill, **1999**.

[7] C. Özçoban, T. Halbritter, S. Steinwand, L.-M. Herzig, J. Kohl-Landgraf, N. Askari, F. Groher, B. Fürtig, C. Richter, H. Schwalbe, et al., *Org. Lett.* **2015**, *17*, 1517–1520.

[8] M. Sobieraj, K. A. Krzyśko, A. Jarmuła, M. W. Kalinowski, B. Lesyng, M. Prokopowicz, J. Cieśla, A. Gojdź, B. Kierdaszuk, *J. Mol. Model.* **2015**, *21*, 1–14.

[9] A. Khadria, Y. de Coene, P. Gawel, C. Roche, K. Clays, H. L. Anderson, *Org. Biomol. Chem.* **2017**, *15*, 947–956.

[10] M. J. Frisch, G. W. Trucks, H. B. Schlegel, G. E. Scuseria, M. A. Robb, J. R. Cheeseman, G. Scalmani, V. Barone, G. A. Petersson, H. Nakatsuji, et al., *Gaussian 16*, Wallingford, CT, **2016**.

[11] J. Tomasi, B. Mennucci, R. Cammi, *Chem. Rev.* **2005**, *105*, 2999–3094.

[12] A. Perrier, F. Maurel, E. A. Perpète, V. Wathelet, D. Jacquemin, *J. Phys. Chem. A* **2009**, *113*, 13004–13012.

[13] G. M. Greetham, D. Sole, I. P. Clark, A. W. Parker, M. R. Pollard, M. Towrie, *Rev. Sci. Instrum.* **2012**, *83*, 103107-1 to 5.

[14] G. M. Greetham, P. M. Donaldson, C. Nation, I. V. Sazanovich, I. P. Clark, D. J. Shaw, A. W. Parker, M. Towrie, *Appl. Spectrosc.* **2016**, *70*, 645–653.

[15] G. M. Greetham, P. Burgos, Q. Cao, I. P. Clark, P. S. Codd, R. C. Farrow, M. W. George, M. Kogimtzis, P. Matousek, A. W. Parker, et al., *Appl. Spectrosc.* **2010**, *64*, 1311–1319.

[16] I. H. M. van Stokkum, D. S. Larsen, R. van Grondelle, *Biochim. Biophys. Acta BBA - Bioenerg.* **2004**, *1657*, 82–104.

[17] L. Palatinus, A. van der Lee, *J. Appl. Crystallogr.* **2008**, *41*, 975–984.

[18] L. Palatinus, *Acta Crystallogr. Sect. B Struct. Sci. Cryst. Eng. Mater.* **2013**, *69*, 1–16.

[19] L. Palatinus, G. Chapuis, *J. Appl. Crystallogr.* **2007**, *40*, 786–790.

[20] P. W. Betteridge, J. R. Carruthers, R. I. Cooper, K. Prout, D. J. Watkin, *J. Appl. Crystallogr.* **2003**, *36*, 1487–1487.

[21] R. I. Cooper, A. L. Thompson, D. J. Watkin, *J. Appl. Crystallogr.* **2010**, *43*, 1100–1107.

[22] P. Parois, R. I. Cooper, A. L. Thompson, *Chem. Cent. J.* **2015**, *9*, 30–43.

[23] S. Wan, Y. Zheng, J. Shen, W. Yang, M. Yin, *ACS Appl. Mater. Interfaces* **2014**, *6*, 19515–19519.

S19. Supporting Spectra

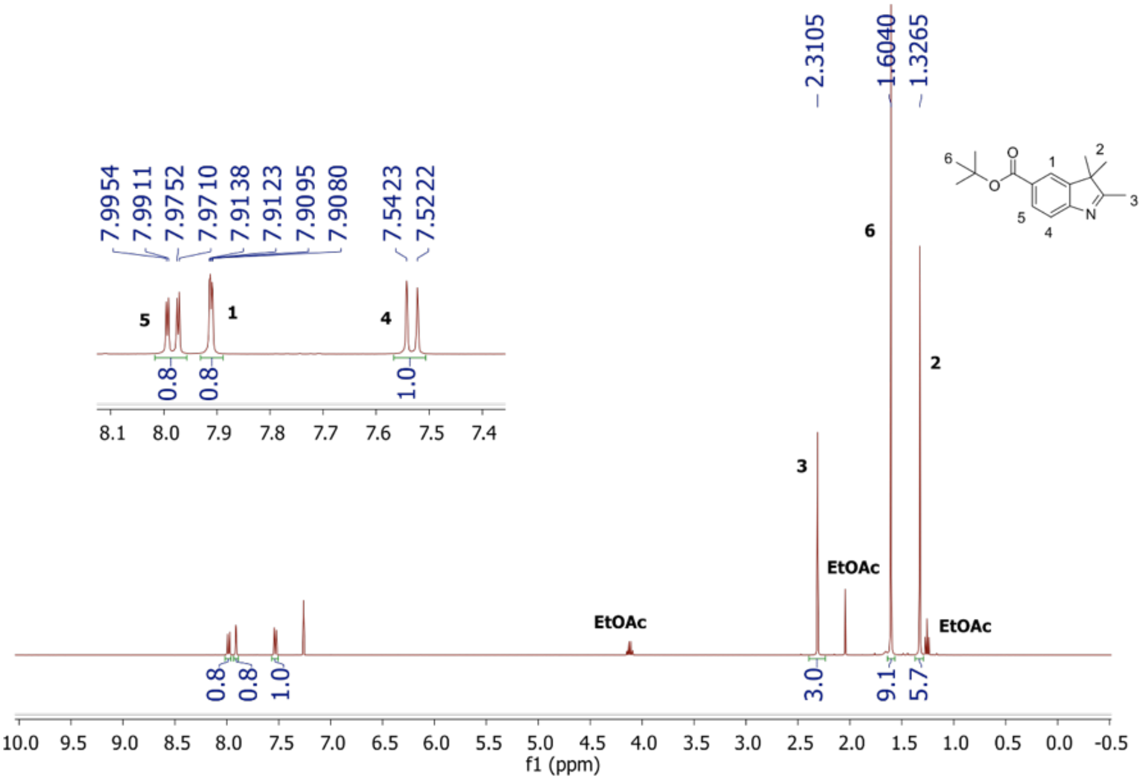

^{1}H ($CDCl_3$, 400 MHz) spectrum of *tert*-butyl 2,3,3-trimethyl-3*H*-indole-5-carboxylate (**2**).

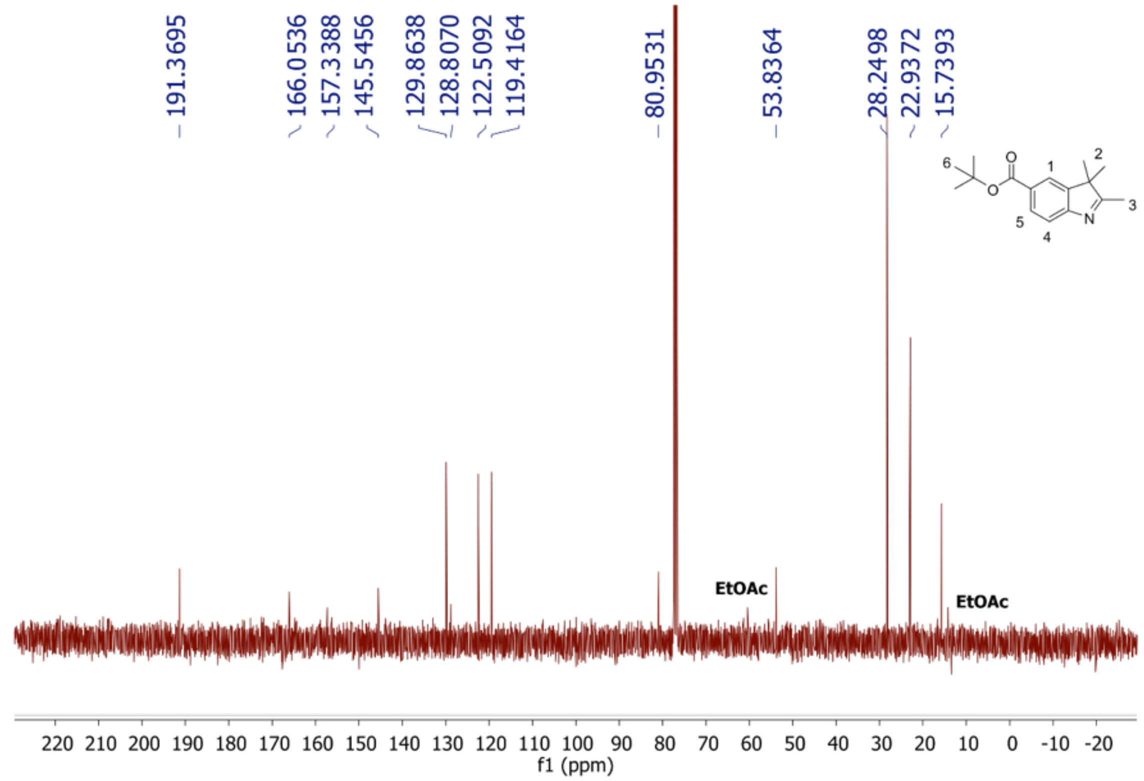

^{13}C{^{1}H} ($CDCl_3$, 101 MHz) spectrum of *tert*-butyl 2,3,3-trimethyl-3*H*-indole-5-carboxylate (**2**).

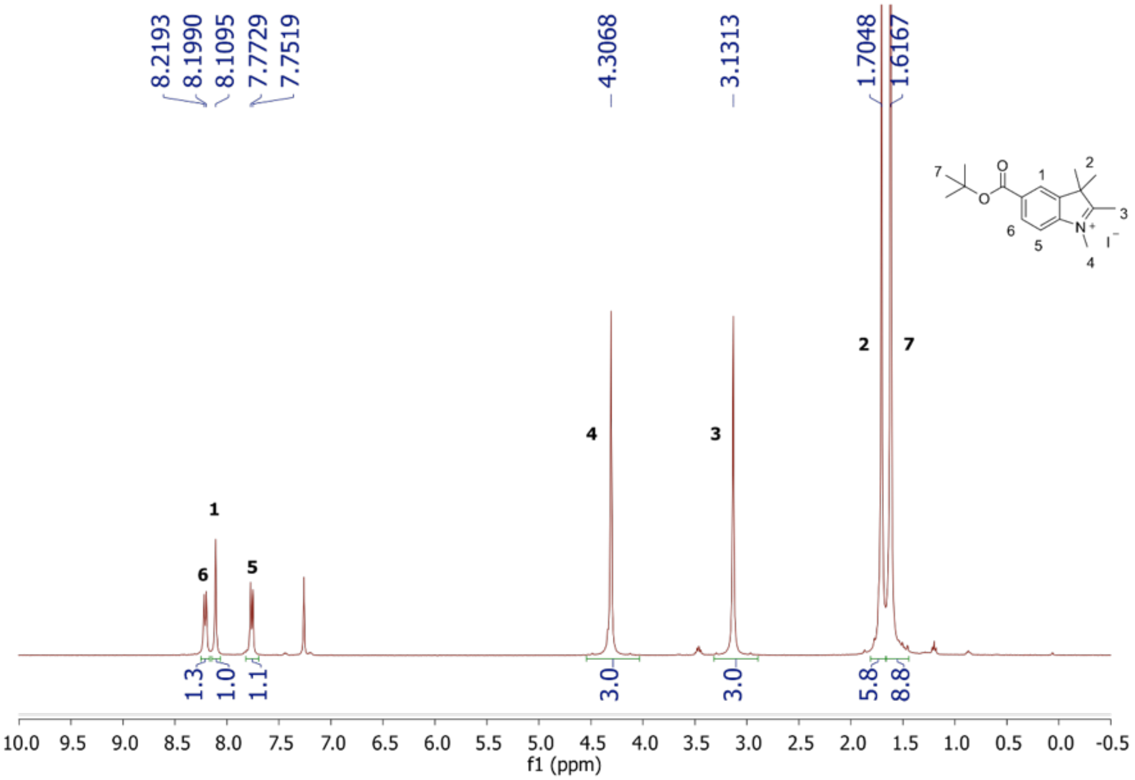

^{1}H ($CDCl_3$, 400 MHz) spectrum of 5-(*tert*-butoxycarbonyl)-1,2,3,3-tetramethyl-3*H*-indol-1-ium iodide (**3**).

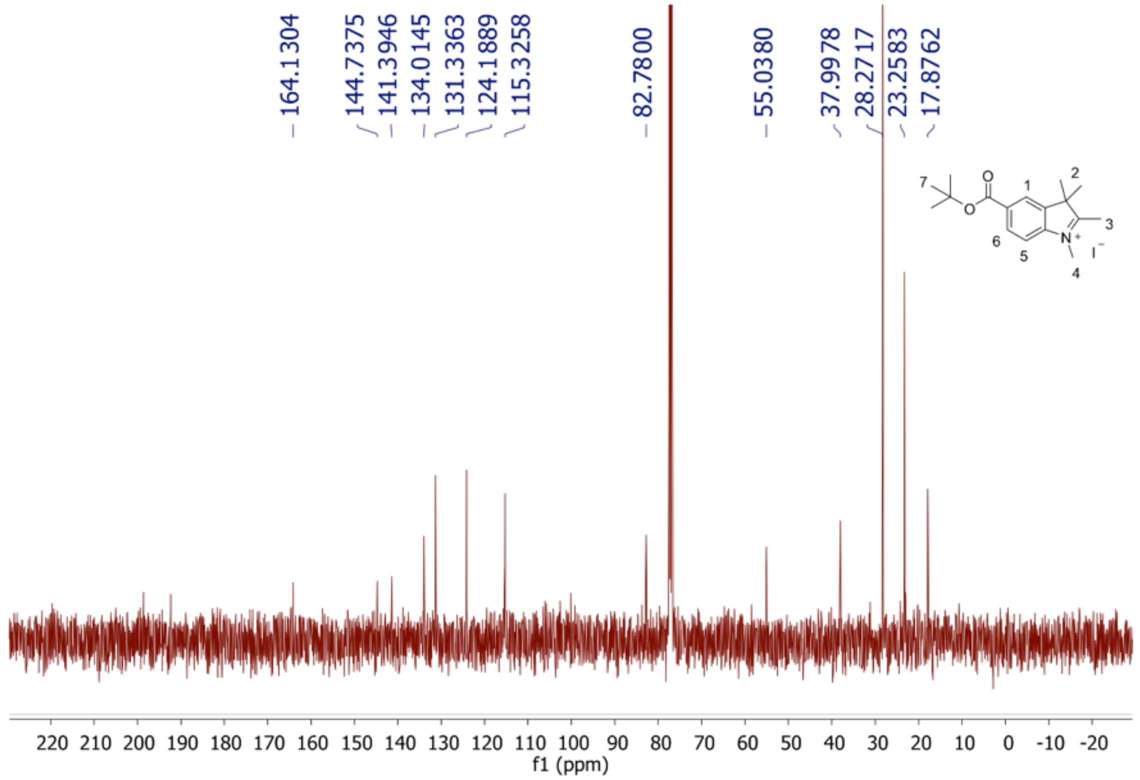

$^{13}C\{^{1}H\}$ ($CDCl_3$, 101 MHz) spectrum of 5-(*tert*-butoxycarbonyl)-1,2,3,3-tetramethyl-3*H*-indol-1-ium iodide (**3**).

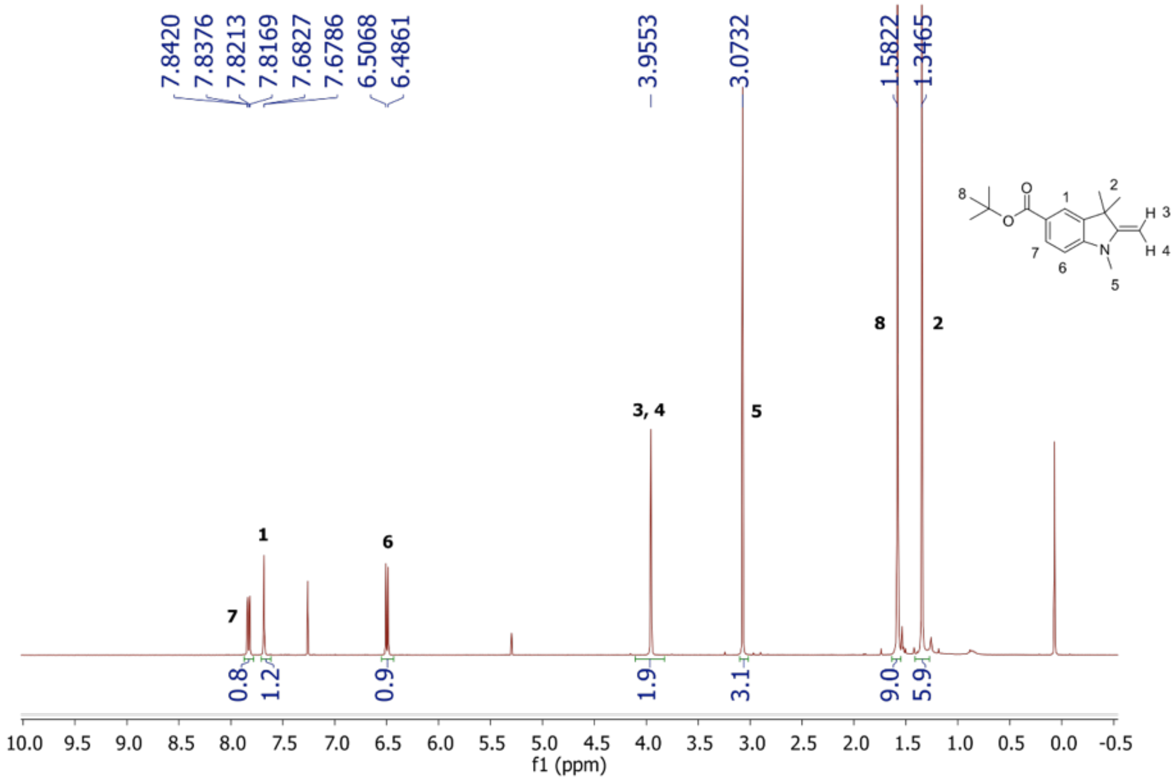

^{1}H ($CDCl_3$, 400 MHz) spectrum of *tert*-butyl 1,3,3-trimethyl-2-methyleneindoline-5-carboxylate (**4**).

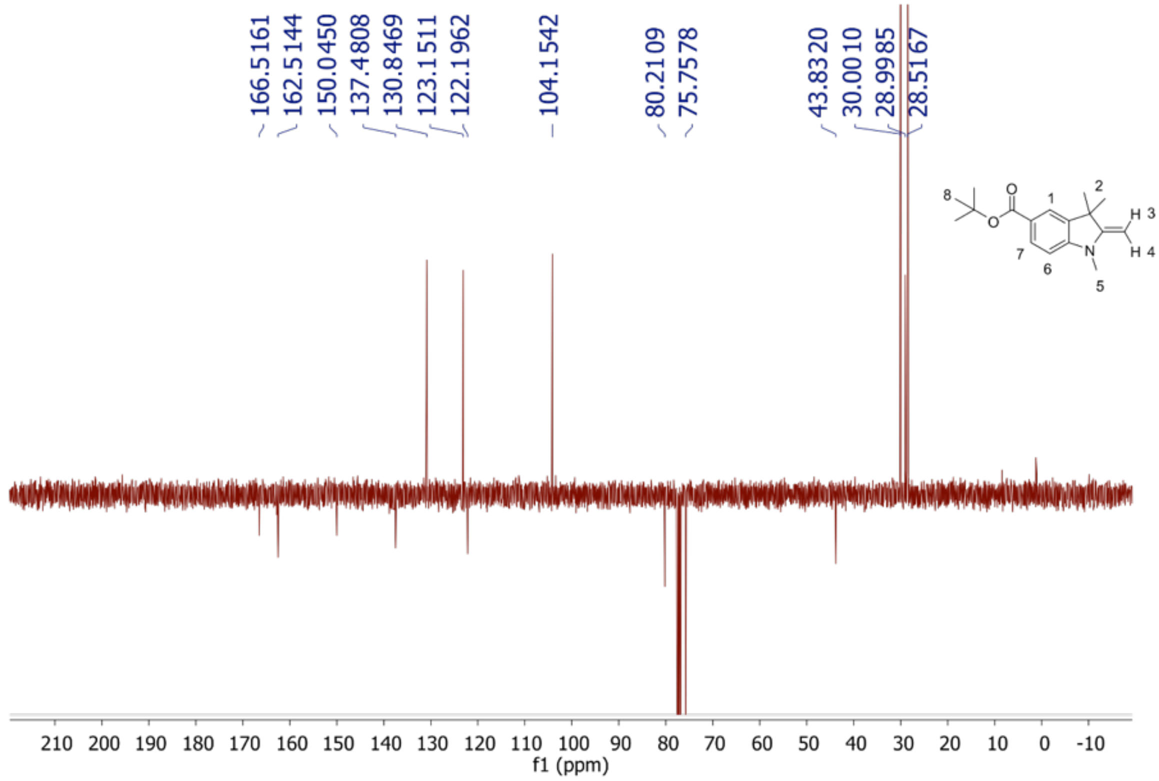

DEPT ($CDCl_3$, 101 MHz) spectrum of *tert*-butyl 1,3,3-trimethyl-2-methyleneindoline-5-carboxylate (**4**).

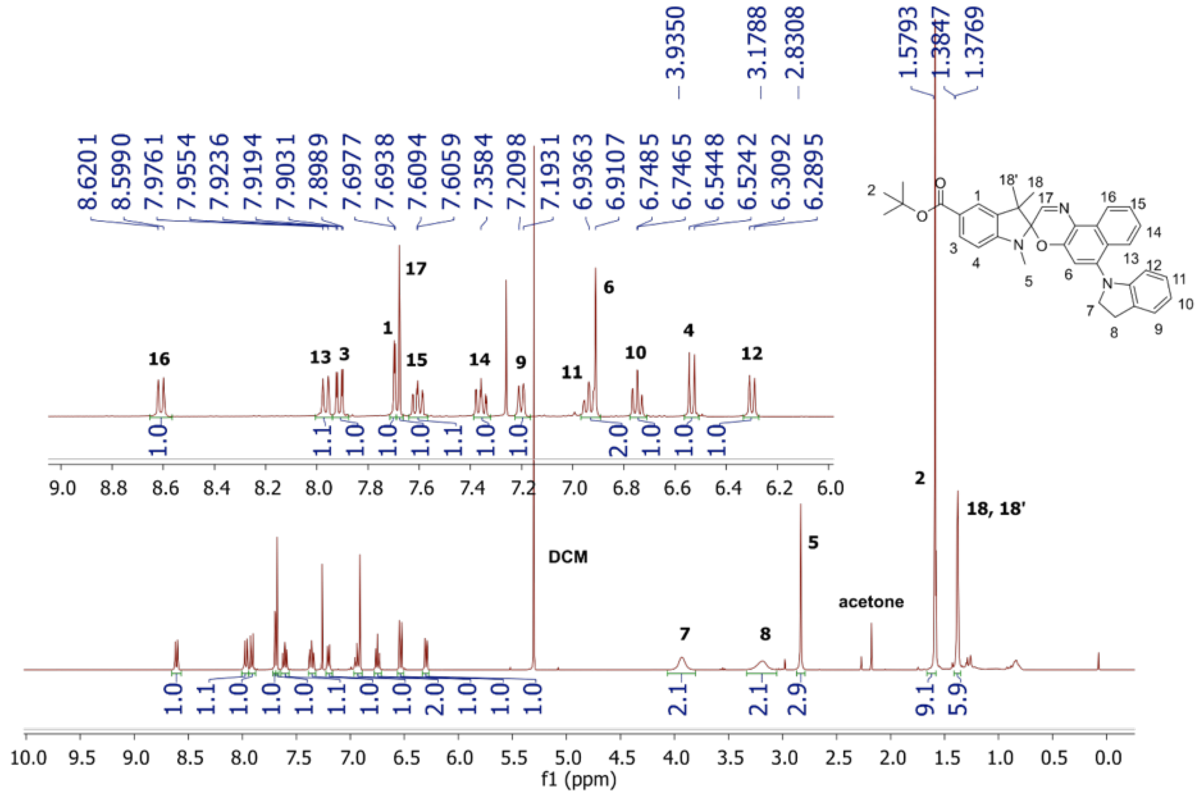

^{1}H ($CDCl_3$, 400 MHz) spectrum of *tert*-butyl 6'-(indolin-1-yl)-1,3,3-trimethylspiro[indoline-2,3'-naphtho[2,1-*b*][1,4]oxazine]-5-carboxylate (**SO**).

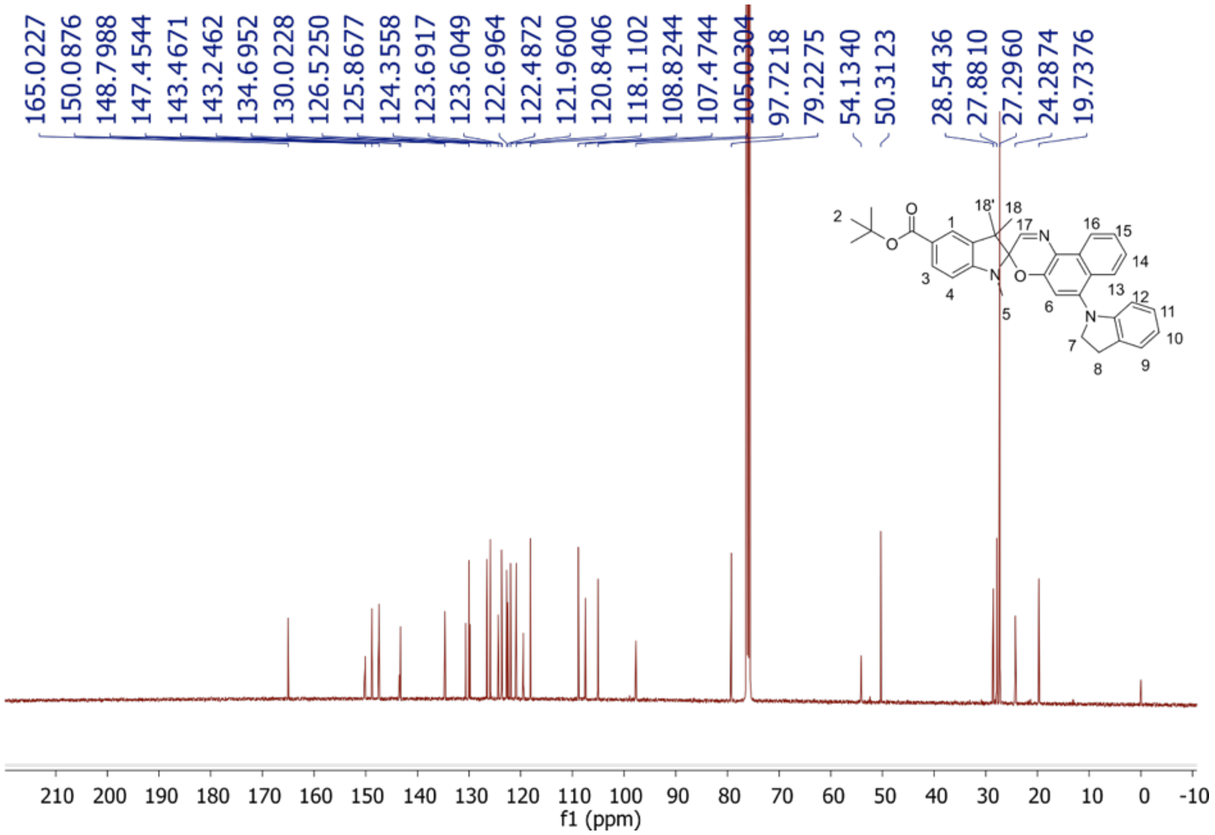

$^{13}C\{^{1}H\}$ ($CDCl_3$, 101 MHz) spectrum of *tert*-butyl 6'-(indolin-1-yl)-1,3,3-trimethylspiro[indoline-2,3'-naphtho[2,1-*b*][1,4]oxazine]-5-carboxylate (**SO**).

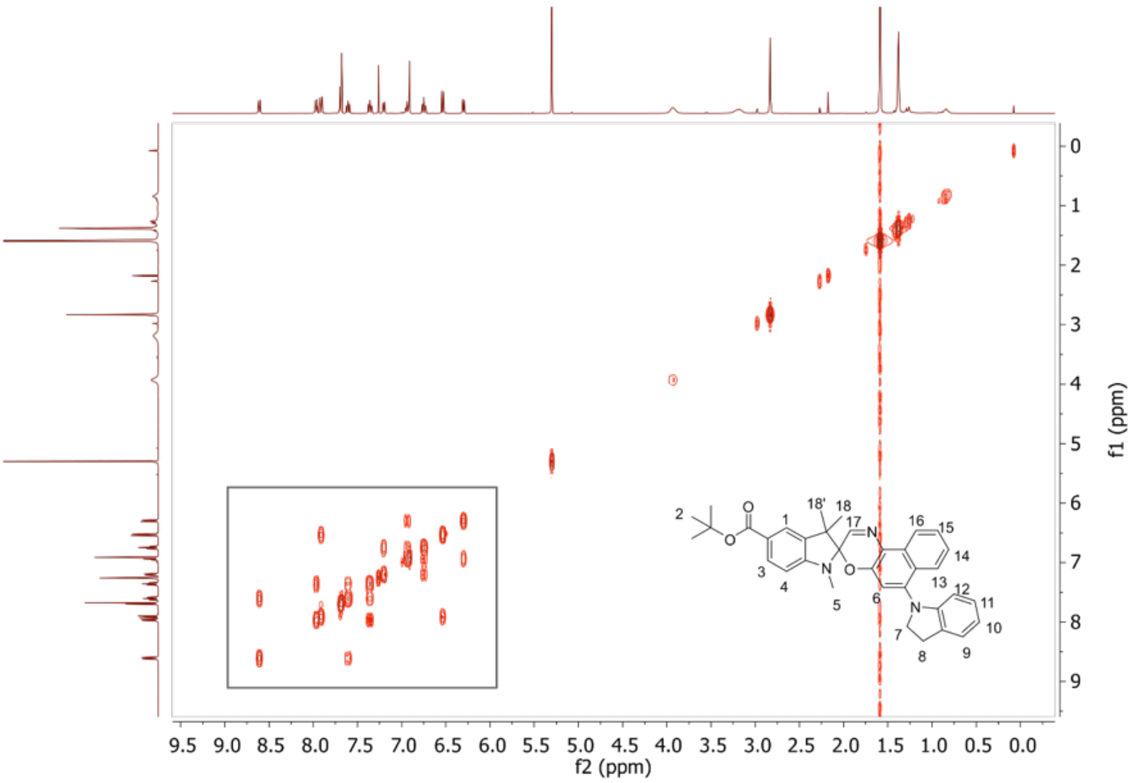

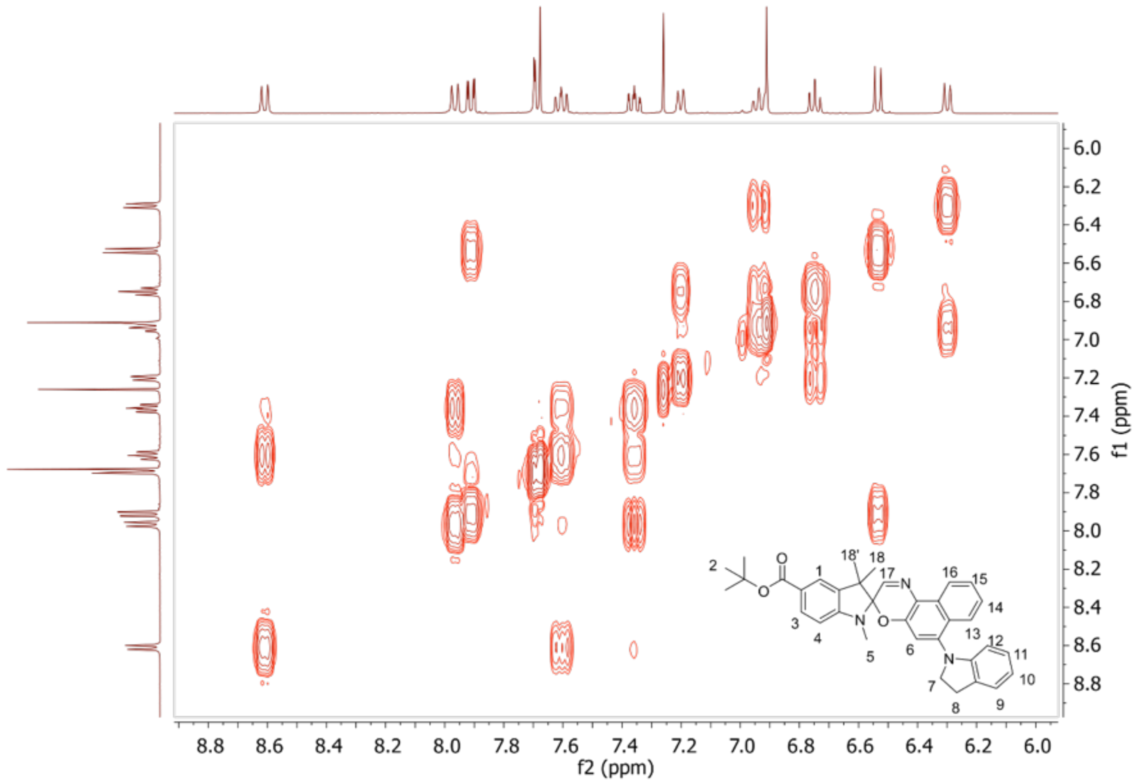

^{1}H-^{1}H COSY ($CDCl_3$, 400, 400 MHz) spectrum of *tert*-butyl 6'-(indolin-1-yl)-1,3,3-trimethylspiro[indoline-2,3'-naphtho[2,1-*b*][1,4]oxazine]-5-carboxylate (**SO**), with zoom-in of the aryl region.

	16	13	3	1	17	15	14	9	11	6	10	4	12
**16**						s							
**13**							s						
**3**												s	
**1**													
**17**													
**15**	s						m						
**14**		s				m							
**9**											s		
**11**											m		s
**6**													
**10**								s	m				
**4**			s										
**12**									s				

^{1}H-^{1}H COSY ($CDCl_3$, 400, 400 MHz) spectrum of *tert*-butyl 6'-(indolin-1-yl)-1,3,3-trimethylspiro[indoline-2,3'-naphtho[2,1-*b*][1,4]oxazine]-5-carboxylate (**SO**), COSY correlation table. s = strong, m = medium.

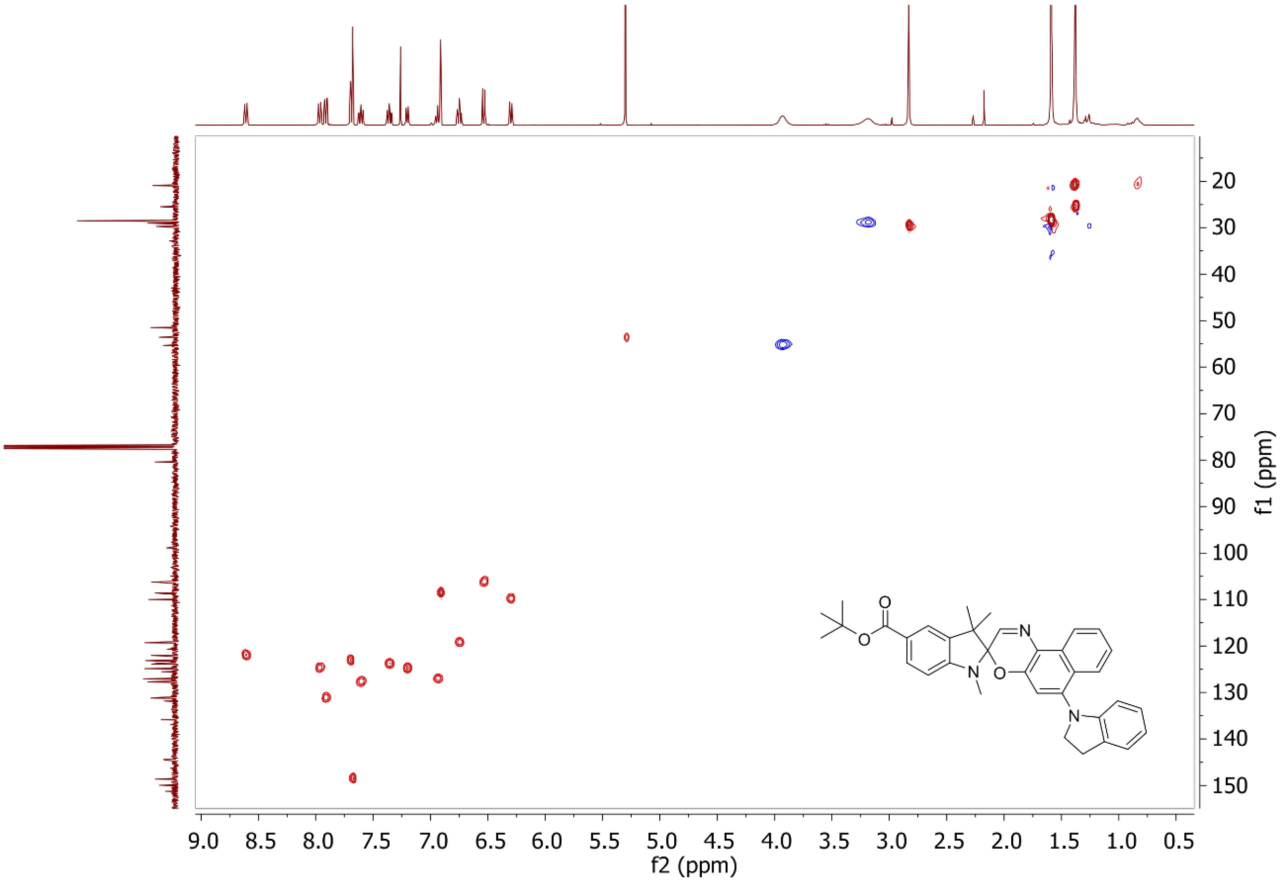

^{13}C-^{1}H HSQC ($CDCl_3$, 400, 101 MHz) spectrum of *tert*-butyl 6'-(indolin-1-yl)-1,3,3-trimethylspiro[indoline-2,3'-naphtho[2,1-*b*][1,4]oxazine]-5-carboxylate (**SO**).

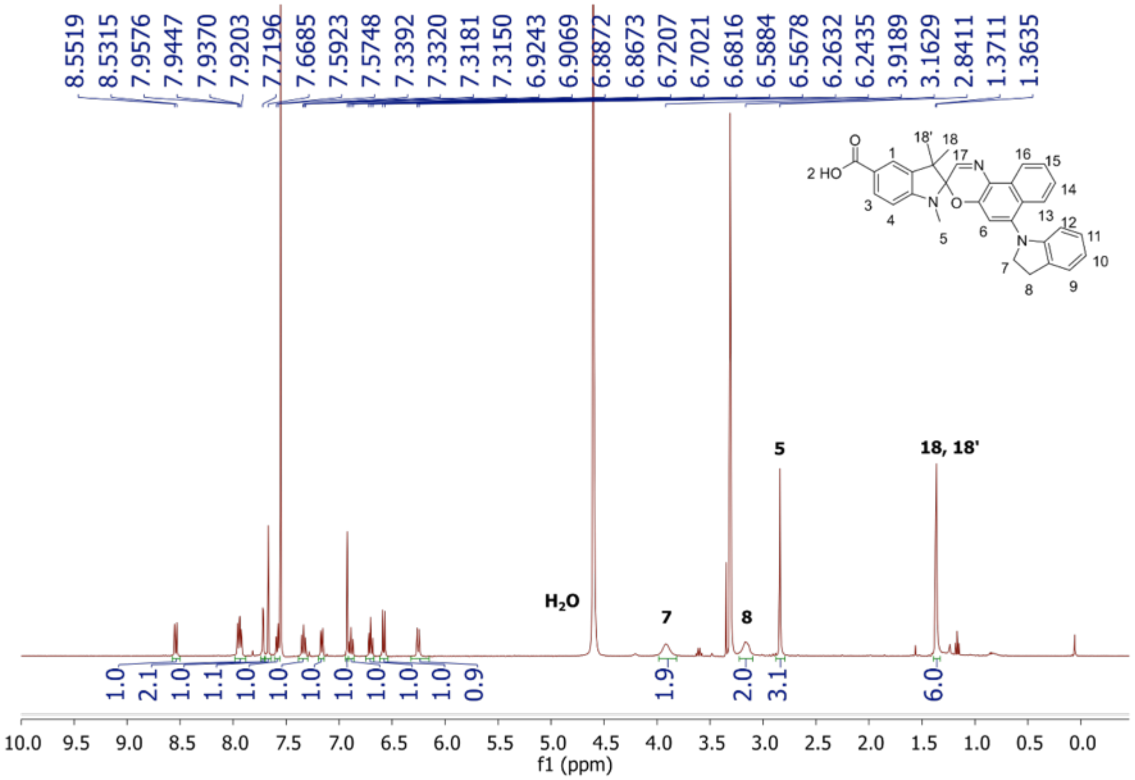

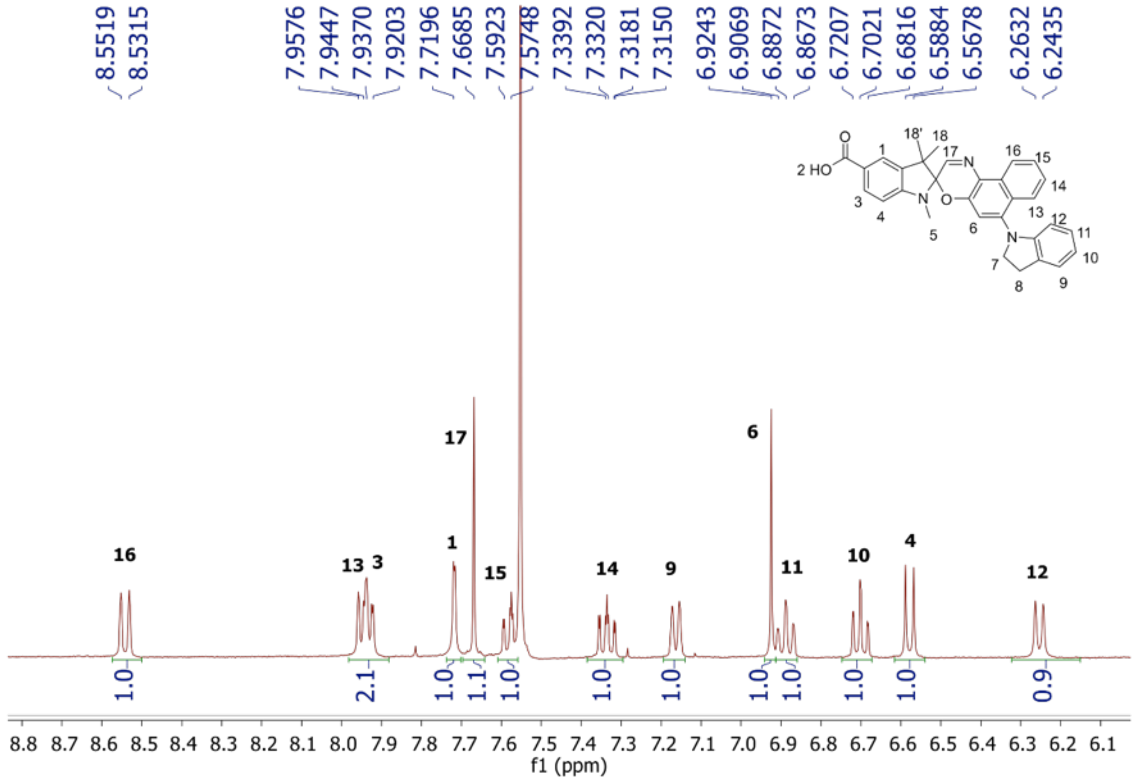

^1H (1:1 $CD_3OD/CDCl_3$, 400 MHz) spectrum of 6'-(indolin-1-yl)-1,3,3-trimethylspiro[indoline-2,3'-naphtho[2,1-*b*][1,4]oxazine]-5-carboxylic acid (**7**), and the zoom-in of the aryl region.

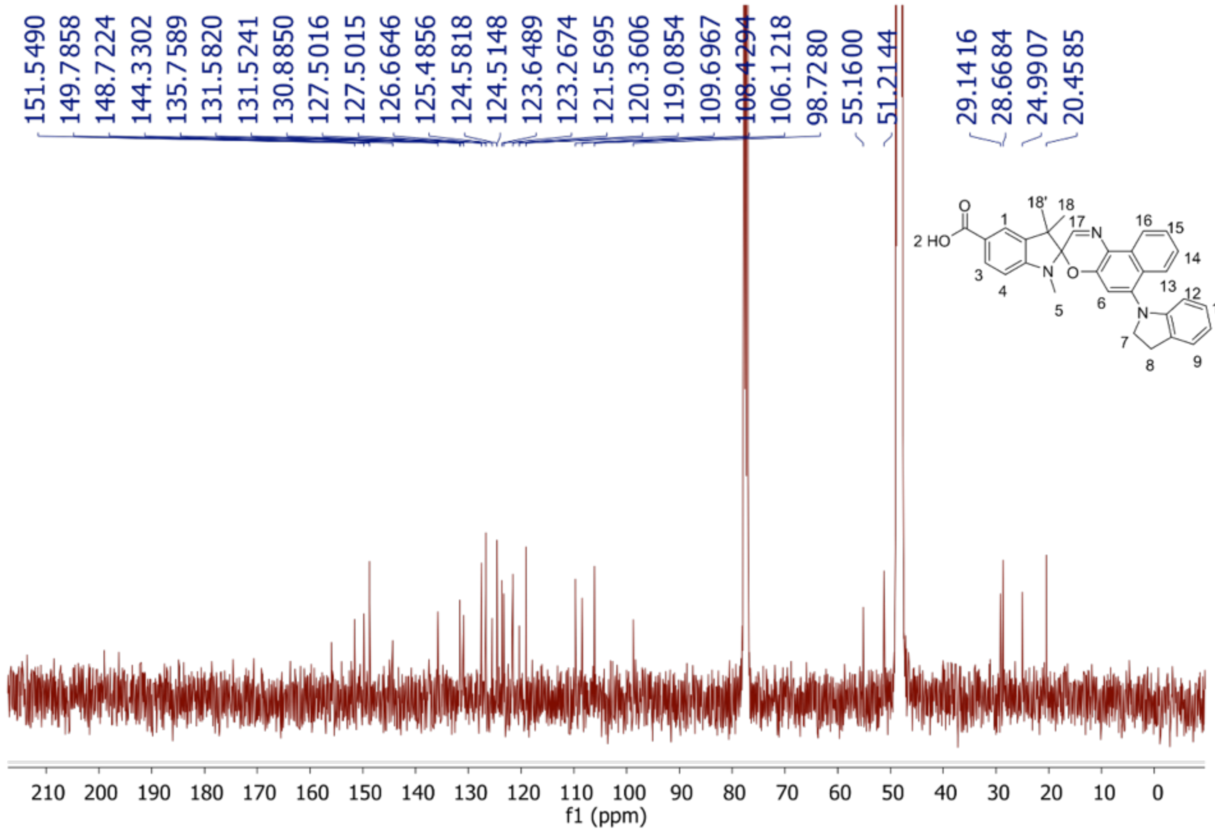

$^{13}C\{^{1}H\}$ (1:1 $CD_3OD/CDCl_3$, 101 MHz) spectrum of 6'-(indolin-1-yl)-1,3,3-trimethylspiro[indoline-2,3'-naphtho[2,1-*b*][1,4]oxazine]-5-carboxylic acid (**7**).

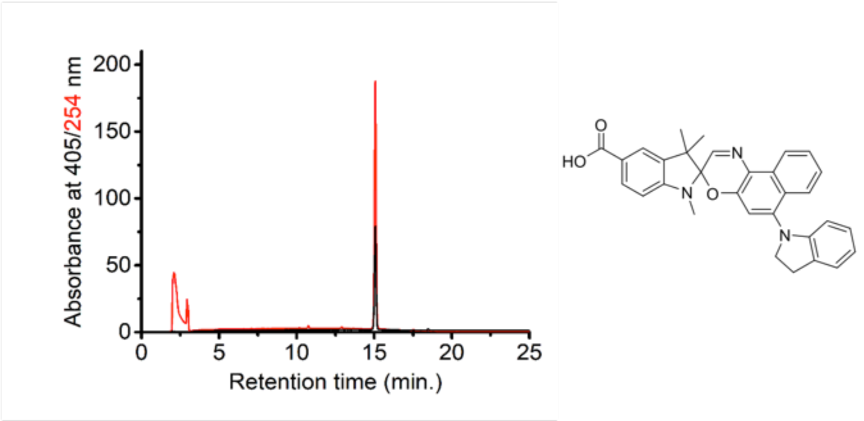

HPLC trace (reverse phase silica, 40–100% CH_3CN/0.1% TFA in H_2O over 15 min, 0.1% TFA in H_2O over 10 min) of compound **7** by monitoring absorbance at 405 nm (purity by area integration: 94%) and at 254 nm.

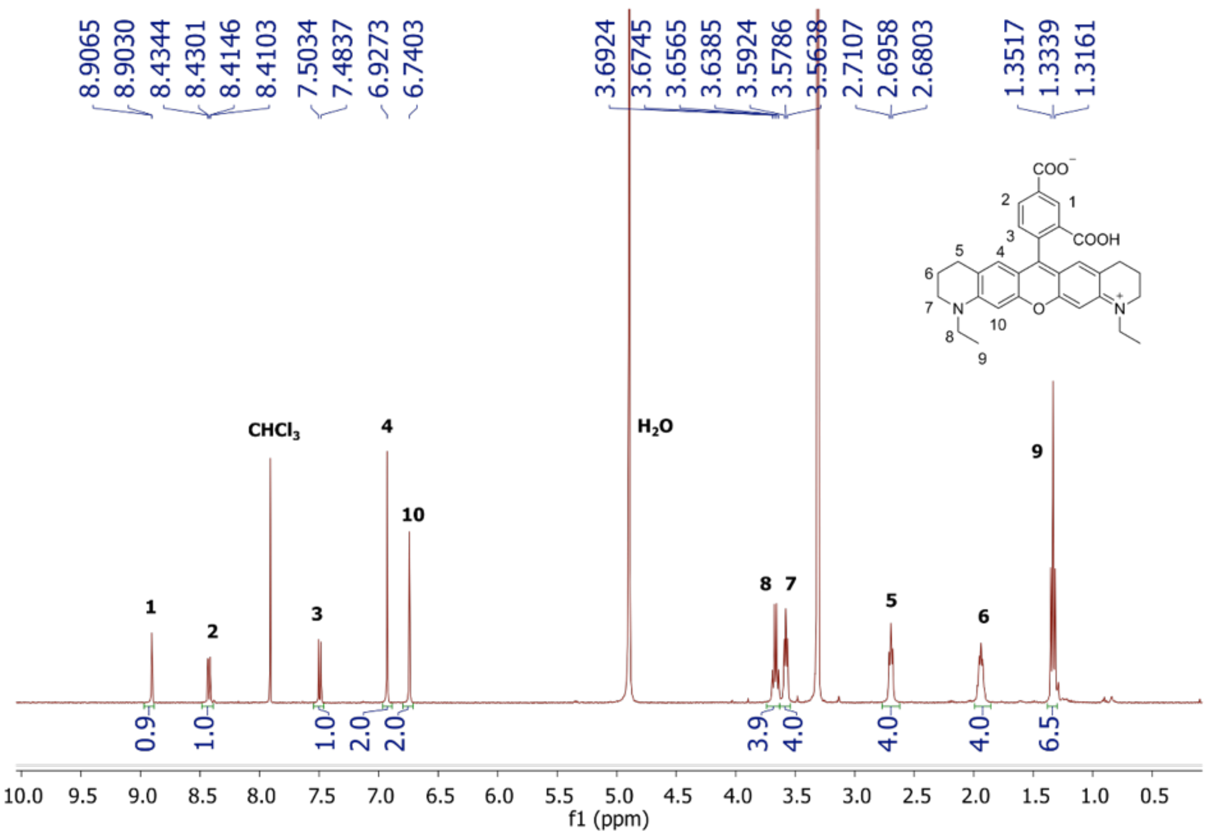

^{1}H (CD_3OD, 400 MHz) spectrum of 5'-Atto 565.

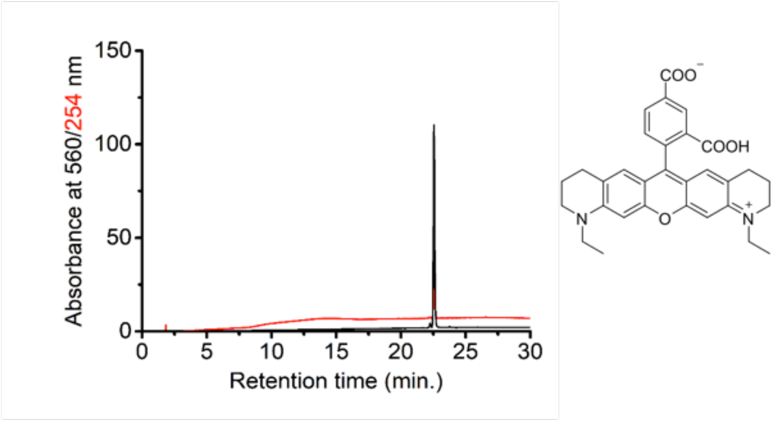

HPLC trace (reverse phase silica, 0–100% CH_3OH/0.1% TFA in H_2O over 30 min) of 5'-Atto 565 by monitoring absorbance at 560 nm (purity by area integration: 98%) and at 254 nm.

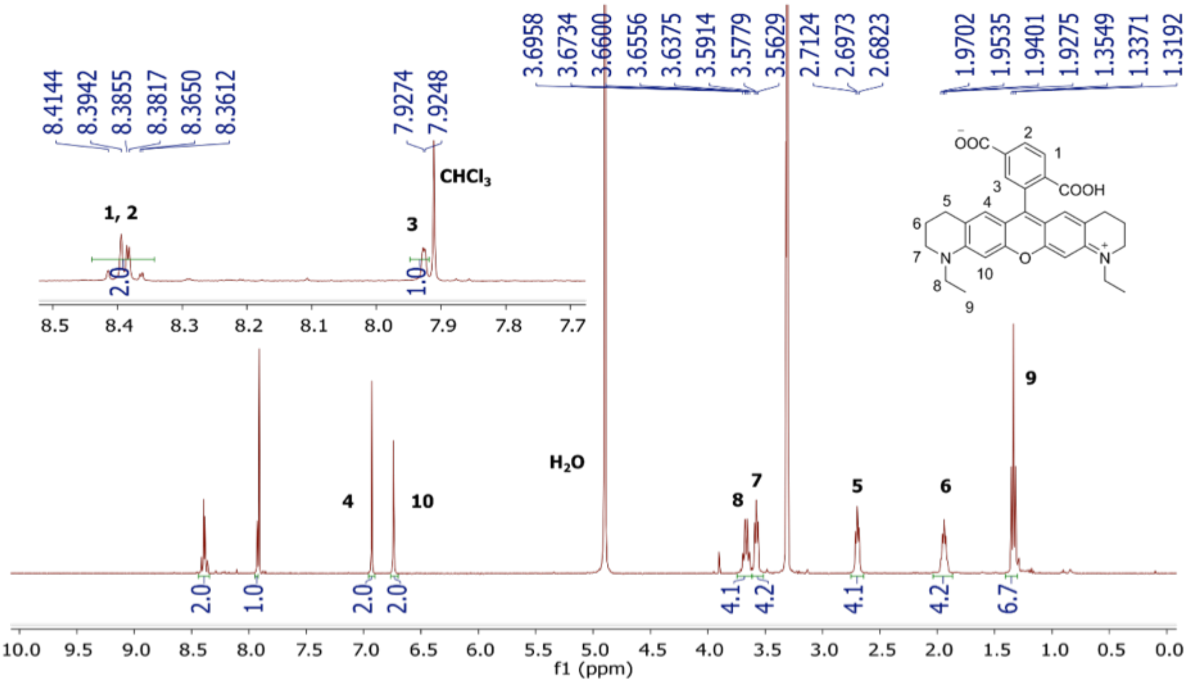

^{1}H (CD_3OD, 400 MHz) spectrum of 6'-Atto 565.

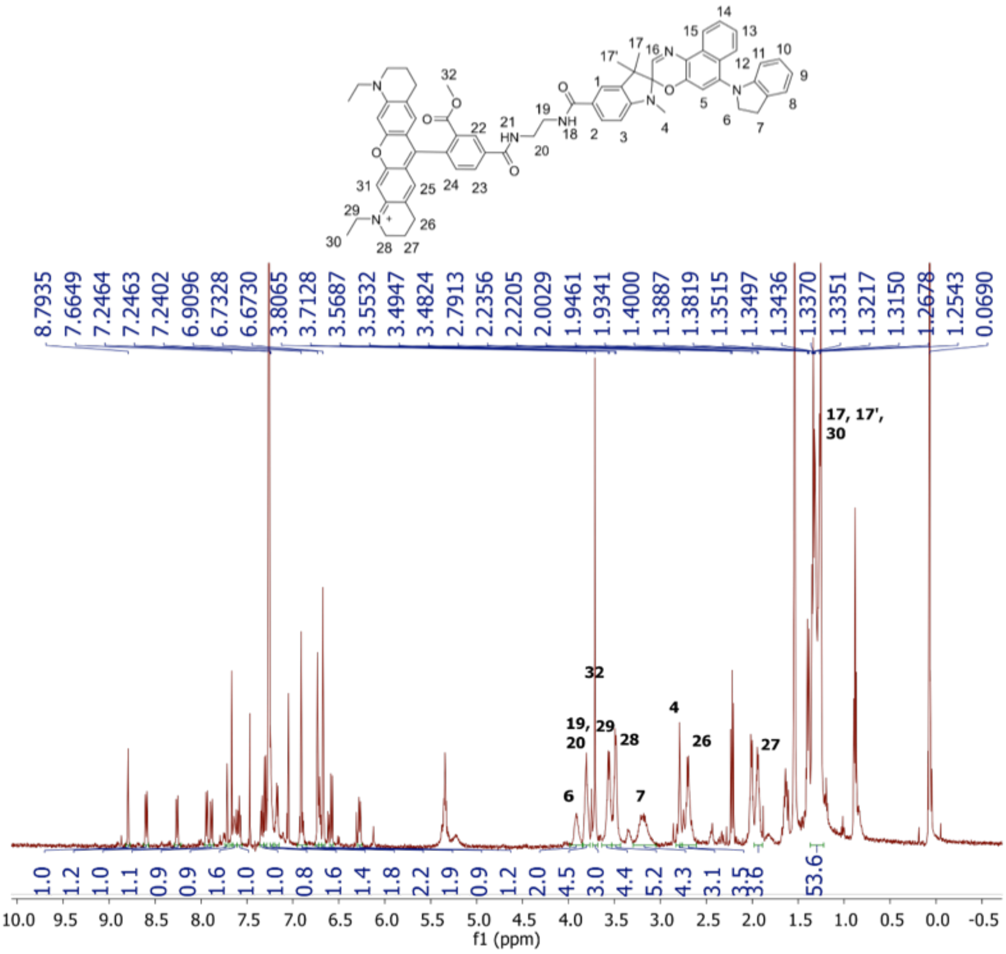

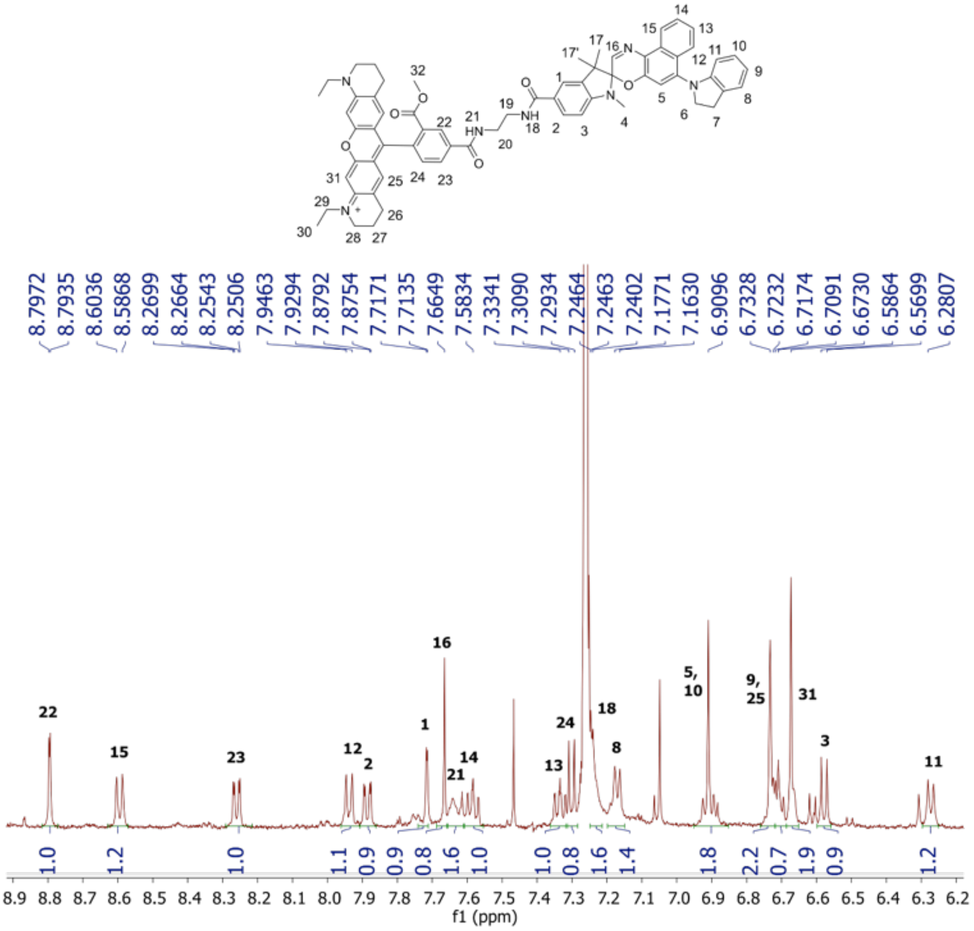

^{1}H ($CDCl_3$, 500 MHz) spectrum of **Dyad 1**, and the zoom-in of the aryl region.

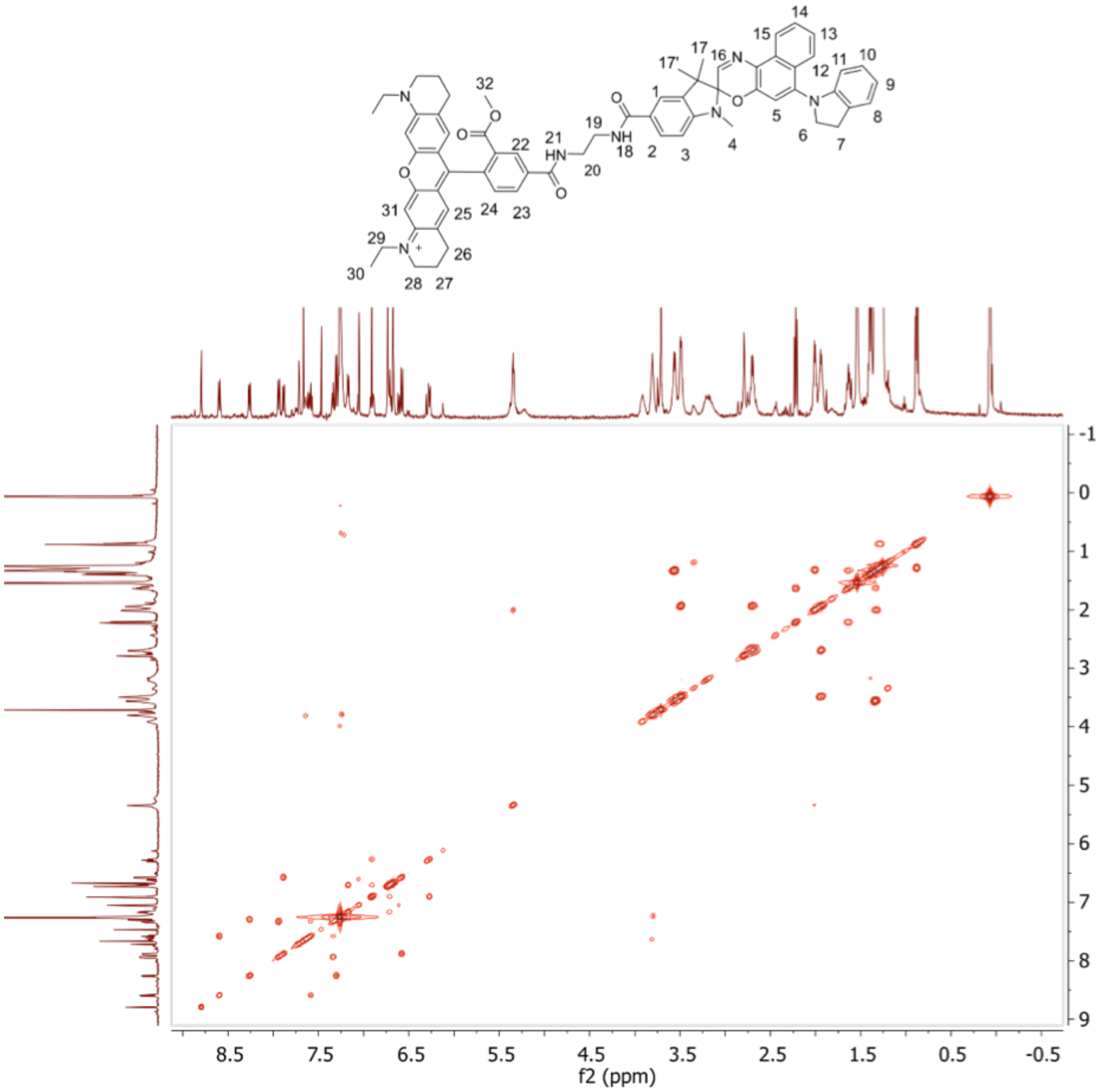

^{1}H-^{1}H COSY ($CDCl_3$, 500, 500 MHz) spectrum of **Dyad 1**

	22	15	23	12	2	1	16	21	14	13	24	18	8	5,10	9,25	31	3	11
22																		
15									s									
23											s							
12										s								
2																	s	
1																		
16																		
21																		
14		s								m								
13				s					m									
24			s															
18																		
8															s			
5,10															m			m
9,25													s	m				
31																		
3					s													
11														m				

	21	18	6	19,20	32	29	28	7	4	26	27	17, 17', 30
21				w								
18				w								
6												
19, 20	w	w										
32												
29												v.s
28											v.s	
7												
4												
26											v.s	
27							v.s			v.s		
17, 17, 30						v.s						

^1H-^1H COSY ($CDCl_3$, 500, 500 MHz) spectrum of **Dyad 1**; correlation table. v.s = very strong, s = strong, m = medium, w = weak.

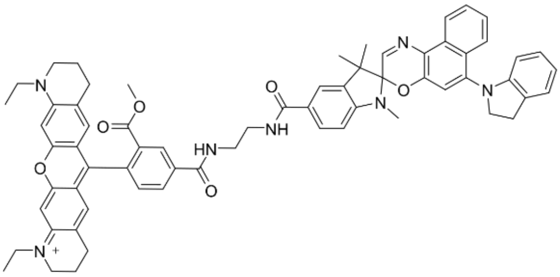

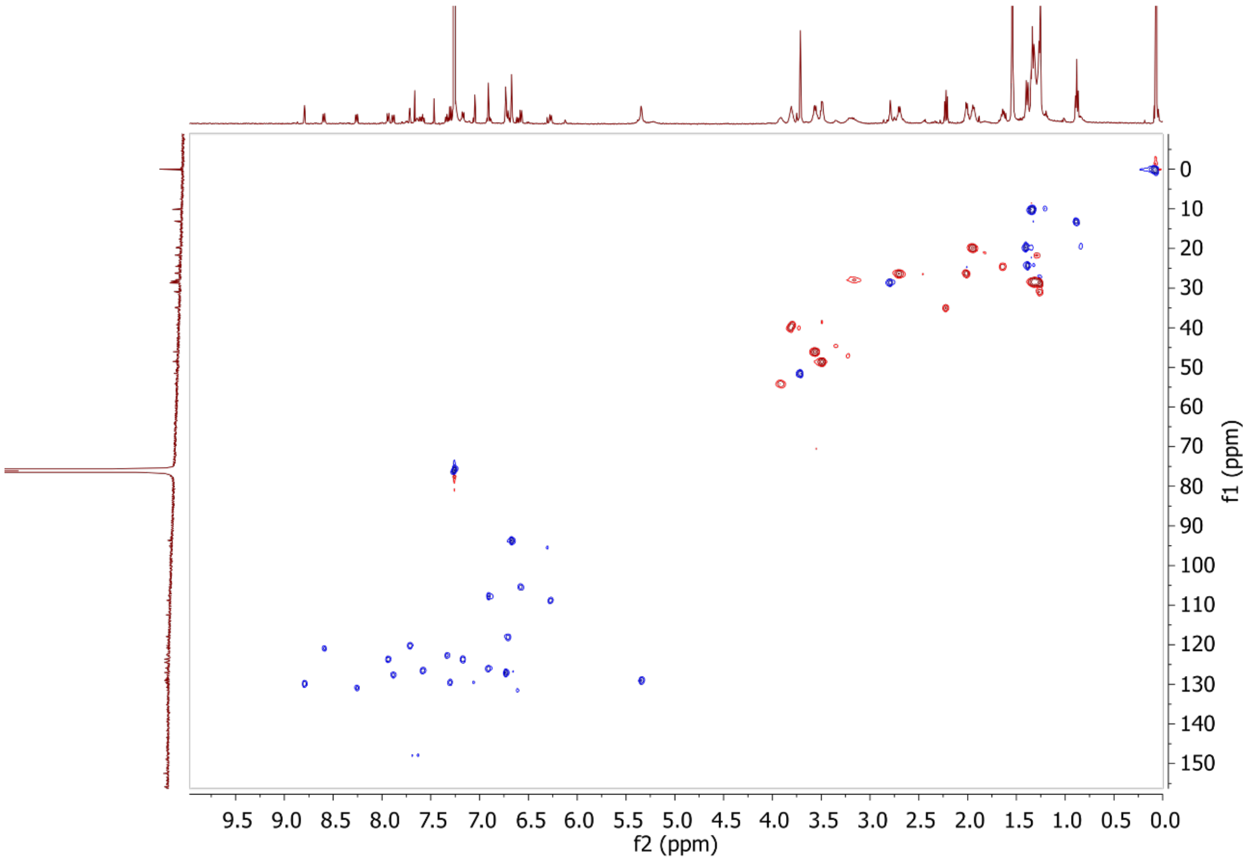

^{13}C-^{1}H HSQC ($CDCl_3$, 500, 126 MHz) spectrum of **Dyad 1**.

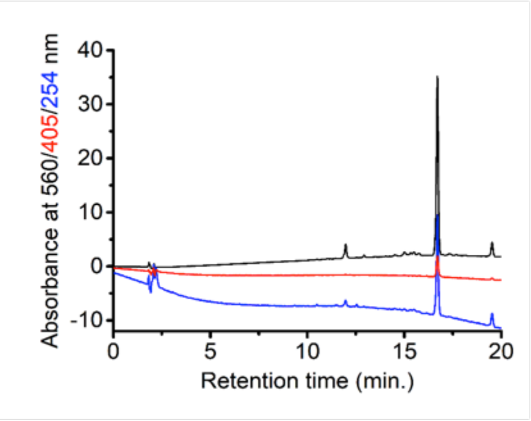

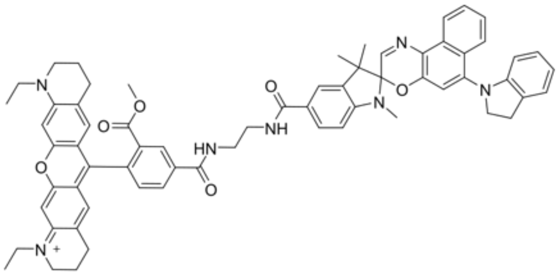

HPLC trace (reverse phase silica, 5–100% CH_3CN/0.1% TFA in H_2O over 20 min) of **Dyad 1** by monitoring absorbance at 560 (purity by area integration: 87%), at 405 nm and 254 nm.

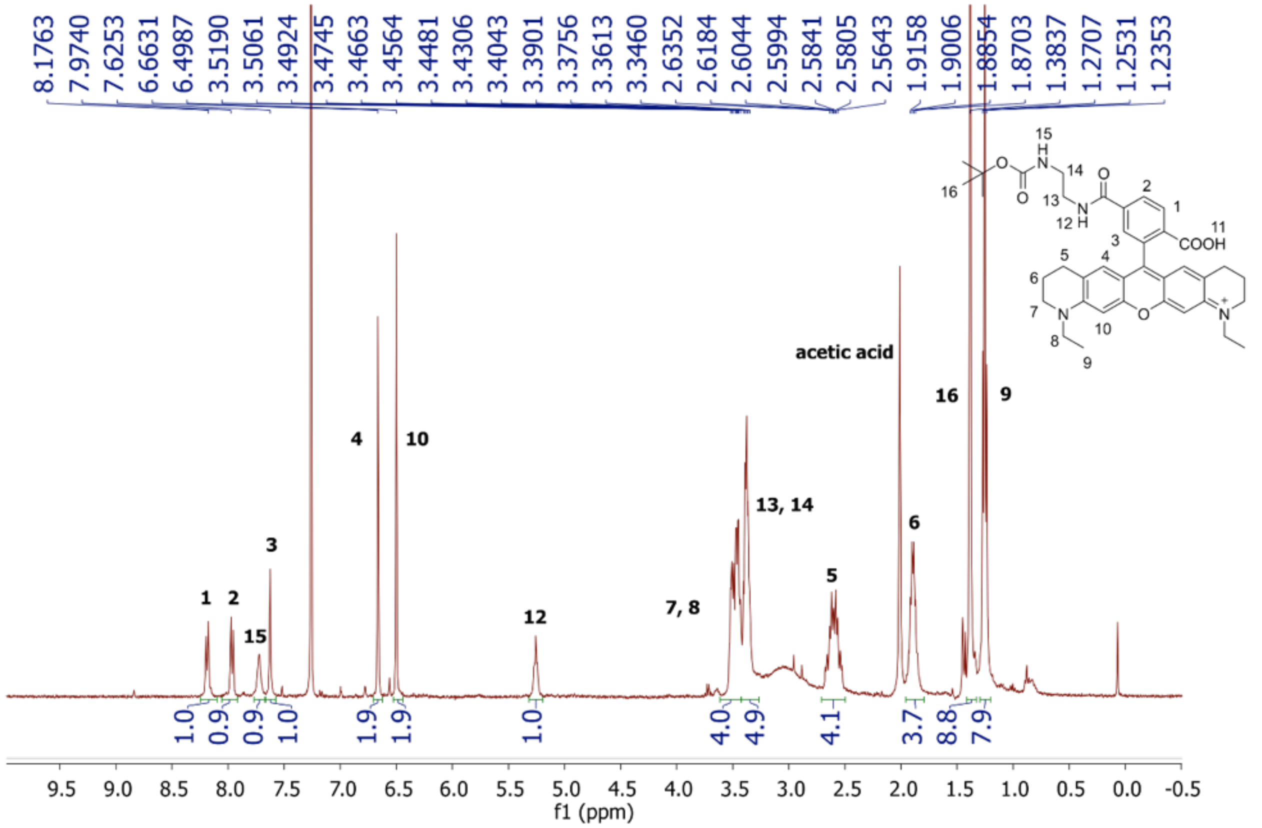

^1H ($CDCl_3$, 400 MHz) spectrum of 6-(5-((2-((*tert*-butoxycarbonyl)amino)ethyl)carbamoyl)-2-carboxyphenyl)-1,11-diethyl-3,4,8,9,10,11-hexahydro-2*H*-pyrano[3,2-*g*:5,6-*g'*]diquinolin-1-ium (**8**).

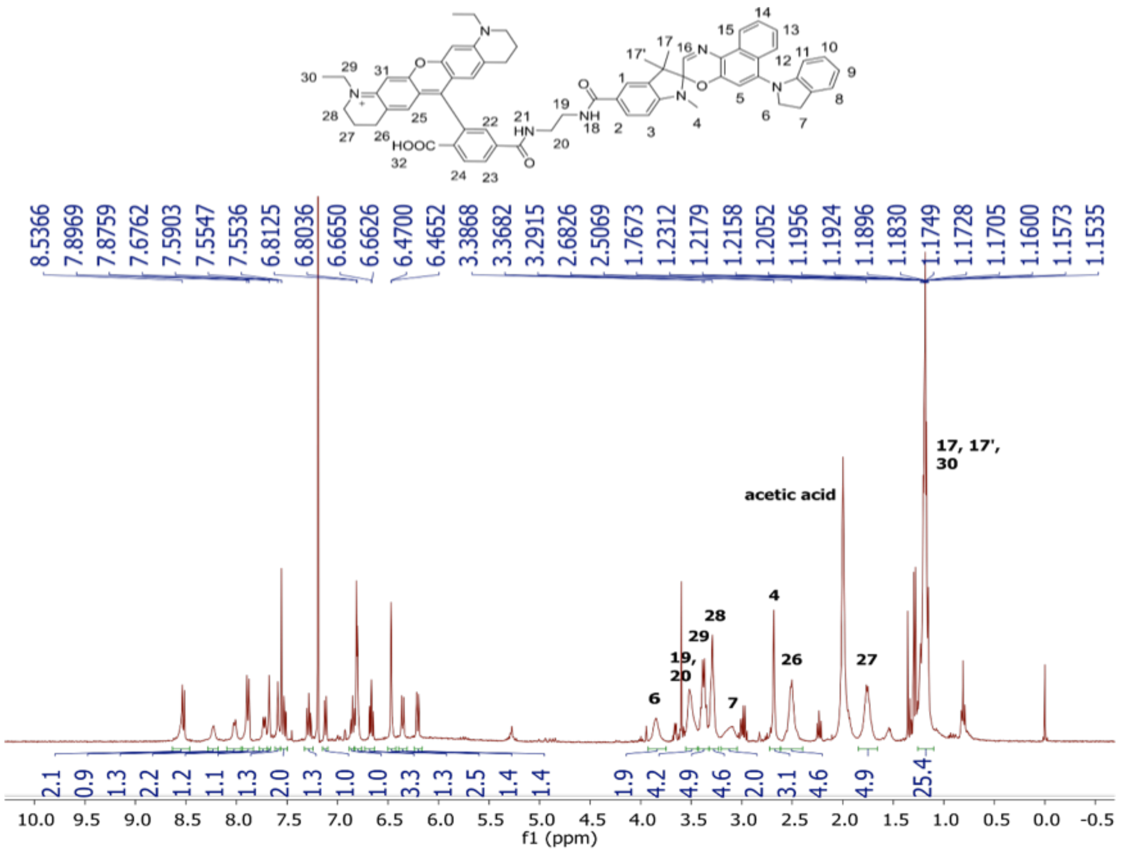

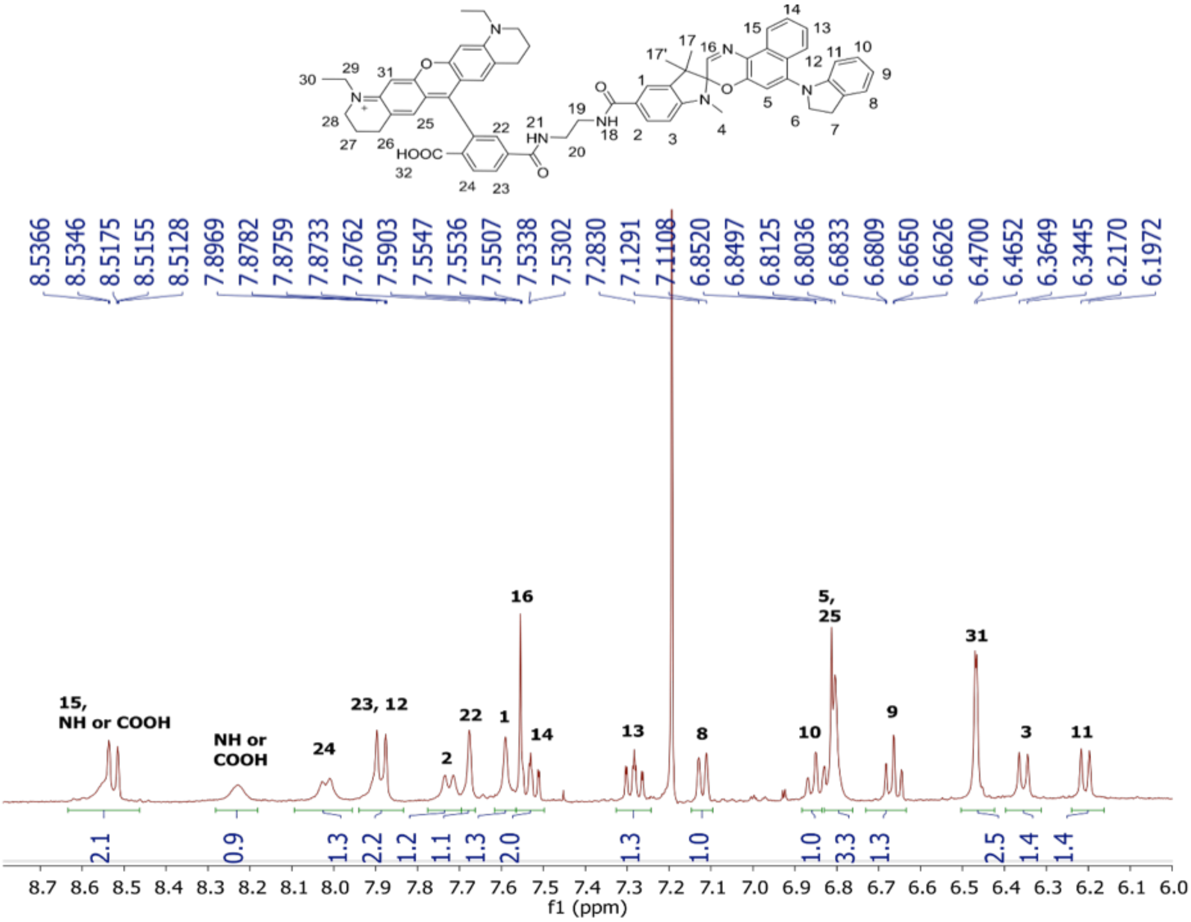

^{1}H ($CDCl_3$, 400 MHz) spectrum of **Dyad 2**, and the zoom-in of the aryl region.

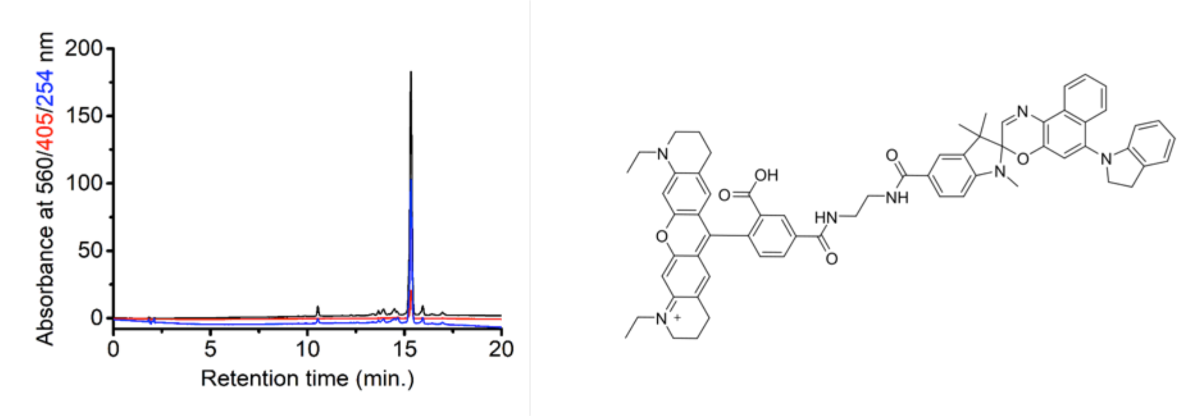

HPLC trace (reverse phase silica, 5–100% CH_3CN/0.1% TFA in H_2O over 20 min) of **Dyad 2** by monitoring absorbance at 560 (purity by area integration: 83%), at 405 nm and 254 nm.
